# Within-Couple Associations Between Communication and Relationship Satisfaction Over Time

**DOI:** 10.1177/01461672211016920

**Published:** 2021-05-24

**Authors:** Matthew D. Johnson, Justin A. Lavner, Marcus Mund, Martina Zemp, Scott M. Stanley, Franz J. Neyer, Emily A. Impett, Galena K. Rhoades, Guy Bodenmann, Rebekka Weidmann, Janina Larissa Bühler, Robert Philip Burriss, Jenna Wünsche, Alexander Grob

**Affiliations:** 1 University of Alberta, Edmonton, Canada; 2 University of Georgia, Athens, USA; 3 Friedrich-Schiller-Universität Jena, Germany; 4 Universität Wien, Austria; 5 University of Denver, CO, USA; 6 University of Toronto, Mississauga, Ontario, Canada; 7 University of Zurich, Switzerland; 8 University of Basel, Switzerland; 9 University of Bern, Switzerland

**Keywords:** couples, communication, longitudinal, relationship satisfaction

## Abstract

Relationship science contends that the quality of couples’ communication predicts relationship satisfaction over time. Most studies testing these links have examined between-person associations, yet couple dynamics are also theorized at the within-person level: For a given couple, worsened communication is presumed to predict deteriorations in future relationship satisfaction. We examined within-couple associations between satisfaction and communication in three longitudinal studies. Across studies, there were some lagged within-person links between deviations in negative communication to future changes in satisfaction (and vice versa). But the most robust finding was for concurrent within-person associations between negative communication and satisfaction: At times when couples experienced less negative communication than usual, they were also more satisfied with their relationship than was typical. Positive communication was rarely associated with relationship satisfaction at the within-person level. These findings indicate that within-person changes in negative communication primarily covary with, rather than predict, relationship satisfaction.

A large literature spanning several decades has examined couple communication as a predictor of future satisfaction (e.g., Gottman & Krokoff, 1989; Huston et al., 2001; Karney & Bradbury, 1997; Leuchtmann et al., 2019; Markman et al., 2010; Ross et al., 2019). Meta-analytic results have shown that negative interactions are associated with lower relationship satisfaction and positive interactions with higher satisfaction, cross-sectionally (Woodin, 2011) and longitudinally (Karney & Bradbury, 1995), though many individual studies have produced findings counter to this pattern. For example, Gottman and Krokoff (1989) found that husbands’ negative communication was linked with lower concurrent relationship satisfaction, but higher satisfaction in the future. Wives’ positive conflict communication, however, was associated with higher concurrent relationship satisfaction, but lower future satisfaction. Such counterintuitive findings, coupled with others finding nonsignificant links between communication and future satisfaction (e.g., Ross et al., 2019), led to doubts about the robustness of the couple communication to relationship satisfaction pathway (Karney & Bradbury, 2020).

Before concluding that these linkages are indeed weak, it is important to ensure that they have been subjected to a rigorous test that is consistent with theory. Prior work has fallen short of such a test, given that existing studies have almost exclusively focused on linkages between communication and satisfaction at the between-person level. That is, all variance in cross-sectional data necessarily reflects between-person differences, and longitudinal studies have typically focused on how between-person differences in communication (generally assessed only once, at baseline) predict differences in how couples’ satisfaction changes over time. However, between-person associations between communication and relationship functioning cannot speak to the within-person associations between these constructs. This is a critical issue because the interplay between communication and relationship satisfaction is also conceptualized as a within-couple process: If a couple communicates more positively (or negatively) than is typical for them, their satisfaction would be expected to change in the future. Accordingly, studies that specifically examine these associations at the within-person level are needed to fully test the key tenets of the theoretical models that have long guided the field of relationship science. This article uses data from three longitudinal studies, providing a rigorous test of whether within-couple changes in communication predict within-person changes in relationship satisfaction.

## Background

### Theoretical Perspective

Applications of behavioral theory to intimate relationships have guided couple research and practice since the 1970s. The core contention of behavioral models, including social learning and social exchange theories, is that couples’ communication quality affects their subjective evaluations of their relationship (e.g., satisfaction; Bandura, 1977; Gottman, 1979; Karney & Bradbury, 1995; Koerner & Jacobson, 1994; Markman, 1979; Wills et al., 1974). Positive communication patterns enhance relationship quality, whereas the accumulation of negative exchanges erodes couples’ satisfaction. Couple communication has also figured prominently in integrative models of couple relations such as the vulnerability-stress-adaptation model (VSA; Karney & Bradbury, 1995), which contends that individual characteristics and contextual factors shape relationship satisfaction via their effects on adaptive processes, which include communication patterns. Similarly, the intimacy process model (Reis & Shaver, 1988) posits that effective communication patterns lead to the experience of intimacy, which includes several hallmarks of a satisfying partnership such as feeling cared for, understood, and validated by one’s partner. This notion that communication is an important factor affecting relationship satisfaction has also served as the foundation of widely used couple therapy (Epstein & Baucom, 2002; Jacobson & Margolin, 1979) and prevention (Markman et al., 1994) programs. Indeed, Johnson and Bradbury (2015) concluded “the impact of social learning theory on the science of predicting, preventing, and treating marital dysfunction cannot be overstated” (p. 19).

In accordance with early work in the behavioral tradition (e.g., Gottman, 1979; Gottman & Krokoff, 1989; Margolin & Wampold, 1981; Markman, 1979) and the majority of the literature on couple communication in subsequent decades (for reviews, see Gottman & Notarius, 2000; Heyman, 2001; Woodin, 2011), we focus specifically on conflict or communication during problem-solving situations. In addition, we aggregate specific communication behaviors into overall positive (e.g., demonstrating interest, clarifying one’s position) and negative (e.g., being critical, withdrawing) communication dimensions, as the most frequent approach in the literature has been to examine aggregated positive and negative communication (Woodin, 2011).

### Previous Research Examining Within-Person Linkages Between Communication and Relationship Satisfaction: Review and Critique

To our knowledge, only three studies have sought to explicitly examine within-person associations between couple communication and relationship satisfaction with repeated-measures longitudinal data. The first study of these three drew on four waves of observational data gathered across the first 3 years of marriage among socioeconomically disadvantaged newlywed couples (Nguyen et al., 2020) to examine whether within-person fluctuations in positive (e.g., being affectionate), negative (e.g., showing contempt), and effective (e.g., generating solutions) observed communication covaried with concurrent fluctuations in relationship satisfaction and whether these results were moderated by stress. Results revealed husbands and wives were more satisfied than was typical for them at times when positive communication was higher than normal. Wives experienced lower than usual relationship satisfaction when negative communication was higher than normal. Husbands also experienced less satisfaction when negative communication was higher than normal, but only at times when they were also experiencing higher than average levels of stress. Similarly, within-person increases in effective communication were only linked with higher than typical relationship satisfaction for couples with higher overall stress levels.

Two other studies examined lagged associations between communication and satisfaction. Using the same sample as Nguyen et al. (2020), Lavner and colleagues (2016) found no consistent pattern linking negative, positive, or effective communication with later relationship satisfaction. Among the 36 longitudinal communication-to-satisfaction paths estimated with all four waves of data (9-month lags), only seven were significant, and all cross-lagged links were small in magnitude. Follow-up analyses examining associations between only the first and last waves (a 27-month lag) revealed that communication never predicted future satisfaction over this longer time interval. In contrast, Johnson et al. (2018) examined self-report data from five annual waves of the German Family Panel study (these data are also used in this study) and found consistent cross-lagged paths from communication to relationship satisfaction. For men and women, more frequent conflict and withdrawal from either partner predicted lower relationship satisfaction in the future, whereas each partner’s positive communication predicted higher satisfaction.

These studies represent important steps in attempting to understand within-person associations between communication and satisfaction, but they are not without limitations. Nguyen et al. (2020) used multilevel modeling to focus on within-person associations, but did not examine lagged links between these constructs. Lavner et al. (2016) and Johnson et al. (2018) used the autoregressive cross-lagged panel model (CLPM; Campbell, 1963), which has recently been critiqued for failing to disaggregate between- and within-person sources of variance (Berry & Willoughby, 2017; Hamaker et al., 2015; Mund & Nestler, 2019). This failure in the CLPM produces estimates that are, in most circumstances, a “mishmash” of between-person differences and within-person changes that may not reflect true directional associations among the variables under investigation. Accordingly, the limitations of these studies warrant additional investigation of the within-person communication-satisfaction associations across time.

Alternative analytic approaches are able to partition variance into between- and within-person components while also considering time-ordered associations between constructs. Autoregressive latent trajectory model with structured residuals (ALT-SR; Curran et al., 2014), which we employ in this study, is one such method. ALT-SR modeling allows for the explicit examination of between-person associations among the initial levels of, and rates of change over time in, communication and relationship satisfaction (random intercepts and slopes) as well as the longitudinal within-person associations between communication and relationship satisfaction captured in their residual variance at each time point. Examining longitudinal communication-to-satisfaction linkages in such a manner is important because results obtained from this approach can differ quite markedly from those obtained from the CLPM (see Berry & Willoughby, 2017, for an example with spanking and child aggression).

### The Present Study

This research explores longitudinal within-person linkages among positive and negative couple communication and relationship satisfaction. Specifically, we focus on whether deviations from one’s own average positive and negative communication (examined separately) predict future deviations in one’s own and one’s partner’s relationship satisfaction. We also consider the reverse pathway: whether within-person deviations in one’s own relationship satisfaction predict future changes in one’s own and one’s partner’s communication. Previous empirical work found varying levels of support for this possibility (Johnson et al., 2018; Lavner et al., 2016), but it has been alluded to in earlier theoretical work; for example, the VSA model (Karney & Bradbury, 1995) argues that “judgments of marital quality will affect how spouses contend with and resolve various difficulties and transitions” (p. 23). The use of ALT-SR modeling in this study allows for the simultaneous consideration of both lagged within-person pathways, as well as concurrent within-person associations: whether deviations in couple communication are linked with deviations in relationship satisfaction at the same time point (as in Nguyen et al., 2020). The analyses also include the cross-sectional and longitudinal between-person associations between communication and relationship satisfaction, given that modeling these between-person associations is necessary to ensure that the within-person results truly capture within-person effects (Curran et al., 2014). The between-person results are presented and interpreted in the Supplemental Materials because they are not the focus of this study.

We address our research questions with data from three longitudinal studies of couples. Study 1 gathered self-report data on negative communication and relationship satisfaction from a diverse sample of unmarried couples in their 20s and 30s assessed 5 times at 4-month intervals. Study 2 used observational and self-report methods to measure negative and positive couple communication and self-reported satisfaction in a sample of couples assessed annually across 5 years. Study 3 included self-report data of positive communication, negative communication, and relationship satisfaction from a national sample of couples assessed annually across 5 years. A recent review of the literature concluded that little is known about optimal time lags for studying relationship development (Karney & Bradbury, 2020); the use of 4-month and annual assessment intervals provides insight into whether within-person fluctuations in communication predict changes in satisfaction across these spans of time.

## Method

### Study 1

#### Procedures

The first five waves of data from the Relationship Development Study (RDS; Rhoades et al., 2010, 2012) were analyzed in Study 1. The RDS is a longitudinal study comprising a national sample of 1,294 young adults between the ages of 18 to 34 years in nonmarital mixed-sex intimate partnerships of at least 2-month duration at baseline. The sample was recruited in 2007 to 2008 through a calling center that used a targeted-listed telephone sampling strategy to call households in the contiguous United States. At baseline, a sample of 1,294 focal participants was recruited. Partners were invited to join the RDS for a subset of randomly selected focal participants (*n =* 642), resulting in a sample of 316 couples who provided information about relationship satisfaction and communication in at least one wave of data. Survey data were gathered at 4-month intervals across Waves 1 through 5 (spanning 16 months) through paper-and-pencil questionnaires sent by mail to each partner. This study received ethics approval from the University of Denver institutional review board.

#### Participants

Data from 316 couples who provided information on negative couple communication (positive conflict communication was not assessed) and relationship satisfaction were used in this study. Couples had been in their relationship for an average of 3.14 years (*SD* = 2.86) at baseline. The RDS recruited unmarried couples; 41.8% of couples were initially cohabiting and 22.5% were engaged to be married. Across the 16-month duration of this study, 16.1% of couples began cohabiting and 14.8% got married. At baseline, male partners were 26.96 years old (*SD* = 6.51), on average, and female partners were 24.80 years old (*SD =* 4.90). Approximately 30% (30.7%) of couples were raising children and, of those, 13.5% of couples were raising at least one child resulting from their current relationship. More than half of the male (58.5%) and female (52.9%) partners had earned a high school diploma or General Education Development (GED) as their highest education credential and 27.5% of male partners and 28.4% of female partners had earned a bachelor’s degree or higher. Regarding annual income, 46.6% of male partners and 65.2% of female partners reported earning less than US$20,000 per year.

#### Measures

Descriptive statistics and correlations among all study variables are available in Supplemental Table 1.

##### Negative communication

Negative communication was assessed at each wave with the six-item Communication Danger Signs Scale (Stanley & Markman, 1997; for example, “My partner criticizes or belittles my opinions, feelings, or desires” and “When we have a problem to solve, it is like we are on opposite teams”). Responses were 1 = *never or almost never*, 2 = *once in a while*, and 3 = *frequently*, and mean scores were computed. Cronbach’s alpha ranged from .79 to .97 for male partners and from .80 to .97 for female partners.

##### Relationship satisfaction

At each wave, relationship satisfaction was assessed with the four-item version of the Dyadic Adjustment Scale (Sabourin et al., 2005; Spanier, 1976). Three items (e.g., “In general, how often do you think that things between you and your partner are going well?”) were measured on a 6-point scale (0 = *never* to 5 = *all the time*), and the fourth (“Please indicate the degree of happiness, all things considered, of your relationship”) was measured on a 7-point scale (0 = *extremely unhappy* to 6 = *perfectly happy*). We used proportion of maximum scoring (Little, 2013) to set the items on a common scale ranging from 0 to 1 and mean scores were computed. Cronbach’s alpha ranged from .71 to .87 for male partners and from .80 to .91 for female partners.

### Study 2

#### Procedures

Data from all five waves of the Impact of Stress on Relationship Development of Couples and Children: Longitudinal Approach on Dyadic Development Across the Lifespan (PASEZ) study were analyzed in Study 2. PASEZ was a 5-year prospective longitudinal study that began in 2011. A total of 368 couples from three age cohorts (20–35, 40–55, and 65–80 years) were recruited through advertisements in newspapers and on the radio in Switzerland. To be included, couples had to be in their current relationship for at least 1 year and both spouses had to be fluent in German. Across all waves, data collection consisted of two parts. First, couples received questionnaires via mail to complete at home. Then, couples attended a university laboratory session (five total visits) where the couples completed additional questionnaires and participated in videotaped interaction tasks: a conflict conversation and two mutual support conversations. Given our focus on problem-solving communication, data from the conflict interaction task were used in this study. Couples received 100 Swiss Francs (CHF), approximately US$99, for participation in each wave. Ethics approval was granted by the University of Zurich.

#### Participants

A total of 368 Swiss mixed-sex couples participated in the study. Three couples were removed from the analyses due to missing video data, resulting in 365 couples included in the analyses. At baseline, couples had been in their relationship for an average of 21.05 years (*SD* = 18.01) and most were married (66.0%). The majority of couples (79.0%) were cohabiting and 65.2% had children (biological or stepchildren). In terms of age, the mean age was 47.24 years (*SD* = 18.33) for female partners, and 49.25 years (*SD* = 18.26) for male partners; 33.2% of couples were from the youngest cohort (aged 20–35 years), 34.2% were from the midlife cohort (aged 40–55 years), and 32.6% were from the oldest cohort (aged 65–80 years). Regarding education, 32.0% of women and 49.0% of men earned a university degree or higher. As for annual net income, 80.3% of women and 33.7% of men earned less than 60,000 CHF per year, 12.0% of women and 17.2% of men earned between 61,000 and 80,000 CHF, and 7.7% of women and 49.1% of men had annual net incomes greater than 80,000 CHF.

#### Measures

Descriptive statistics and correlations among all study variables are available in Supplemental Tables 2 through 5.

##### Observed positive and negative communication

Couples completed an observational conflict interaction task in the lab at each wave, providing five assessments of observed communication. Prior to the conflict interaction task, both partners independently rated how problematic 13 common couple conflict topics (e.g., child rearing, finances, chores, sexuality; Heavey et al., 1995) were in their relationship using a 4-point scale (1 = *a little problematic* to 4 = *very problematic*). Participants were also allowed to specify up to three additional problem areas. Next, the couples were asked to agree on one issue from the list which caused high tension for both partners. If partners did not agree, the examiner tried to achieve consensus on a topic that affected both partners to some extent. The couple then discussed the issue for 8 minutes while being videotaped.

To code communication behavior that spouses displayed in this interaction, an adapted German version of the SPAFF coding system (Gottman & Krokoff, 1989; German version by Bodenmann, 2011) was used. The coding system consisted of four main categories: verbal positivity, verbal negativity, nonverbal positivity, and nonverbal negativity. Verbal positivity was coded in four subcategories: interest, validation, affect/caring, and constructive communication. Verbal negativity consisted of seven subcategories: criticism, defensiveness, domineering, stonewalling, interruptions, contempt, and belligerence. The categories of nonverbal positivity and nonverbal negativity did not derive from specific predefined subcategories, but examples guided coding. Examples of nonverbal positivity included nodding, caring, smiling, laughing, kissing, hugging, or stroking. Examples of nonverbal negativity included refusal to answer, rejection, withdrawal, hostile gestures or facial expressions, head-shaking, or sarcastic laughter.

Two research assistants were trained to rate communication behavior. Each rater first practiced coding procedures with videotaped couple interactions not part of this study for at least 60 hr. At the end of the training period, Cohen’s kappa indicated that the raters had achieved an acceptable interobserver agreement (κ = .90). Each interaction sequence was rated by two raters simultaneously: one focusing on the man, the other focusing on the woman. Raters coded verbal and nonverbal categories in two separate passes. Videotapes were divided into 48 sequences of 10 s each because multiple categories could occur during the 8-min interaction. Each sequence of 10 s was then coded for the occurrence of the (sub)categories (0 = *did not occur*; 1 = *did occur*). Total scores for each partner’s positive communication and each partner’s negative communication were obtained by summing the verbal and nonverbal codes in each respective domain (e.g., negativity was the sum of verbal negativity and nonverbal negativity) across the 8-min discussion. The validity of the coding system with the current sample has been established in previous research (Kuster et al., 2015).

##### Self-report positive and negative communication

The 12-item Marital Communication Questionnaire (MCQ; Bodenmann, 2000) assessed self-reported positive and negative communication. The MCQ was designed to assess communication patterns in daily life comparable to those measured in the SPAFF coding system. Respondents were presented with a series of items and asked, “How do you deal with conflicts with your partner?” Four items assessed frequency of positive communication (e.g., “Endeavor to clarify the partner’s position” and “Listen to partner to understand better”). Eight items assessed the frequency of negative communication (e.g., “Insult partner,” “Criticize partner and address reproaches to him or her”). Mean scores were computed for each scale and responses ranged from 1 = *never* to 6 = *always.* Cronbach’s alpha for positive communication ranged from .79 to .85 for male partners and .75 to .82 for female partners, and Cronbach’s alpha for negative communication ranged from .77 to .78 for male and female partners.

##### Relationship satisfaction

The 4-item version of the Couples Satisfaction Index (CSI-4; Funk & Rogge, 2007) was used to assess relationship satisfaction (e.g., “In general, how satisfied are you with your relationship?)” Mean scores were computed and responses ranged from 1 = *not at all* to 6 = *completely*. Cronbach’s alpha ranged from .87 to .92 for male partners and .83 to .91 for female partners.

### Study 3

#### Procedures

Data from the first five waves of the German Family Panel (pairfam) study (Brüderl et al., 2015) were analyzed in Study 3. At baseline, a nationally representative random sample of 12,402 focal participants (referred to as anchors) were interviewed from three birth cohorts: adolescents (aged 15–17), young adults (aged 25–27), and adults approaching midlife (aged 35–37). Anchors in romantic relationships were asked for permission to contact their partners to participate in the study, resulting in a subsample of 3,743 intimate partner pairs. Anchors completed annual computer-assisted interviews with self-administered sections for sensitive topics and partners completed paper-and-pencil questionnaires. Anchors and partners were provided a small stipend (€10 and €5, respectively) for their participation. Additional study information can be found in the pairfam concept paper (Huinink et al., 2011) and from the study website: http://www.pairfam.de/en/. The first author received ethics approval for this analysis from the University of Alberta.

#### Participants

Data from 3,405 mixed-sex couples are used in this study, selected from the 3,743 total partnerships initially recruited. The 338 couples from the adolescent age cohort were excluded due to differences in teenage relationships compared with adult couples. At the outset of the study, couples had been in their relationship 8.79 years, on average, (*SD =* 5.70) and most were married (62.2%). Just more than a quarter were cohabiting (27.6%) and 10.2% were in a noncohabiting partnership. Among those in the young adult age cohort, male partners were 28.14 years old (*SD =* 4.18) and female partners were 25.45 years (*SD =* 2.62), on average, while in the midlife cohort, male partners were 37.90 years (*SD =* 4.14) and female partners were 35.12 years old (*SD =* 3.66), on average. In terms of parental status, 38% of couples had no children, 24.2% had one child, 27.4% had two children, and 10.4% had three or more kids. Less than 30% of male partners had earned a 4-year university undergraduate degree (28.5%) and 32.5% of female partners had earned an undergraduate degree. Median net annual household income was €30,000 (*M* = €32,628, *SD =* €16,200).

#### Measures

Descriptive statistics and correlations among all study variables are available in Supplemental Tables 6 and 7.

##### Positive communication

Participants’ positive communication was assessed in each wave with the average of two items adapted from the MCQ used to assess self-reported communication in Study 2 (Bodenmann, 2000). Participants indicated how often they exhibited the following behaviors during disagreements in the past 6 months: “Listen to and ask questions of your partner to understand better” and “Endeavor to clarify your own position to your partner.” Responses ranged from 1 = *almost never or never* to 5 = *very frequently*. Correlations between the items ranged from *r =* .42 to *r =* .50 across waves for male partners and *r =* .37 to *r =* .52 for female partners.

##### Negative communication

In all waves, four items assessed negative communication during disagreements in the past 6 months. Two items from the withdrawal scale of the Conflict Resolution Inventory (Kurdek, 1994) assessed how often participants “Remain silent” and “Refuse to talk about the subject” during relationship conflicts. Two items from the MCQ (Bodenmann, 2000) assessed how often participants “Insult or verbally abuse your partner” and “Yell at your partner” during disagreements. Mean scores were computed and responses ranged from 1 = *almost never or never* to 5 = *very frequently*. Cronbach’s alpha ranged from .64 to .69 for male partners and from .62 to .70 for female partners.

##### Relationship satisfaction

Relationship satisfaction was assessed with one item from the Relationship Assessment Scale (Hendrick et al., 1998): “All in all, how satisfied are you with your relationship?” Response options ranged from 0 = *very dissatisfied* to 10 = *very satisfied*.

### Analysis Plan

**Figure 1.**
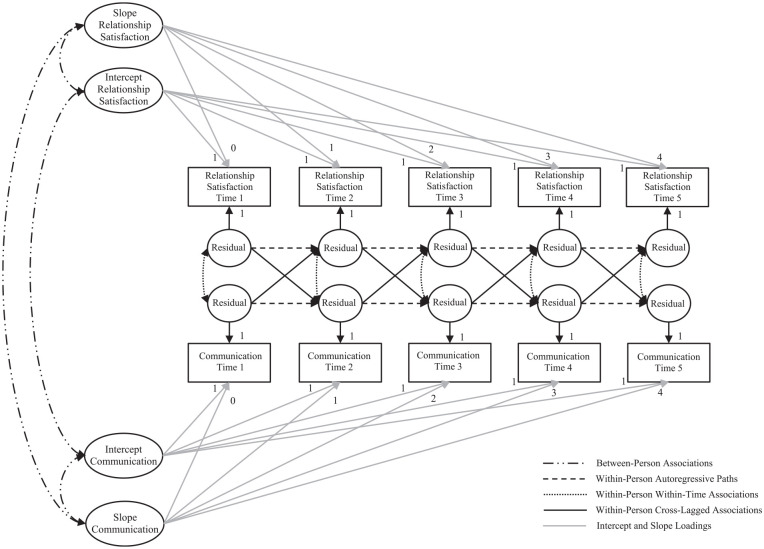
Prototype bivariate autoregressive latent trajectory model with structured residuals (ALT-SR) depicting the longitudinal interrelation of couple communication and relationship satisfaction.

In all studies, we used ALT-SR modeling (Curran et al., 2014) to examine the time-lagged association of within-person fluctuations in couple communication and relationship satisfaction. A prototype bivariate analytic model is depicted in Figure 1. In this approach, the variance in the communication and relationship satisfaction variables is partitioned into between-person differences, captured in the latent growth constructs (intercept and slope), and within-person deviations from one’s average trajectory, captured in the construct residuals at each measurement occasion. Covariances among the intercepts and slopes test the between-person associations among communication and relationship satisfaction (see the dashed dotted lines in Figure 1). These findings are discussed in the Supplement.

Within-person variation in couple communication and relationship satisfaction are examined through the addition of directional paths and covariances to the construct residuals. The autoregressive paths reflect continuity across time in how deviations from one’s average trajectory in the past influence future fluctuations (i.e., carryover effects; see the dashed lines in Figure 1). The cross-lagged paths (the solid black lines in Figure 1) shed light on our central questions: Does within-couple deviation in communication prompt future within-person change in relationship satisfaction, and does within-couple deviation in relationship satisfaction prompt future within-person change in communication? The within-time covariances capture the concurrent associations among within-person fluctuation in each construct (see the dotted lines in Figure 1).

An analytic plan recommended by Curran and colleagues (2014) guided data analysis (detailed in the Supplemental Material). In brief, we first identified the best-fitting growth curve separately for each construct (relationship satisfaction and positive and negative communication for each partner). Once we identified the shape of the trajectory, we added autoregressive paths to the construct residuals at each measurement occasion and tested the suitability of equality constraints on these paths with chi-square difference testing. Next, the ALT-SR model depicted in Figure 1 was computed using the individual construct growth curves, equality constraints were tested on cross-construct residual associations, and relationship duration was added as a control variable.

We also note that strong measurement invariance across time and partners is an assumption of longitudinal analyses involving mean structures (as is the case with growth curve analyses) to ensure that associations that emerge are true links between constructs and not due to changes in measurement (Little, 2013). Accordingly, we computed measurement invariance tests for all multi-item self-report measures (detailed in Supplemental Table 8). These analyses provide confidence that our main findings are unlikely to be attributable to inconsistent measurement.

Output files containing the syntax, complete results, and 95% confidence intervals for the final ALT-SR models are available at https://osf.io/k42ar/?view_only=79c39c2f494245db82adbfc19012c4c6.

## Results

### Initial Growth Curve Fitting

We began by fitting a series of growth models for male and female partner relationship satisfaction, positive communication, and negative communication in each study. In Study 1, a random linear slope model fit the data best for both partners’ relationship satisfaction and negative communication. Relationship satisfaction decreased slightly across the observation period for men (Cohen’s *d* = .16; *p* = .005), while women’s relationship satisfaction (Cohen’s *d =* .04) and both partners’ negative communication remained stable (Cohen’s *d*s = .00 and .01). Equality constraints were then added to the autoregressive paths for each construct. Chi-square difference testing suggested that the constraints did not substantively worsen model fit; hence, they were maintained.

In Study 2, the latent basis growth curves that captured any pattern of nonlinear change proved the best fit for every construct except male partners’ self-reported positive communication, which was best represented in the random linear slope model. Relationship satisfaction significantly decreased across time for men and women (Cohen’s *d*s = .18 and .35), as did observed positive communication (male partner Cohen’s *d* = .43; female partner Cohen’s *d =* 1.80). Observed negative communication increased significantly for men and women across the study (Cohen’s *d*s = .90 and .30). Self-reported positive communication did not exhibit significant change for male or female partners (Cohen’s *d*s = .02 and .06) but self-reported negative communication decreased for men and women (Cohen’s *d*s = .36 and .38). Equality constraints were then added to the autoregressive paths and chi-square difference testing evaluated their appropriateness. The constraints did not worsen fit in most models, except for male and female partners’ observed negative communication, so the autoregressive paths were freely estimated in those two models. The addition of autoregressive paths resulted in a Heywood case (negative slope variance estimates) for male and female partner observed negative communication and female partner observed positive communication, so the slope variances were fixed to 0 in these models and in all subsequent analyses.

For all constructs in Study 3, the latent basis growth models proved the best fit. For both partners, the slope coefficients revealed that positive communication and relationship satisfaction declined significantly over time (Cohen’s *d*s from .27 to .45), whereas negative communication increased slightly (Cohen’s *d*s = .06 and .08). Application of equality constraints on the autoregressive paths of each construct was appropriate for all constructs except for female partner positive communication.

### ALT-SR Models

Next, we computed the ALT-SR models, examined equality constraints on the longitudinal within-person paths (e.g., cross-lagged paths and within-time covariances between constructs), and then added relationship duration as a predictor of the intercepts and slopes. When computing these ALT-SR models in Study 1, Heywood cases arose: The relationship satisfaction and negative communication slope correlations were greater than one although their associations were not statistically significant. On closer inspection, the negative communication slope for male and female partners had no significant variance in these models, so they were set to zero and the models estimated normally.

Given the large number of analyses computed, we present only the focal within-person results in the manuscript in Tables 1 through 3 (the dotted and solid lines in Figure 1). Supplemental Tables 9 to 16 contain the full results for all models, including the model fit indices, between-person estimates (the dashed dotted lines in Figure 1), and the autoregressive paths between construct residuals (the dashed lines in Figure 1). We present fully standardized estimates to ease interpretability and comparability across studies, but note that some standardized coefficients differ when they were constrained to equality. In these cases, the unstandardized coefficients are equal, but the standardization calculation can result in small differences in parameter estimates (for unstandardized results, see model output at https://osf.io/k42ar/?view_only=79c39c2f494245db82adbfc19012c4c6). Each model fit the data well.

**Table 1.** Summary of Within-Person Concurrent Associations for Positive and Negative Communication and Relationship Satisfaction.

	Within-partner comm. model						Cross-partner comm. model				
Within-person results	W1	W2	W3	W4	W5		W1	W2	W3	W4	W5
Positive communication: within-person results											
Concurrent correlations for female satisfaction											
Study 1: Pos. Not assessed											
Study 2: Obs. Pos. ↔ F. Sat.	**−**.04^a^	**−**.04^a^	**−**.04^a^	**−**.03^a^	**−**.04^a^		.03^d^	.02^d^	.02^d^	.02^d^	.02^d^
Study 2: S. R. Pos. ↔ F. Sat.	.10^b^	.05^b^	.05^b^	.04^b^	.05^b^		.00^e^	.00^e^	.00^e^	.00^e^	.00^e^
Study 3: Pos. ↔ F. Sat.	.06* ^c^	.06* ^c^	.06* ^c^	.06* ^c^	.07* ^c^		.05* ^f^	.05* ^f^	.05* ^f^	.05* ^f^	.06* ^f^
Concurrent correlations for male satisfaction											
Study 1: Pos. Not assessed											
Study 2: Obs. Pos. ↔ M. Sat.	.04^g^	.05^g^	.05^g^	.03^g^	.04^g^		.25*	.23*	.02	.12	**−**.21
Study 2: S. R. Pos. ↔ M. Sat.	.09^h^	.09^h^	.08^h^	.08^h^	.10^h^		.14^j^	.08^j^	.09^j^	.07^j^	.11^j^
Study 3: Pos. ↔ M. Sat.	.05* ^i^	.06* ^i^	.06* ^i^	.05* ^i^	.07* ^i^		**−**.02	.02	.05	**−**.01	.18*
Negative communication: within-person results											
Concurrent correlations for female satisfaction											
Study 1: Neg. ↔ F. Sat.	−.39* ^a^	−.51* ^a^	−.48* ^a^	−.62* ^a^	−.45* ^a^		−.17* ^e^	−.20* ^e^	−.20* ^e^	−.29* ^e^	−.18* ^e^
Study 2: Obs. Neg. ↔ F. Sat.	−.22* ^b^	−.19* ^b^	−.11* ^b^	−.13* ^b^	−.11* ^b^		**−**.09^f^	**−**.05^f^	**−**.04^f^	**−**.03^f^	**−**.03^f^
Study 2: S. R. Neg. ↔ F. Sat.	−.24* ^c^	−.20* ^c^	–.17* ^c^	−.19* ^c^	−.19* ^c^		−.20*^g^	−.21* ^g^	−.16* ^g^	−.22* ^g^	−.19* ^g^
Study 3: Neg. ↔ F. Sat.	−.17* ^d^	–.17* ^d^	–.18* ^d^	−.19* ^d^	−.19* ^d^		−.15*	−.14*	−.11*	**−**.08	−.19*
Concurrent correlations for male satisfaction											
Study 1: Neg. ↔ M. Sat.	−.37*	–.33*	**−**.12	−.29*	−.71*		−.16* ^j^	−.19* ^j^	−.22* ^j^	−.19* ^j^	−.24* ^j^
Study 2: Obs. Neg. ↔ M. Sat.	**−**.11^h^	**−**.09^h^	**−**.06^h^	**−**.05^h^	**−**.06^h^		−.27* ^k^	−.30* ^k^	−.17* ^k^	−.20* ^k^	−.20* ^k^
Study 2: S. R. Neg. ↔ M. Sat.	−.23* ^i^	−.28* ^i^	−.20* ^i^	−.28* ^i^	−.32* ^i^		**−**.09^l^	**−**.09^l^	**−**.08^l^	**−**.09^l^	**−**.11^l^
Study 3: Neg. ↔ M. Sat.	−.12*	−.15*	−.15*	−.15*	−.25*		−.11* ^m^	−.12* ^m^	−.12* ^m^	−.11* ^m^	−.12* ^m^

*Note*: Standardized estimates.

_a–m_ Corresponding coefficients are constrained to equality. Significant effects are shown in bold for emphasis. F. = female partner; M. = male partner; Pos. = positive communication; Neg. = negative communication; Sat. = relationship satisfaction; W = wave; Obs. = observed; S. R. = self-reported; Positive communication was not assessed in Study 1.

* *p* < .05.

**Table 2.** Summary of Within-Person Cross-Lagged Effects for Positive Communication and Relationship Satisfaction.

Within-person results	Within-partner comm. model					Cross-partner comm. model				
	W1	W2	W3	W4	W5	W1	W2	W3	W4	W5
Positive communication-to-satisfaction lagged paths										
Cross-lagged paths: Pos. _W-1_ to F. Sat.										
Study 1: Pos. Not assessed										
Study 2: Obs. Pos._W-1_ → F. Sat.	–	−.05^a^	−.03^a^	−.03^a^	−.04^a^	–	.00^g^	.00^g^	.00^g^	.00^g^
Study 2: S. R. Pos._W-1_ → F. Sat.	–	−.01^b^	−.02^b^	−.02^b^	−.02^b^	–	−.05^h^	−.04^h^	−.04^h^	−.04^h^
Study 3: Pos._W-1_ → F. Sat.	–	.00^c^	.00^c^	.00^c^	.00^c^	–	.03^i^	.02^i^	.02^i^	.02^i^
Cross-lagged paths: Pos. _W-1_ to M. Sat.										
Study 1: Pos. Not assessed										
Study 2: Obs. Pos._W-1_ → M. Sat.	–	.05^m^	.04^m^	.03^m^	.06^m^	–	.10^s^	.06^s^	.06^s^	.09^s^
Study 2: S. R. Pos._W-1_ → M. Sat.	–	−.05^n^	−.05^n^	−.05^n^	−.07^n^	–	.03^t^	.05^t^	.05^t^	.07^t^
Study 3: Pos._W-1_ → M. Sat.	–	−.02^o^	−.01^o^	−.01^o^	−.01^o^	–	−.07*	.02	.05	.14*
Satisfaction-to-positive communication lagged paths										
Cross-lagged paths: F. Sat. _W-1_ to Pos.										
Study 1: Pos. Not assessed										
Study 2: F. Sat._W-1_ → Obs. Pos.	–	−.05^d^	−.08^d^	−.07^d^	−.07^d^	–	.02^j^	.04^j^	.02^j^	.02^j^
Study 2: F. Sat._W-1_ → S. R. Pos.	–	−.03^e^	−.04^e^	−.04^e^	−.05^e^	–	.00^k^	.00^k^	.00^k^	.00^k^
Study 3: F. Sat._W-1_ → Pos.	–	.00^f^	.00^f^	.00^f^	.00^f^	–	.02^l^	.02^l^	.02^l^	.02^l^
Cross-lagged paths: M. Sat. _W-1_ to Pos.										
Study 1: Pos. Not assessed										
Study 2: M. Sat._W-1_ → Obs. Pos.	–	−.03^p^	−.04^p^	−.02^p^	−.02^p^	–	.16	.08	.21*	−.25*
Study 2: M. Sat._W-1_ → S. R. Pos.	–	.03^q^	.03^q^	.03^q^	.03^q^	–	.02^u^	.03^u^	.02^u^	.03^u^
Study 3: M. Sat._W-1_ → Pos.	–	−.02^r^	−.02^r^	−.02^r^	−.03^r^	–	−.01^v^	−.01^v^	−.01^v^	−.01^v^

*Note.* Standardized estimates.

^a–v^ Corresponding coefficients are constrained to equality. Significant effects are shown in bold for emphasis. _W-1_ = preceding wave. F. = female partner; M. = male partner; Pos. = positive communication; Sat. = relationship satisfaction; W = wave; Obs. = observed; S. R. = self-reported. Positive communication was not assessed in Study 1.

* *p* < .05.

**Table 3.** Summary of Within-Person Cross-Lagged Effects for Negative Communication and Relationship Satisfaction.

Within-person results	Within-partner comm. model					Cross-partner comm. model				
	W1	W2	W3	W4	W5	W1	W2	W3	W4	W5
Negative communication-to-satisfaction lagged paths										
Cross-lagged paths: Neg. _W-1_ to F. Sat.										
Study 1: Neg._W-1_ → F. Sat.	–	−.15*^a^	−.12*^a^	−.18*^a^	−.13*^a^	–	−.08^h^	−.06^h^	−.09^h^	−.07^h^
Study 2: Obs. Neg._W-1_ → F. Sat.	–	−.06^b^	−.05^b^	−.09^b^	−.08^b^	–	.01^i^	.01^i^	.02^i^	.02^i^
Study 2: S. R. Neg._W-1_ → F. Sat.	–	.04^c^	.03^c^	.04^c^	.03^c^	–	−.16*^j^	−.11*^j^	−.14*^j^	−.10*^j^
Study 3: Neg._W-1_ → F. Sat.	–	−.03^d^	−.03^d^	−.03^d^	−.03^d^	–	−.04^k^	−.04^k^	−.04^k^	−.04^k^
Cross-lagged paths: Neg. _W-1_ to M. Sat.										
Study 1: Neg._W-1_ → M. Sat.	–	.01	.21	.02	−.24*	–	−.07^u^	−.07^u^	−.07^u^	−.07^u^
Study 2: Obs. Neg._W-1_ → M. Sat.	–	−.04^o^	−.05^o^	−.07^o^	−.09^o^	–	−.02^v^	−.02^v^	−.02^v^	−.02^v^
Study 2: S. R. Neg._W-1_ → M. Sat.	–	−.14*^p^	−.10*^p^	−.13*^p^	−.11*^p^	–	.28*	.06	−.01	−.11
Study 3: Neg._W-1_ → M. Sat.	–	−.04^q^	−.04^q^	−.03^q^	−.03^q^	–	−.05*^w^	−.05*^w^	−.04*^w^	−.04*^w^
Satisfaction-to-negative communication lagged paths										
Cross-lagged paths: F. Sat. _W-1_ to Neg.										
Study 1: F. Sat._W-1_ → Neg.	–	−.10^e^	−.09^e^	−.08^e^	−.07^e^	–	−.02^l^	−.02^l^	−.02^l^	−.01^l^
Study 2: F. Sat._W-1_ → Obs. Neg.	–	−.05^f^	−.04^f^	−.05^f^	−.04^f^	–	.03^m^	.03^m^	.03^m^	.03^m^
Study 2: F. Sat._W-1_ → S. R. Neg.	–	.03^g^	.03^g^	.02^g^	.02^g^	–	−.13*^n^	−.13*^n^	−.18*^n^	−.17*^n^
Study 3: F. Sat._W-1_ → Neg.	–	−.09*	.03	−.03	.07	–	−.15*	.00	.04	−.03
Cross-lagged paths: M. Sat. _W-1_ to Neg.										
Study 1: M. Sat._W-1_ → Neg.	–	−.02	.10	.25*	−.35*	–	.00^x^	.00^x^	.00^x^	.00^x^
Study 2: M. Sat._W-1_ → Obs. Neg.	–	.02^r^	.01^r^	.01^r^	.01^r^	–	−.12*^y^	−.10*^y^	−.17*^y^	−.19*^y^
Study 2: M. Sat._W-1_ → S. R. Neg.	–	−.19*^s^	−.17*^s^	−.25*^s^	−.23*^s^	–	.00^z^	.00^z^	.00^z^	.00^z^
Study 3: M. Sat._W-1_ → Neg.	–	−.01^t^	−.01^t^	−.01^t^	−.01^t^	–	−.02^aa^	−.02^aa^	−.02^aa^	−.02^aa^

*Note.* Standardized estimates.

^a–aa^ Corresponding coefficients are constrained to equality. Significant effects are shown in bold for emphasis. _W-1_ = preceding wave. F. = female partner; M. = male partner; Neg. = negative communication; Sat. = relationship satisfaction; W = wave; Obs. = observed; S. R. = self-reported.

* *p* < .05.

#### Within-person ALT-SR modeling results for positive communication and relationship satisfaction

Within-person concurrent associations between intraindividual deviations in positive communication and relationship satisfaction (the dotted lines in Figure 1) are shown in the upper portion of Table 1 (positive communication was not assessed in Study 1). In Study 2, two within-time correlations between positive communication and relationship satisfaction were significant: Male partners were more satisfied than normal at Waves 1 and 2 when female partners’ concurrent observed positive communication was higher than their average. In Study 3, most concurrent within-person associations were significant. Female partners reported higher than normal relationship satisfaction at times when their own and their partner’s positive communication was higher than normal and male partners reported higher than normal relationship satisfaction at times when their own positive communication was higher than normal. Male partners’ relationship satisfaction was not robustly associated with female partner communication; they were only significantly linked at Wave 5.

Table 2 contains the longitudinal within-person cross-lagged paths between positive communication and relationship satisfaction (the solid lines in Figure 1). There were no robust longitudinal links between intraindividual deviations in positive communication and within-person fluctuations in relationship satisfaction. Specifically, regarding the link from positive communication to future satisfaction, significant paths only emerged in Study 3 and these two paths were in different directions: Higher than normal female partner positive communication at Wave 1 predicted an intraindividual decrease in their male partner’s relationship satisfaction at Wave 2, but higher than normal female partner positive communication at Wave 4 predicted an intraindividual increase in their male partner’s Wave 5 relationship satisfaction. Regarding relationship satisfaction predicting future positive communication, two significant paths were found in the observational data from Study 2, but the coefficients were, again, in opposite directions: Higher than average male partner relationship satisfaction at Wave 3 predicted an intraindividual increase in female partner observed positive communication at Wave 4, but higher than average male partner relationship satisfaction at Wave 4 predicted an intrapersonal decrease in female partner positive communication at Wave 5.

#### Within-person ALT-SR modeling results for negative communication and relationship satisfaction

The bottom portion of Table 1 contains the within-person concurrent associations between intraindividual deviations in negative communication and relationship satisfaction (the dotted lines in Figure 1). Across Studies 1 through 3, these results revealed robust within-time associations between within-person fluctuations in negative communication and relationship satisfaction. The overall pattern showed that both partners were more satisfied than normal when their own and their partner’s reports of negative communication were less frequent than average (or conversely, both partners reported less frequent negative communication at times when they were more satisfied than average). There were three exceptions to this pattern in Study 2. Intraindividual deviations in male partner observed negative communication were not significantly associated with their own concurrent relationship satisfaction fluctuations or with their female partner’s concurrent relationship satisfaction deviations, and female partner self-reported negative communication was not significantly associated with within-person variation in male partner satisfaction.

The longitudinal within-person cross-lagged results between negative communication and relationship satisfaction (the solid lines in Figure 1) are shown in Table 3. Although not as robust as the concurrent associations, several of these longitudinal associations were significant, though no pattern was robust across all three studies. Regarding communication to later satisfaction, in Study 1, higher than average negative communication reported by female partners predicted an intraindividual decrease in their own future relationship satisfaction. In Study 2, an upward deviation in male partner self-reported negative communication predicted a future reduction in his own and his partner’s relationship satisfaction. There were no significant negative communication to relationship satisfaction associations with the observational data, however. In Study 3, higher than typical negative communication in the female partner predicted an intraindividual decrease in the male partner’s future relationship satisfaction at all lags.

Regarding satisfaction-to-negative communication associations, in Study 1, higher than average relationship satisfaction for male partners at Wave 3 predicted intraindividual increases in negative communication at Wave 4, but upward deviations in male partner satisfaction at Wave 4 predicted within-person reductions in negative communication in Wave 5. In Study 2, higher than average male partner relationship satisfaction predicted a future intraindividual decrease in female partner observed negative communication and higher than average relationship satisfaction for male and female partners predicted a future reduction in male partner future self-reported negative communication at all lags. In Study 3, higher than average female partner relationship satisfaction at Wave 1 predicted an intraindividual decrease in her own and her partner’s negative communication at Wave 2.

## Discussion

Do within-couple changes in communication predict future changes in relationship satisfaction? This notion has been foundational to theories, empirical studies, and intervention protocols aimed at understanding and improving couple relations, but the literature has yet to adequately test this fundamental question. This study tested this question by drawing on data from three longitudinal studies of mixed-sex couples comprising community-based and national samples that used observational and self-report measurements at 4-month and annual assessment intervals. The pattern of results across these three studies supports two overarching conclusions regarding the within-person associations between couple communication and relationship satisfaction. First, there was inconsistent evidence for associations between within-person deviations in communication on lagged changes in satisfaction (or for satisfaction on lagged changes in communication). Second, there was consistent evidence for concurrent within-person associations between negative communication and satisfaction (but not positive communication and satisfaction), such that couples were more satisfied than normal at times when they also engaged in less negative communication than was typical.

### Lagged Within-Couple Communication and Relationship Satisfaction Associations

We did not find consistent evidence that within-couple changes in communication prompted subsequent deviations in relationship satisfaction. Across studies, intraindividual deviations in positive communication were rarely a significant predictor of future changes in relationship satisfaction, indicating that increases in the extent to which partners expressed themselves and tried to understand their partner better were not associated with subsequent improvements in their relationship satisfaction. Changes in negative communication sometimes predicted worsening relationship satisfaction at the next measurement occasion, but no specific pattern was evident in every study. That is, despite the fact that each study found at least one set of linkages between negative communication and satisfaction in the direction expected by decades of theory, this pattern was not reliably observed and differed in specifics across the three studies. In Study 1, women’s self-reported negative communication predicted their own satisfaction. In Study 2, men’s self-reported negative communication predicted their own and their partner’s satisfaction. In Study 3, women’s self-reported negative communication predicted their partner’s satisfaction. Thus, although there were significant longitudinal within-person links between negative communication to future relationship satisfaction, the lack of cross-study support for any specific pathway casts doubt on the notion that communication is a robust, significant predictor of future changes in relationship satisfaction within couples.

Results were similar for the relationship satisfaction-to-communication within-person lagged links. As with the positive communication-to-satisfaction lagged associations, there was very little support for significant lagged satisfaction-to-positive communication linkages. There was relatively more support for significant lagged satisfaction-to-negative communication linkages; notably, there was a similar level of empirical support for the pathways from within-person changes in relationship satisfaction to future reductions in negative communication as there was from negative communication to future decreases in relationship satisfaction. Once again, however, no consistent longitudinal links emerged across studies. Broadly, such inconsistency in the within-person lagged communication/satisfaction association casts doubt on the robustness of these effects. Insofar as within-person fluctuations in couple communication and satisfaction might be linked, it is most likely these associations are due to changes in negative communication rather than positive communication. Furthermore, it is just as likely that fluctuations in satisfaction lead to deviations in negative communication as it is for negative communication to influence later relationship satisfaction.

Our ALT-SR analyses also considered between-person associations in within-person change trajectories of communication and relationship satisfaction (captured in their slopes). These analyses are not detailed in the article, due to length constraints, but are presented in the Supplemental Material (see Supplemental Table 17). These results are relevant to the cross-lagged within-person associations presented here because the pattern of longitudinal results at the between-person level mirrors those at the within-person level. Intercepts of communication and relationship satisfaction were inconsistently associated with the slope of the other construct and slope-to-slope associations were rare. Furthermore, several of the longitudinal between-person associations were in a counterintuitive direction (e.g., higher initial satisfaction was associated with a more gradual decrease in negative communication over time). Such counterintuitive between-couple findings are not without precedent (Gottman & Krokoff, 1989; Karney & Bradbury, 1997) and cast further doubt on the robustness of lagged associations between communication and relationship satisfaction.

### Concurrent Within-Couple Communication and Relationship Satisfaction Associations

The concurrent within-couple associations between communication and relationship satisfaction paint a clearer picture. Once again, positive communication was not robustly linked with relationship satisfaction across studies. Consistent positive communication/satisfaction associations emerged in Study 3, but the coefficients were small in magnitude (*r*s from .05 to .07). The concurrent links between within-couple deviations in negative communication and relationship satisfaction, however, were robust across studies: Upward deviations in one partner’s negative communication generally coincided with intraindividual reductions in relationship satisfaction for oneself and the partner. This pattern was evident in 13 out of 16 total models tested across the studies, with the primary inconsistent result coming from men’s observed negative communication not being associated with their own or their female partner’s satisfaction. These results indicate that deviations in one partner’s negative communication are likely accompanied by concurrent changes in both partners’ relationship satisfaction, while within-couple deviations in positive communication unfold independent of relationship satisfaction. These findings extend a large body of research on between-couple associations between communication and relationship satisfaction (e.g., Woodin, 2011) and contribute additional evidence regarding concurrent within-person links (e.g., Nguyen et al., 2020) by indicating that not only do more satisfied couples communicate more positively and less negatively than dissatisfied couples during conflict, but that couples are most satisfied than normal at times when they are communicating less negatively (though not more positively).

In considering these findings, it is important to contrast the results in our analysis with those of Nguyen et al. (2020) who published the only other study to our knowledge to examine cross-sectional within-person links between relationship satisfaction and communication. Nguyen et al. found fluctuations in positive and negative (for women) communication were linked with concurrent deviations in relationship satisfaction, whereas this study found the most robust evidence in support of negative communication/satisfaction links for both partners. One possibility for the discrepant findings is that communication in this study was assessed specific to conflict situations, raising the possibility that the negative valence of such interactions may explain why negative communication exhibited more robust links with relationship satisfaction than positive communication. Nguyen et al.’s observed communication measure was coded on the basis of one problem-solving task and two support tasks. Given that prior research demonstrated that positive communication exerts more influence than negative communication in the context of positively valenced interactions, such as when partners share positive events with one another (Gable et al., 2006), it is possible that the inclusion of supportive interactions in the assessment of communication accounts for the more robust positive communication/satisfaction association (and lack of a main negative communication/relationship satisfaction effect for men). It is important for future research to continue exploring within-person communication-satisfaction associations in other contexts.

### Limitations, Future Directions, and Implications

The findings presented here must be interpreted in light of limitations across these studies. First, as just mentioned, our focus on conflict and problem-solving communication leaves open the possibility that broader communication patterns or communication in other contexts would have different associations with satisfaction. Second, Studies 1 and 3 did not have optimal measurement. Study 1 did not assess positive communication and Study 3 relied on shortened measures of all constructs. These studies drew from large national samples of couples, as well, necessitating the use of self-reported assessment. The inclusion of Study 2 with robust measurement (observed and self-reported) from a less diverse sample was particularly valuable in this regard. Third, the time lags in each study (4 months in Study 1 and 1 year in Studies 2 and 3) cannot address the possibility that there may be more consistent short-term associations between couple communication and relationship satisfaction.^1^ Along these lines, all self-reports of communication and relationship satisfaction were assessed with trait-like wording, asking participants to report how they typically communicate during conflict situations and their overall relationship satisfaction. State-like measurement focused on communication and satisfaction at a specific point in time (as was the case with the observational measurement in Study 2) coupled with daily or weekly data collection protocols would be valuable to determine whether linkages between communication and satisfaction might exist over shorter lags than used here.

With these limitations in mind, the results from this research have theoretical and empirical implications. Although couple communication has long been theorized as a central predictor of relationship satisfaction (Bandura, 1977; Gottman, 1979; Markman, 1979; Wills et al., 1974), scholars have questioned the robustness of the communication to relationship satisfaction pathway (Johnson & Bradbury, 2015; Karney & Bradbury, 2020; Lavner et al., 2016). Findings from this study provide novel longitudinal within-person evidence that underscore such concerns; even though some significant cross-lagged links were evident in the analyses, within-couple deviations in communication were not as robustly associated with future relationship satisfaction as would be anticipated based on the behavioral models of intimate relations. This study did reveal, however, robust concurrent within-person links between negative communication and relationship satisfaction: Men’s and women’s relationship satisfaction was higher than average at times when negative communication was lower than average.

These results suggest that the linkage between negative communication and satisfaction may be better conceptualized as one consisting predominately of covariation rather than prediction of change. Such a conclusion aligns with findings from a large machine learning study that examined a wide swath of individual and relational (including conflict) between-person predictors of relationship satisfaction in 43 longitudinal couple studies (Joel et al., 2020). Cross-sectionally, these analyses accounted for up to 46% of the variation in relationship satisfaction, but the longitudinal prediction of future changes in satisfaction proved elusive: Only 5% of the variance in change in satisfaction was predicted in any analysis. Together, these findings suggest that relationship science has been more successful in understanding what predicts how satisfied people feel with their relationship in the moment than in the future.

Given that all relationship-relevant processes play out at some level via verbal or nonverbal communication (Heyman, 2001), it is critical for the field to better articulate exactly how communication plays a role in couples’ relationship satisfaction. We offer three suggestions in this regard. First, an increased focus on moderators of the within-person longitudinal linkages between communication and relationship satisfaction could articulate the conditions under which a deviation in a given couple’s communication patterns is likely to be more or less impactful on their relationship satisfaction. Much valuable research in this vein has focused on additional dimensions of couple communication, such as the directness of the communication (Overall et al., 2009) or severity of the problems being discussed (McNulty & Russell, 2010), that moderate the impact of conflict communication on satisfaction. Another possibility would be to focus on how intrapersonal explanations of partner communication behaviors (e.g., attributions) may buffer the impact of communication on relationship satisfaction (Bradbury & Fincham, 1990). Moderators outside of the couple relationship deserve attention as well, such as contextual influences and individual characteristics. For example, as mentioned, Nguyen et al.’s (2020) study found that within-person fluctuations in negative communication for men (and effective communication for both men and women) were associated with relationship satisfaction deviations only at times when they experienced higher than average stress levels. Continued research in these areas would be of considerable benefit to further refine understanding of the conditions in which couple communication is likely to be more consequential for relationship satisfaction.

Second, the robust concurrent within-person associations between negative communication and relationship satisfaction suggest that the focus on longitudinal associations between communication and satisfaction may be misplaced. Rather, reductions in negative communication may be a concurrent facet of increased relationship satisfaction, or conversely, increased relationship satisfaction may reflect reductions in negative communication. A shift toward emphasizing covariation raises interesting questions for future research about how these variables come to be linked, including whether couples do in fact form judgments about their relationship on the basis of their communication in the manner long suggested by behavioral theory (Bandura, 1977; Gottman, 1979; Karney & Bradbury, 1995; Koerner & Jacobson, 1994; Markman, 1979; Wills et al., 1974) or whether this covariation simply reflects a more global level of functioning such that at times couples are functioning well, they function well in multiple domains and at times they are functioning poorly, they function poorly in multiple domains.

Third, these findings suggest that theories of relationship development would benefit from increased attention to interpersonal factors other than problem-solving communication behaviors that might prove to be more robust predictors of change in relationship satisfaction over time. Along these lines, prior research has found links between positively valenced communication in nonconflict situations and relationship satisfaction. For example, recent work has demonstrated that within-person increases in perceived expressions of gratitude from one’s partner predicted an intraindividual reduction in future anxious attachment (Park et al., 2019). Insofar as reductions in anxious attachment might serve to increase relationship satisfaction, expressions of gratitude might be one interpersonal process that predicts changes in relationship satisfaction. Alternatively, prior research found that partner responses to positive events were a more robust between-person predictor of future relationship satisfaction than responses to negative events (Gable et al., 2006) and a large literature has found between-person associations between the provision of support during stressful circumstances and relationship satisfaction (Bodenmann, 2005). Future within-person examination of these and other nonconflict communication processes may prove useful to refine perspectives specifying how communication influences the development of relationship satisfaction.

### Conclusion

This multistudy article aimed to understand whether within-couple changes in communication predicted future within-couple changes in relationship satisfaction across 4-month and 1-year intervals. Sophisticated analysis of three high-quality longitudinal studies of couple relations revealed no consistent cross-study lagged within-person linkages between communication and satisfaction despite a smattering of lagged within-person negative communication/relationship satisfaction associations. These findings, viewed in light of the consistent within-person evidence of concurrent linkages between satisfaction and negative communication, dovetail with other recent findings indicating that the causal influence of communication on satisfaction may be limited (e.g., Lavner et al., 2016), and suggest that communication behavior may be a correlate of satisfaction rather than a cause. Such findings should prove useful to informing theoretical perspectives on the development of couple functioning that emphasize communication as a key factor predicting changes in relationship satisfaction over time toward perspectives that seek to better understand the covariation between satisfaction and communication.

## Supplemental Material

sj-docx-1-psp-10.1177_01461672211016920 – Supplemental material for Within-Couple Associations Between Communication and Relationship Satisfaction Over Time Supplemental material, sj-docx-1-psp-10.1177_01461672211016920 for Within-Couple Associations Between Communication and Relationship Satisfaction Over Time by Matthew D. Johnson, Justin A. Lavner, Marcus Mund, Martina Zemp, Scott M. Stanley, Franz J. Neyer, Emily A. Impett, Galena K. Rhoades, Guy Bodenmann, Rebekka Weidmann, Janina Larissa Bühler, Robert Philip Burriss, Jenna Wünsche and Alexander Grob in Personality and Social Psychology Bulletin

## Notes

1 We obtained daily diary data from a fourth study to test this possibility. Unfortunately, these models failed to converge. A full method section and detailed description of these analyses are provided in the Supplemental Materials.
