# Supplementary material for: Within-Couple Associations Between Communication and Relationship Satisfaction Over Time
Source: Pers Soc Psychol Bull. 2021 May 24;48(4):534–49. doi: 10.1177/01461672211016920 (PMC8915221; doi:10.1177/01461672211016920)
Supplement: sj-docx-1-psp-10.1177_01461672211016920 – Supplemental material for Within-Couple Associations Between Communication and Relationship Satisfaction Over Time [file sj-docx-1-psp-10.1177_01461672211016920.docx]

**Table of Contents**

1. Correlation Tables Page 2
   1. Study 1 (RDS): Supplementary Table 1
   2. Study 2 (PASEZ): Supplementary Tables 2 – 5
   3. Study 3 (Pairfam): Supplementary Tables 6 – 7
2. Detailed Analytic Plan Page 10
3. Tests of Measurement Invariance Page 15
4. Full ALT-SR Model Results Page 20
   1. Study 1 (RDS): Supplementary Table 10
   2. Study 2 (PASEZ): Supplementary Tables 11 – 14
   3. Study 3 (Pairfam): Supplementary Tables 15 – 16
5. Between-Person Associations Page 28
   1. Introduction
   2. Results for Study 1 (RDS)
   3. Results for Study 2 (PASEZ)
   4. Results for Study 3 (Pairfam)
6. Dyadic ALT-SR Models Page 37
   1. Introduction
   2. Results for Study 1 (RDS)
   3. Results for Study 2 (PASEZ)
   4. Results for Study 3 (Pairfam)
7. Overview of Study 4 Page 64
8. References Page 73

**1. Correlation Tables**

Supplementary Table 1

*Correlations Among Negative Communication and Relationship Satisfaction for Male and Female Partners in the RDS Study (Study 1; n = 316 couples)*

| Variable | Wave | 1 | 2 | 3 | 4 | 5 | 6 | 7 | 8 | 9 | 10 | 11 | 12 | 13 | 14 | 15 | 16 | 17 | 18 | 19 | 20 | 21 |
| --- | --- | --- | --- | --- | --- | --- | --- | --- | --- | --- | --- | --- | --- | --- | --- | --- | --- | --- | --- | --- | --- | --- |
| 1. Male Negative | 1 | — |  |  |  |  |  |  |  |  |  |  |  |  |  |  |  |  |  |  |  |  |
| 2. Male Negative | 2 | .64* | — |  |  |  |  |  |  |  |  |  |  |  |  |  |  |  |  |  |  |  |
| 3. Male Negative | 3 | .58* | .71* | — |  |  |  |  |  |  |  |  |  |  |  |  |  |  |  |  |  |  |
| 4. Male Negative | 4 | .57* | .70* | .72* | — |  |  |  |  |  |  |  |  |  |  |  |  |  |  |  |  |  |
| 5. Male Negative | 5 | .55* | .65* | .66* | .71* | — |  |  |  |  |  |  |  |  |  |  |  |  |  |  |  |  |
| 6. Female Negative | 1 | .59* | .51* | .43* | .42* | .39* | — |  |  |  |  |  |  |  |  |  |  |  |  |  |  |  |
| 7. Female Negative | 2 | .47* | .56* | .50* | .50* | .48* | .64* | — |  |  |  |  |  |  |  |  |  |  |  |  |  |  |
| 8. Female Negative | 3 | .39* | .49* | .53* | .50* | .50* | .60* | .75* | — |  |  |  |  |  |  |  |  |  |  |  |  |  |
| 9. Female Negative | 4 | .35* | .38* | .49* | .51* | .46* | .50* | .61* | .67* | — |  |  |  |  |  |  |  |  |  |  |  |  |
| 10. Female Negative | 5 | .35* | .48* | .48* | .50* | .54* | .56* | .69* | .74* | .73* | — |  |  |  |  |  |  |  |  |  |  |  |
| 11. Male Satisfaction | 1 | -.51* | -.38* | -.37* | -.40* | -.37* | -.38* | -.34* | -.32* | -.30* | -.21* | — |  |  |  |  |  |  |  |  |  |  |
| 12. Male Satisfaction | 2 | -.42* | -.55* | -.49* | -.45* | -.53* | -.33* | -.40* | -.39* | -.32* | -.26* | .66* | — |  |  |  |  |  |  |  |  |  |
| 13. Male Satisfaction | 3 | -.40* | -.43* | -.54* | -.41* | -.47* | -.32* | -.38* | -.39* | -.36* | -.25* | .60* | .77* | — |  |  |  |  |  |  |  |  |
| 14. Male Satisfaction | 4 | -.34* | -.36* | -.45* | -.52* | -.53* | -.28* | -.36* | -.36* | -.34* | -.26* | .51* | .68* | .78* | — |  |  |  |  |  |  |  |
| 15. Male Satisfaction | 5 | -.41* | -.49* | -.57* | -.56* | -.68* | -.33* | -.44* | -.47* | -.48* | -.44* | .58* | .74* | .71* | .80* | — |  |  |  |  |  |  |
| 16. Female Satisfaction | 1 | -.46* | -.36* | -.35* | -.37* | -.28* | -.61* | -.50* | -.38* | -.42* | -.33* | .48* | .45* | .46* | .34* | .38* | — |  |  |  |  |  |
| 17. Female Satisfaction | 2 | -.43* | -.51* | -.46* | -.41* | -.35* | -.56* | -.68* | -.52* | -.49* | -.49* | .46* | .60* | .52* | .46* | .51* | .70* | — |  |  |  |  |
| 18. Female Satisfaction | 3 | -.34* | -.40* | -.43* | -.38* | -.33* | -.51* | -.58* | -.67* | -.57* | -.50* | .46* | .52* | .58* | .46* | .49* | .65* | .71* | — |  |  |  |
| 19. Female Satisfaction | 4 | -.34* | -.35* | -.42* | -.38* | -.36* | -.45* | -.57* | -.56* | -.69* | -.55* | .44* | .54* | .48* | .44* | .53* | .63* | .76* | .80* | — |  |  |
| 20. Female Satisfaction | 5 | -.28* | -.44* | -.41* | -.41* | -.43* | -.43* | -.55* | -.61* | -.64* | -.62* | .37* | .54* | .52* | .50* | .65* | .52* | .67* | .70* | .82* | — |  |
| 21. Rel. Length | 1 | .24* | .17* | .18* | .07 | .08 | .23* | .10 | .12 | .09 | .03 | -.21* | -.18* | -.10 | -.10 | -.17* | -.25* | -.14* | -.13* | -.12 | -.08 | — |
| *Mean* |  | 1.64 | 1.65 | 1.61 | 1.60 | 1.62 | 1.60 | 1.57 | 1.59 | 1.57 | 1.55 | .61 | .76 | .78 | .79 | .77 | .81 | .81 | .81 | .82 | .81 | 3.14 |
| *SD* |  | .48 | .48 | .46 | .47 | .50 | .49 | .48 | .50 | .47 | .48 | .13 | .16 | .15 | .16 | .18 | .16 | .17 | .17 | .17 | .18 | 2.86 |

*Notes*. Male = Male partner. Female = Female partner. Negative = Negative communication. Rel. = Relationship. Range is 0 – 3 for negative communication and 0 – 1 for relationship satisfaction. ^*^ *p* < .05.

Supplementary Table 2

*Correlations Among Observed Positive Communication and Relationship Satisfaction for Male and Female Partners in the PASEZ Study (Study 2; n = 365 couples)*

| Variable | Wave | 1 | 2 | 3 | 4 | 5 | 6 | 7 | 8 | 9 | 10 | 11 | 12 | 13 | 14 | 15 | 16 | 17 | 18 | 19 | 20 | 21 |
| --- | --- | --- | --- | --- | --- | --- | --- | --- | --- | --- | --- | --- | --- | --- | --- | --- | --- | --- | --- | --- | --- | --- |
| 1. Male Positive | 1 | — |  |  |  |  |  |  |  |  |  |  |  |  |  |  |  |  |  |  |  |  |
| 2. Male Positive | 2 | .44* | — |  |  |  |  |  |  |  |  |  |  |  |  |  |  |  |  |  |  |  |
| 3. Male Positive | 3 | .43* | .47* | — |  |  |  |  |  |  |  |  |  |  |  |  |  |  |  |  |  |  |
| 4. Male Positive | 4 | .42* | .44* | .43* | — |  |  |  |  |  |  |  |  |  |  |  |  |  |  |  |  |  |
| 5. Male Positive | 5 | .44* | .40* | .48* | .39* | — |  |  |  |  |  |  |  |  |  |  |  |  |  |  |  |  |
| 6. Female Positive | 1 | .33* | .19* | .20* | .11 | .18* | — |  |  |  |  |  |  |  |  |  |  |  |  |  |  |  |
| 7. Female Positive | 2 | .22* | .34* | .17* | .27* | .26* | .42* | — |  |  |  |  |  |  |  |  |  |  |  |  |  |  |
| 8. Female Positive | 3 | .24* | .20* | .35* | .27* | .33* | .35* | .39* | — |  |  |  |  |  |  |  |  |  |  |  |  |  |
| 9. Female Positive | 4 | .13 | .18* | .18* | .37* | .20* | .16* | .33* | .33* | — |  |  |  |  |  |  |  |  |  |  |  |  |
| 10. Female Positive | 5 | .12 | .07 | .22* | .27* | .35* | .19* | .24* | .29* | .37* | — |  |  |  |  |  |  |  |  |  |  |  |
| 11. Male Satisfaction | 1 | .11* | .10 | .02 | -.05 | .08 | .16* | .06 | -.11 | -.04 | -.01 | — |  |  |  |  |  |  |  |  |  |  |
| 12. Male Satisfaction | 2 | .04 | .10 | .01 | .03 | .07 | .11 | .11 | -.03 | .11 | -.04 | .75* | — |  |  |  |  |  |  |  |  |  |
| 13. Male Satisfaction | 3 | .11 | .19* | .11 | .05 | .13 | .08 | .10 | -.02 | .08 | .05 | .74* | .72* | — |  |  |  |  |  |  |  |  |
| 14. Male Satisfaction | 4 | .04 | .13 | .10 | .07 | .11 | .10 | .18* | .02 | .10 | .01 | .69* | .66* | .75* | — |  |  |  |  |  |  |  |
| 15. Male Satisfaction | 5 | .01 | .14* | .17* | .05 | .17* | .13 | .19* | .08 | .13 | .05 | .61* | .59* | .70* | .74* | — |  |  |  |  |  |  |
| 16. Female Satisfaction | 1 | .16* | .19* | .14* | .07 | .17* | .18* | .05 | .10 | .10 | .05 | .56* | .49* | .50* | .44* | .48* | — |  |  |  |  |  |
| 17. Female Satisfaction | 2 | .07 | .09 | .13* | .08 | .14* | .09 | .02 | .11 | .15* | .05 | .45* | .58* | .49* | .48* | .47* | .68* | — |  |  |  |  |
| 18. Female Satisfaction | 3 | .12 | .12 | .10 | .05 | .14 | .13* | .07 | .07 | .08 | .06 | .44* | .40* | .58* | .46* | .50* | .67* | .67* | — |  |  |  |
| 19. Female Satisfaction | 4 | .15* | .17* | .19* | .15* | .17* | .12 | .15* | .17* | .10 | .02 | .43* | .45* | .48* | .60* | .52* | .65* | .67* | .67* | — |  |  |
| 20. Female Satisfaction | 5 | .15* | .18* | .14* | .10 | .17* | .14* | .16* | .15* | .13 | .03 | .33* | .32* | .39* | .46* | .59* | .63* | .66* | .64* | .73* | — |  |
| 21. Rel. Length | 1 | -.22* | -.22* | -.14* | -.08 | -.14 | -.10 | -.17* | -.07 | -.13 | -.07 | .01 | -.02 | .03 | .07 | .09 | -.08 | -.05 | -.07 | -.07 | -.02 | — |
| *Mean* |  | 16.66 | 9.73 | 10.25 | 13.83 | 14.12 | 15.97 | 8.93 | 8.67 | 9.86 | 8.53 | 5.18 | 5.19 | 5.18 | 5.16 | 5.14 | 5.22 | 5.13 | 5.15 | 5.08 | 5.06 | 21.06 |
| *SD* |  | 11.09 | 8.61 | 8.31 | 11.15 | 10.98 | 10.46 | 7.28 | 7.03 | 8.18 | 7.43 | .73 | .72 | .77 | .73 | .76 | .69 | .79 | .78 | .87 | .91 | 18.03 |

*Notes*. Male = Male partner. Female = Female partner. Positive = Positive communication. Rel. = Relationship. Range is 0 – 60 for positive communication and 1 – 6 for relationship satisfaction.

^*^ *p* < .05.

Supplementary Table 3

*Correlations Among Observed Negative Communication and Relationship Satisfaction for Male and Female Partners in the PASEZ Study (Study 2; n = 365 couples)*

| Variable | Wave | 1 | 2 | 3 | 4 | 5 | 6 | 7 | 8 | 9 | 10 | 11 | 12 | 13 | 14 | 15 | 16 | 17 | 18 | 19 | 20 | 21 |
| --- | --- | --- | --- | --- | --- | --- | --- | --- | --- | --- | --- | --- | --- | --- | --- | --- | --- | --- | --- | --- | --- | --- |
| 1. Male Negative | 1 | — |  |  |  |  |  |  |  |  |  |  |  |  |  |  |  |  |  |  |  |  |
| 2. Male Negative | 2 | .34* | — |  |  |  |  |  |  |  |  |  |  |  |  |  |  |  |  |  |  |  |
| 3. Male Negative | 3 | .32* | .59* | — |  |  |  |  |  |  |  |  |  |  |  |  |  |  |  |  |  |  |
| 4. Male Negative | 4 | .32* | .56* | .46* | — |  |  |  |  |  |  |  |  |  |  |  |  |  |  |  |  |  |
| 5. Male Negative | 5 | .45* | .42* | .47* | .49* | — |  |  |  |  |  |  |  |  |  |  |  |  |  |  |  |  |
| 6. Female Negative | 1 | .49* | .33* | .28* | .28* | .21* | — |  |  |  |  |  |  |  |  |  |  |  |  |  |  |  |
| 7. Female Negative | 2 | .20* | .59* | .36* | .37* | .30* | .35* | — |  |  |  |  |  |  |  |  |  |  |  |  |  |  |
| 8. Female Negative | 3 | .17* | .46* | .66* | .43* | .25* | .38* | .54* | — |  |  |  |  |  |  |  |  |  |  |  |  |  |
| 9. Female Negative | 4 | .20* | .40* | .34* | .63* | .27* | .42* | .52* | .60* | — |  |  |  |  |  |  |  |  |  |  |  |  |
| 10. Female Negative | 5 | .26* | .32* | .30* | .36* | .69* | .31* | .39* | .37* | .42* | — |  |  |  |  |  |  |  |  |  |  |  |
| 11. Male Satisfaction | 1 | -.18* | -.11 | -.02 | .00 | -.03 | -.20* | -.19* | -.11 | -.17* | -.13 | — |  |  |  |  |  |  |  |  |  |  |
| 12. Male Satisfaction | 2 | -.10 | -.12* | -.01 | -.06 | -.02 | -.13* | -.24* | -.13* | -.22* | -.10 | .75* | — |  |  |  |  |  |  |  |  |  |
| 13. Male Satisfaction | 3 | -.06 | -.17* | -.09 | -.01 | -.02 | -.13* | -.17* | -.23* | -.25* | -.15* | .74* | .72* | — |  |  |  |  |  |  |  |  |
| 14. Male Satisfaction | 4 | -.06 | -.18* | -.12 | -.12 | -.10 | -.18* | -.25* | -.30* | -.35* | -.20* | .69* | .66* | .75* | — |  |  |  |  |  |  |  |
| 15. Male Satisfaction | 5 | -.02 | -.17* | -.11 | .00 | -.09 | -.20* | -.22* | -.27* | -.27* | -.28 | .61* | .59* | .70* | .74* | — |  |  |  |  |  |  |
| 16. Female Satisfaction | 1 | -.29* | -.22* | -.19* | -.16* | -.17* | -.30* | -.26* | -.24* | -.31* | -.24* | .56* | .49* | .50* | .44* | .48* | — |  |  |  |  |  |
| 17. Female Satisfaction | 2 | -.20* | -.17* | -.16* | -.09 | -.12 | -.25* | -.29* | -.23* | -.24* | -.17* | .45* | .58* | .49* | .48* | .47* | .68* | — |  |  |  |  |
| 18. Female Satisfaction | 3 | -.10 | -.15* | -.16* | -.07 | -.11 | -.17* | -.20* | -.22* | -.27* | -.18* | .44* | .40* | .58* | .46* | .50* | .67* | .67* | — |  |  |  |
| 19. Female Satisfaction | 4 | -.16* | -.25* | -.18* | -.15* | -.14* | -.26* | -.35* | -.32* | -.39* | -.28* | .43* | .45* | .48* | .60* | .52* | .65* | .67* | .67* | — |  |  |
| 20. Female Satisfaction | 5 | -.15* | -.25* | -.30* | -.13 | -.23* | -.21* | -.27* | -.33* | -.31* | -.37* | .33* | .32* | .39* | .46* | .59* | .63* | .66* | .64* | .73* | — |  |
| 21. Rel. Length | 1 | .07 | .02 | -.06 | .01 | .08 | -.08 | -.04 | -.10 | .02 | .07 | .01 | -.02 | .03 | .07 | .09 | -.08 | -.05 | -.07 | -.07 | -.02 | — |
| *Mean* |  | 5.85 | 8.49 | 9.53 | 12.25 | 11.60 | 5.92 | 5.72 | 7.99 | 7.88 | 8.65 | 5.18 | 5.19 | 5.18 | 5.16 | 5.14 | 5.22 | 5.13 | 5.15 | 5.08 | 5.06 | 21.06 |
| *SD* |  | 8.61 | 10.18 | 11.42 | 12.67 | 13.03 | 7.42 | 7.06 | 9.70 | 9.72 | 9.56 | .73 | .72 | .77 | .73 | .76 | .69 | .79 | .78 | .87 | .91 | 18.03 |

*Notes*. Male = Male partner. Female = Female partner. Positive = Positive communication. Rel. = Relationship. Range is 0 – 89 for negative communication and 1 – 6 for relationship satisfaction.

^*^ *p* < .05.

Supplementary Table 4

*Correlations Among Self-Reported Positive Communication and Relationship Satisfaction for Male and Female Partners in the PASEZ Study (Study 2; n = 365 couples)*

| Variable | Wave | 1 | 2 | 3 | 4 | 5 | 6 | 7 | 8 | 9 | 10 | 11 | 12 | 13 | 14 | 15 | 16 | 17 | 18 | 19 | 20 | 21 |
| --- | --- | --- | --- | --- | --- | --- | --- | --- | --- | --- | --- | --- | --- | --- | --- | --- | --- | --- | --- | --- | --- | --- |
| 1. Male Positive | 1 | — |  |  |  |  |  |  |  |  |  |  |  |  |  |  |  |  |  |  |  |  |
| 2. Male Positive | 2 | .71* | — |  |  |  |  |  |  |  |  |  |  |  |  |  |  |  |  |  |  |  |
| 3. Male Positive | 3 | .69* | .73* | — |  |  |  |  |  |  |  |  |  |  |  |  |  |  |  |  |  |  |
| 4. Male Positive | 4 | .62* | .69* | .70* | — |  |  |  |  |  |  |  |  |  |  |  |  |  |  |  |  |  |
| 5. Male Positive | 5 | .64* | .74* | .73* | .70* | — |  |  |  |  |  |  |  |  |  |  |  |  |  |  |  |  |
| 6. Female Positive | 1 | .20* | .17* | .13* | .20* | .14* | — |  |  |  |  |  |  |  |  |  |  |  |  |  |  |  |
| 7. Female Positive | 2 | .24* | .19* | .17* | .22* | .17* | .63* | — |  |  |  |  |  |  |  |  |  |  |  |  |  |  |
| 8. Female Positive | 3 | .23* | .18* | .19* | .27* | .14* | .66* | .74* | — |  |  |  |  |  |  |  |  |  |  |  |  |  |
| 9. Female Positive | 4 | .19* | .20* | .22* | .28* | .16* | .58* | .61* | .68* | — |  |  |  |  |  |  |  |  |  |  |  |  |
| 10. Female Positive | 5 | .25* | .20* | .24* | .28* | .23* | .56* | .70* | .73* | .69* | — |  |  |  |  |  |  |  |  |  |  |  |
| 11. Male Satisfaction | 1 | .35* | .33* | .30* | .29* | .29* | .13* | .16* | .17* | .17* | .28* | — |  |  |  |  |  |  |  |  |  |  |
| 12. Male Satisfaction | 2 | .35* | .34* | .25* | .25* | .26* | .06 | .13* | .17* | .16* | .27* | .75* | — |  |  |  |  |  |  |  |  |  |
| 13. Male Satisfaction | 3 | .38* | .34* | .38* | .36* | .30* | .08 | .20* | .18* | .18* | .29* | .74* | .72* | — |  |  |  |  |  |  |  |  |
| 14. Male Satisfaction | 4 | .24* | .31* | .30* | .31* | .31* | .03 | .18* | .14* | .19* | .27* | .69* | .66* | .75* | — |  |  |  |  |  |  |  |
| 15. Male Satisfaction | 5 | .31* | .37* | .36* | .31* | .32* | .16* | .22* | .18* | .23* | .28* | .61* | .59* | .70* | .74* | — |  |  |  |  |  |  |
| 16. Female Satisfaction | 1 | .28* | .34* | .22* | .23* | .20* | .22* | .23* | .28* | .33* | .33* | .56* | .49* | .50* | .44* | .48* | — |  |  |  |  |  |
| 17. Female Satisfaction | 2 | .26* | .31* | .18* | .24* | .19* | .19* | .30* | .29* | .35* | .34* | .45* | .58* | .49* | .48* | .47* | .68* | — |  |  |  |  |
| 18. Female Satisfaction | 3 | .27* | .30* | .24* | .25* | .18* | .24* | .25* | .25* | .34* | .32* | .44* | .40* | .58* | .46* | .50* | .67* | .67* | — |  |  |  |
| 19. Female Satisfaction | 4 | .17* | .30* | .22* | .22* | .25* | .12 | .28* | .24* | .34* | .39* | .43* | .45* | .48* | .60* | .52* | .65* | .67* | .67* | — |  |  |
| 20. Female Satisfaction | 5 | .19* | .27* | .25* | .22* | .24* | .26* | .29* | .24* | .36* | .37* | .33* | .32* | .39* | .46* | .59* | .63* | .66* | .64* | .73* | — |  |
| 21. Rel. Length | 1 | -.14* | -.10 | -.05 | -.01 | .01 | -.15* | -.10 | -.10 | -.12 | -.06 | .01 | -.02 | .03 | .07 | .09 | -.08 | -.05 | -.07 | -.07 | -.02 | — |
| *Mean* |  | 4.05 | 4.06 | 4.03 | 4.05 | 4.03 | 4.27 | 4.31 | 4.26 | 4.08 | 4.15 | 5.18 | 5.19 | 5.18 | 5.16 | 5.14 | 5.22 | 5.13 | 5.15 | 5.08 | 5.06 | 21.06 |
| *SD* |  | .80 | .80 | .79 | .76 | .84 | .77 | .82 | .80 | .81 | .81 | .73 | .72 | .77 | .73 | .76 | .69 | .79 | .78 | .87 | .91 | 18.03 |

*Notes*. Male = Male partner. Female = Female partner. Positive = Positive communication. Rel. = Relationship. Range is 1 – 6 for positive communication and for relationship satisfaction.

^*^ *p* < .05.

Supplementary Table 5

*Correlations Among Self-Reported Negative Communication and Relationship Satisfaction for Male and Female Partners in the PASEZ Study (Study 2; n = 365 couples)*

| Variable | Wave | 1 | 2 | 3 | 4 | 5 | 6 | 7 | 8 | 9 | 10 | 11 | 12 | 13 | 14 | 15 | 16 | 17 | 18 | 19 | 20 | 21 |
| --- | --- | --- | --- | --- | --- | --- | --- | --- | --- | --- | --- | --- | --- | --- | --- | --- | --- | --- | --- | --- | --- | --- |
| 1. Male Negative | 1 | — |  |  |  |  |  |  |  |  |  |  |  |  |  |  |  |  |  |  |  |  |
| 2. Male Negative | 2 | .76* | — |  |  |  |  |  |  |  |  |  |  |  |  |  |  |  |  |  |  |  |
| 3. Male Negative | 3 | .72* | .78* | — |  |  |  |  |  |  |  |  |  |  |  |  |  |  |  |  |  |  |
| 4. Male Negative | 4 | .71* | .79* | .78* | — |  |  |  |  |  |  |  |  |  |  |  |  |  |  |  |  |  |
| 5. Male Negative | 5 | .62* | .74* | .76* | .80* | — |  |  |  |  |  |  |  |  |  |  |  |  |  |  |  |  |
| 6. Female Negative | 1 | .38* | .40* | .47* | .40* | .41* | — |  |  |  |  |  |  |  |  |  |  |  |  |  |  |  |
| 7. Female Negative | 2 | .49* | .49* | .49* | .42* | .44* | .79* | — |  |  |  |  |  |  |  |  |  |  |  |  |  |  |
| 8. Female Negative | 3 | .44* | .48* | .52* | .47* | .47* | .76* | .80* | — |  |  |  |  |  |  |  |  |  |  |  |  |  |
| 9. Female Negative | 4 | .40* | .41* | .48* | .42* | .48* | .70* | .71* | .74* | — |  |  |  |  |  |  |  |  |  |  |  |  |
| 10. Female Negative | 5 | .38* | .41* | .45* | .42* | .45* | .69* | .76* | .78* | .80* | — |  |  |  |  |  |  |  |  |  |  |  |
| 11. Male Satisfaction | 1 | -.44* | -.35* | -.31* | -.31* | -.22* | -.26* | -.23* | -.15* | -.13 | -.14* | — |  |  |  |  |  |  |  |  |  |  |
| 12. Male Satisfaction | 2 | -.41* | -.43* | -.33* | -.28* | -.26* | -.16* | -.27* | -.15* | -.17* | -.18* | .75* | — |  |  |  |  |  |  |  |  |  |
| 13. Male Satisfaction | 3 | -.37* | -.39* | -.37* | -.43* | -.33* | -.29* | -.26* | -.24* | -.25* | -.24* | .74* | .72* | — |  |  |  |  |  |  |  |  |
| 14. Male Satisfaction | 4 | -.34* | -.37* | -.37* | -.38* | -.38* | -.30* | -.32* | -.31* | -.36* | -.35* | .69* | .66* | .75* | — |  |  |  |  |  |  |  |
| 15. Male Satisfaction | 5 | -.34* | -.37* | -.35* | -.39* | -.37* | -.27* | -.29* | -.31* | -.37* | -.33* | .61* | .59* | .70* | .74* | — |  |  |  |  |  |  |
| 16. Female Satisfaction | 1 | -.35* | -.35* | -.29* | -.32* | -.25* | -.38* | -.34* | -.27* | -.29* | -.31* | .56* | .49* | .50* | .44* | .48* | — |  |  |  |  |  |
| 17. Female Satisfaction | 2 | -.39* | -.39* | -.28* | -.23* | -.22* | -.26* | -.38* | -.23* | -.30* | -.28* | .45* | .58* | .49* | .48* | .47* | .68* | — |  |  |  |  |
| 18. Female Satisfaction | 3 | -.33* | -.36* | -.33* | -.33* | -.26* | -.27* | -.27* | -.30* | -.28* | -.26* | .44* | .40* | .58* | .46* | .50* | .67* | .67* | — |  |  |  |
| 19. Female Satisfaction | 4 | -.28* | -.29* | -.31* | -.29* | -.25* | -.30* | -.29* | -.27* | -.42* | -.34* | .43* | .45* | .48* | .60* | .52* | .65* | .67* | .67* | — |  |  |
| 20. Female Satisfaction | 5 | -.30* | -.30* | -.26* | -.28* | -.23* | -.29* | -.29* | -.28* | -.38* | -.35* | .33* | .32* | .39* | .46* | .59* | .63* | .66* | .64* | .73* | — |  |
| 21. Rel. Length | 1 | -.09 | -.08 | -.14* | -.19* | -.21* | -.20* | -.18* | -.22* | -.21* | -.21* | .01 | -.02 | .03 | .07 | .09 | -.08 | -.05 | -.07 | -.07 | -.02 | — |
| *Mean* |  | 2.00 | 1.97 | 1.97 | 1.86 | 1.82 | 2.24 | 2.16 | 2.14 | 2.04 | 2.00 | 5.18 | 5.19 | 5.18 | 5.16 | 5.14 | 5.22 | 5.13 | 5.15 | 5.08 | 5.06 | 21.06 |
| *SD* |  | .56 | .52 | .55 | .49 | .49 | .63 | .58 | .59 | .54 | .56 | .73 | .72 | .77 | .73 | .76 | .69 | .79 | .78 | .87 | .91 | 18.03 |

*Notes*. Male = Male partner. Female = Female partner. Positive = Positive communication. Rel. = Relationship. Range is 1 – 6 for negative communication and for relationship satisfaction.

^*^ *p* < .05.

Supplementary Table 6

*Correlations Among Positive Communication and Relationship Satisfaction for Male and Female Partners in the Pairfam Study (Study 3; n = 3,405 couples)*

| Variable | Wave | 1 | 2 | 3 | 4 | 5 | 6 | 7 | 8 | 9 | 10 | 11 | 12 | 13 | 14 | 15 | 16 | 17 | 18 | 19 | 20 | 21 |
| --- | --- | --- | --- | --- | --- | --- | --- | --- | --- | --- | --- | --- | --- | --- | --- | --- | --- | --- | --- | --- | --- | --- |
| 1. Male Positive | 1 | — |  |  |  |  |  |  |  |  |  |  |  |  |  |  |  |  |  |  |  |  |
| 2. Male Positive | 2 | .35* | — |  |  |  |  |  |  |  |  |  |  |  |  |  |  |  |  |  |  |  |
| 3. Male Positive | 3 | .35* | .41* | — |  |  |  |  |  |  |  |  |  |  |  |  |  |  |  |  |  |  |
| 4. Male Positive | 4 | .30* | .39* | .40* | — |  |  |  |  |  |  |  |  |  |  |  |  |  |  |  |  |  |
| 5. Male Positive | 5 | .32* | .35* | .42* | .42* | — |  |  |  |  |  |  |  |  |  |  |  |  |  |  |  |  |
| 6. Female Positive | 1 | .08* | .08* | .07* | .06* | .06* | — |  |  |  |  |  |  |  |  |  |  |  |  |  |  |  |
| 7. Female Positive | 2 | .09* | .11* | .10* | .09* | .07* | .37* | — |  |  |  |  |  |  |  |  |  |  |  |  |  |  |
| 8. Female Positive | 3 | .09* | .07* | .10* | .09* | .13* | .43* | .45* | — |  |  |  |  |  |  |  |  |  |  |  |  |  |
| 9. Female Positive | 4 | .10* | .13* | .12* | .12* | .15* | .35* | .39* | .45* | — |  |  |  |  |  |  |  |  |  |  |  |  |
| 10. Female Positive | 5 | .09* | .13* | .10* | .12* | .13* | .35* | .38* | .45* | .46* | — |  |  |  |  |  |  |  |  |  |  |  |
| 11. Male Satisfaction | 1 | .14* | .12* | .09* | .11* | .11* | .08* | .09* | .11* | .15* | .11* | — |  |  |  |  |  |  |  |  |  |  |
| 12. Male Satisfaction | 2 | .10* | .19* | .10* | .12* | .06* | .04* | .11* | .09* | .09* | .07* | .42* | — |  |  |  |  |  |  |  |  |  |
| 13. Male Satisfaction | 3 | .13* | .17* | .15* | .13* | .15* | .09* | .13* | .12* | .12* | .11* | .32* | .47* | — |  |  |  |  |  |  |  |  |
| 14. Male Satisfaction | 4 | .08* | .14* | .11* | .15* | .14* | .09* | .07* | .08* | .07* | .04 | .28* | .37* | .40* | — |  |  |  |  |  |  |  |
| 15. Male Satisfaction | 5 | .11* | .15* | .16* | .13* | .21* | .10* | .13* | .07* | .17* | .16* | .28* | .41* | .39* | .45* | — |  |  |  |  |  |  |
| 16. Female Satisfaction | 1 | .11* | .13* | .10* | .09* | .10* | .11* | .13* | .13* | .11* | .13* | .21* | .26* | .19* | .18* | .19* | — |  |  |  |  |  |
| 17. Female Satisfaction | 2 | .09* | .16* | .16* | .10* | .08* | .09* | .17* | .14* | .11* | .15* | .18* | .33* | .23* | .20* | .21* | .36* | — |  |  |  |  |
| 18. Female Satisfaction | 3 | .06* | .11* | .11* | .08* | .11* | .10* | .13* | .15* | .15* | .18* | .19* | .27* | .28* | .23* | .22* | .34* | .42* | — |  |  |  |
| 19. Female Satisfaction | 4 | .05 | .09* | .11* | .10* | .12* | .10* | .14* | .15* | .19* | .15* | .16* | .21* | .22* | .21* | .21* | .25* | .36* | .43* | — |  |  |
| 20. Female Satisfaction | 5 | .08* | .15* | .13* | .15* | .17* | .13* | .18* | .15* | .17* | .22* | .17* | .22* | .22* | .22* | .36* | .32* | .40* | .42* | .48* | — |  |
| 21. Rel. Length | 1 | -.09* | -.07* | -.05* | -.10* | -.07* | -.10* | -.08* | -.08* | -.08* | -.06* | -.01 | .02 | .03 | -.02 | .01 | -.02 | -.03 | -.02 | -.07* | -.02 | — |
| *Mean* |  | 3.65 | 3.65 | 3.55 | 3.61 | 3.51 | 3.88 | 3.90 | 3.80 | 3.84 | 3.75 | 8.33 | 8.12 | 8.02 | 7.86 | 7.89 | 8.26 | 8.01 | 7.95 | 7.80 | 7.85 | 8.79 |
| *SD* |  | .91 | .82 | .81 | .82 | .79 | .87 | .82 | .82 | .84 | .81 | 1.97 | 1.96 | 1.95 | 2.17 | 2.01 | 2.04 | 2.12 | 2.08 | 2.17 | 2.07 | 5.70 |

*Notes*. Male = Male partner. Female = Female partner. Positive = Positive communication. Rel. = Relationship. Range is 1 – 5 for positive communication and 0 – 10 for relationship satisfaction.

^*^*p* < .05.

Supplementary Table 7

*Correlations Among Negative Communication and Relationship Satisfaction for Male and Female Partners in the Pairfam Study (Study 3; n = 3,405 couples)*

| Variable | Wave | 1 | 2 | 3 | 4 | 5 | 6 | 7 | 8 | 9 | 10 | 11 | 12 | 13 | 14 | 15 | 16 | 17 | 18 | 19 | 20 | 21 |
| --- | --- | --- | --- | --- | --- | --- | --- | --- | --- | --- | --- | --- | --- | --- | --- | --- | --- | --- | --- | --- | --- | --- |
| 1. Male Negative | 1 | — |  |  |  |  |  |  |  |  |  |  |  |  |  |  |  |  |  |  |  |  |
| 2. Male Negative | 2 | .57* | — |  |  |  |  |  |  |  |  |  |  |  |  |  |  |  |  |  |  |  |
| 3. Male Negative | 3 | .58* | .62* | — |  |  |  |  |  |  |  |  |  |  |  |  |  |  |  |  |  |  |
| 4. Male Negative | 4 | .53* | .60* | .65* | — |  |  |  |  |  |  |  |  |  |  |  |  |  |  |  |  |  |
| 5. Male Negative | 5 | .50* | .56* | .59* | .61* | — |  |  |  |  |  |  |  |  |  |  |  |  |  |  |  |  |
| 6. Female Negative | 1 | .27* | .25* | .23* | .24* | .18* | — |  |  |  |  |  |  |  |  |  |  |  |  |  |  |  |
| 7. Female Negative | 2 | .24* | .31* | .26* | .28* | .23* | .63* | — |  |  |  |  |  |  |  |  |  |  |  |  |  |  |
| 8. Female Negative | 3 | .23* | .28* | .33* | .29* | .28* | .58* | .65* | — |  |  |  |  |  |  |  |  |  |  |  |  |  |
| 9. Female Negative | 4 | .25* | .27* | .31* | .34* | .29* | .53* | .63* | .69* | — |  |  |  |  |  |  |  |  |  |  |  |  |
| 10. Female Negative | 5 | .21* | .26* | .28* | .30* | .32* | .51* | .61* | .65* | .67* | — |  |  |  |  |  |  |  |  |  |  |  |
| 11. Male Satisfaction | 1 | -.27* | -.21* | -.19* | -.15* | -.13* | -.20* | -.15* | -.15* | -.16* | -.10* | — |  |  |  |  |  |  |  |  |  |  |
| 12. Male Satisfaction | 2 | -.26* | -.30* | -.22* | -.23* | -.21* | -.23* | -.25* | -.20* | -.19* | -.21* | .42* | — |  |  |  |  |  |  |  |  |  |
| 13. Male Satisfaction | 3 | -.21* | -.22* | -.29* | -.23* | -.18* | -.17* | -.15* | -.22* | -.17* | -.15* | .32* | .47* | — |  |  |  |  |  |  |  |  |
| 14. Male Satisfaction | 4 | -.17* | -.20* | -.21* | -.31* | -.22* | -.16* | -.11* | -.20* | -.19* | -.18* | .28* | .37* | .40* | — |  |  |  |  |  |  |  |
| 15. Male Satisfaction | 5 | -.19* | -.23* | -.23* | -.28* | -.35* | -.14* | -.17* | -.18* | -.21* | -.27* | .28* | .41* | .39* | .45* | — |  |  |  |  |  |  |
| 16. Female Satisfaction | 1 | -.22* | -.22* | -.16* | -.17* | -.14* | -.29* | -.26* | -.23* | -.20* | -.20* | .21* | .26* | .19* | .18* | .19* | — |  |  |  |  |  |
| 17. Female Satisfaction | 2 | -.19* | -.23* | -.21* | -.21* | -.18* | -.26* | -.34* | -.25* | -.25* | -.24* | .18* | .33* | .23* | .20* | .21* | .36* | — |  |  |  |  |
| 18. Female Satisfaction | 3 | -.16* | -.20* | -.26* | -.21* | -.23* | -.24* | -.26* | -.36* | -.30* | -.27* | .19* | .27* | .28* | .23* | .22* | .34* | .42* | — |  |  |  |
| 19. Female Satisfaction | 4 | -.19* | -.15* | -.21* | -.25* | -.22* | -.16* | -.20* | -.26* | -.34* | -.21* | .16* | .21* | .22* | .21* | .21* | .25* | .36* | .43* | — |  |  |
| 20. Female Satisfaction | 5 | -.17* | -.19* | -.22* | -.24* | -.29* | -.24* | -.30* | -.32* | -.35* | -.40* | .17* | .22* | .22* | .22* | .36* | .32* | .40* | .42* | .48* | — |  |
| 21. Rel. Length | 1 | .05* | .06* | -.02 | .06* | .02 | -.07* | -.07* | -.07* | -.04 | -.06* | -.01 | .02 | .03 | -.02 | .01 | -.02 | -.03 | -.02 | -.07* | -.02 | — |
| *Mean* |  | 2.03 | 2.05 | 2.00 | 1.96 | 1.99 | 2.16 | 2.10 | 2.09 | 2.06 | 2.09 | 8.33 | 8.12 | 8.02 | 7.86 | 7.89 | 8.26 | 8.01 | 7.95 | 7.80 | 7.85 | 8.79 |
| *SD* |  | .77 | .73 | .72 | .73 | .72 | .82 | .78 | .80 | .78 | .78 | 1.97 | 1.96 | 1.95 | 2.17 | 2.01 | 2.04 | 2.12 | 2.08 | 2.17 | 2.07 | 5.70 |

*Notes*. Male = Male partner. Female = Female partner. Negative = Negative communication. Rel. = Relationship. Range is 1 – 5 for negative communication and 0 – 10 for relationship satisfaction.

^*^*p* < .05.

**2. Detailed Analytic Plan**

The computation of our ALT-SR models proceeded as follows. First, we identified the best-fitting growth curve separately for each construct (relationship satisfaction and positive and negative communication for each partner) to produce unbiased within-person estimates (Voelkle, 2008). Intercept loadings in each model were set to 1, corresponding to initial levels at the outset of the study, and linear slope loadings signify the uniform passage of time because data collection occurred at equidistant intervals in each study (e.g., 0 [Wave 1], 1 [Wave 2], 2 [Wave 3], and so on). Nonlinear patterns were considered with latent basis growth models (McArdle & Epstein, 1987), which allow the slope loadings to be estimated by the data (first and last measurement occasion loadings are set at 0 and 1, respectively, to identify the model). The slope loadings were then fixed to the estimated values for model comparisons and subsequent analyses. Each construct was fitted to a series of increasingly complex growth models (e.g., fixed intercept, random intercept, fixed linear slope) and the change in model chi-square determined the best-fitting trajectory. Once we identified the shape of the trajectory, we added autoregressive paths to the construct residuals at each measurement occasion and tested the suitability of equality constraints on these paths with chi-square difference testing.

Next, the ALT-SR model depicted in Figure 1 was computed using the individual construct growth curves. We tested a series of bivariate models between each partner’s relationship satisfaction and their own communication (referred to as within-partner communication models), and each partner’s relationship satisfaction and their partner’s communication (referred to as cross-partner communication models). This resulted in four models for positive communication (i.e., female partner relationship satisfaction with her own positive communication, female partner relationship satisfaction with male partner positive communication, male partner relationship satisfaction with his own positive communication, and male partner relationship satisfaction with female partner positive communication) and four models for negative communication in each study.

Once the ALT-SR model was computed, we applied equality constraints across time to the paths linking the cross-construct residuals (e.g., the longitudinal cross-lagged paths and within-time cross-construct correlations) and tested with chi-square difference testing. In a final step, we added relationship duration as a between-person control variable and reported final estimates.

Missing data were handled with full-information maximum likelihood estimation (FIML), which computes model parameters with all available information in the variance/covariance matrix (Enders, 2011). Among couples in Study 1 (RDS; n = 316), 19.6% were lost to attrition and 24.4% terminated their relationship across the duration of the study. Female partners in couples who ended their relationship reported significantly lower relationship satisfaction (*M* = .73, *SD* = .20) than female partners in couples who remained together (*M* = .84, *SD* = .14, *t*(112.17) = 4.19, *p* < .001) or dropped out of the study (*M* = .83, *SD* = .14, *t*(137) = 3.29, *p* = .001). No differences were found for dissolving couples relative to attriting and continuing couples in regard to Wave 1 reports of male partner relationship satisfaction or for either partner’s negative communication and there were no differences between attriting and continuing couples (all *p*s > .003).

For the 368 couples in Study 2 (PASEZ), 47.9% remained partnered through the five years of this study, 41.1% were lost to attrition, and 11.0% ended their relationship. In the *t*-test comparisons, one pattern emerged: female partners in couples who ended their relationship had significantly higher self-reported positive communication at Wave 1 (*M* = 4.75, *SD* = .84) compared to female partners in couples who remained together through the duration of the study (*M* = 4.21, *SD* = .72, *t*(215) = 4.171, *p* < .001) and to female partners who attrited (*M* = 4.21, *SD* = .78, *t*(188) = 3.805, *p* < .001). No other significant differences were found (all *ps* > .002).

Among the 3,405 couples in Study 3 (Pairfam), 47.5% remained partnered through the five-year duration of this study, 46.7% were lost to attrition, and 5.8% terminated their relationship. Couples who ended their relationship reported significantly lower relationship satisfaction (male partner *M* = 7.75, *SD =* 2.06; female partner *M* = 7.62, *SD* = 2.26) than couples who remained together (male partner *M* = 8.43, *SD =* 1.91, *t*(1774) = 4.61, *p* < .001; female partner *M* = 8.34, *SD* = 2.00, *t*(224.70) = 4.22, *p* < .001) or attrited from the study (male partner *M* = 8.30, *SD =* 2.00, *t*(1719) = 3.57, *p* < .001; female partner *M* = 8.25, *SD* = 2.04, *t*(227.11) = 3.64, *p* < .001). No differences were found for dissolving couples relative to attriting and continuing couples in regard to Wave 1 reports of positive and negative communication and there were no differences between attriting and continuing couples (all *p*s > .003).

Across studies, these tests suggest there are systematic differences in relationship satisfaction and communication at baseline between couples who broke up compared to continually partnered and attrited couples. As such, we incorporated a variable representing whether the couple broke up as an auxiliary variable to aid in the accurate estimation of missing values (Graham, 2003).

Overall, we evaluated model fit with commonly used global fit indices: the chi-square test (χ^2^), the root mean square error of approximation (RMSEA), the comparative fit index (CFI), the Tucker-Lewis Index (TLI), and standardized root mean square residual (SRMR). A non-significant chi-square, values greater than .95 for CFI and TLI, and values smaller than .06 and .08 for RMSEA and SRMR are generally accepted criteria to suggest good model fit, while a CFI and TLI greater than .90 and RMSEA and SRMR smaller than .10 indicate acceptable fit (Little, 2013). We conducted all analyses using Mplus 8.0 (Muthén & Muthén, 1998-2017). In each study we evaluated attrition bias by comparing the couples who remained partnered through the study, those who attrited, and those who broke up on baseline reports of all study variables with a series of *t*-tests (corrected for family-wise error).

**3. Tests of Measurement Invariance**

To ensure that observed cross-construct associations in our analyses reflect true relationship satisfaction/communication links and are not due to inconsistent measurement, we tested measurement invariance over time and across partners following procedures outlined by Little (2013) for all multi-item self-report measures. For constructs with more than four items, longitudinal parceling procedures were used to create three indicators for the modeling (Little, 2013). A baseline model was first computed where factor loadings and intercepts of indicator variables were freely estimated. A weak invariance model was next specified that constrained factor loadings to equivalence across time and partners. The final test of measurement invariance constrained indicator intercepts to equality across time and across partners, referred to as the strong invariance model.

Models were evaluated according to the change in CFI because the chi-square difference test is overpowered when testing measurement invariance (Little, 2013; Widaman, Ferrer, & Conger, 2010). A change in CFI ≤ .01 indicates a non-substantive decrease in model fit. If the application of equality constraints resulted in a significant worsening of model fit, residuals and modification indices were consulted to diagnose the specific area of misfit. If partial invariance could be achieved by freeing a parameter, we did so and proceeded with the analysis (Widaman et al., 2010). Full model fit indices for these analyses are shown in Supplementary Table 8.

All constructs except relationship satisfaction in Study 1 achieved at least partial strong measurement invariance across time and partners. In the partial strong invariance models, typically one or more indicators were allowed to vary across partners, but were constrained to equality across time. Given that the same construct for both partners (e.g., male and female partner relationship satisfaction) was not included in the same analysis in any of our ALT-SR models, we proceeded with a reasonable degree of confidence that the reported cross-construct associations reflect links between the constructs and did not arise due to inconsistent measurement across time. For relationship satisfaction in Study 1, the residuals and modification indices did not reveal any specific parameter responsible for the misfit. To determine whether inconsistent measurement of this construct over time and across partners accounted for the observed associations in our analyses, we recomputed our ALT-SR models with a single-item assessment of satisfaction (“Please indicate the degree of happiness, all things considered, of your relationship”). The pattern of results obtained in the alternate ALT-SR model with the single item were consistent with those obtained from the full measure (see Supplementary Table 9). As such, we present results with the full four-item satisfaction measure in the manuscript.

Supplementary Table 8

*Model Fit Indices for the Tests of Measurement Invariance Across Time and Partners for Multi-Items Self-Report Measures*

| *Model* | χ^2^(df) | RMSEA (90% CI) | CFI | Model Comparison: ΔCFI |
| --- | --- | --- | --- | --- |
| **Study 1** | | | | |
| Negative Communication | | | | |
| Baseline | 401.413 (285) | .035 (.027, .043) | .976 |  |
| Weak Invariance | 421.656 (303) | .035 (.026, .042) | .975 | ΔCFI = .001 |
| **Strong Invariance** | **445.262 (323)** | **.034 (.026, .041)** | **.975** | ΔCFI = .000 |
| Relationship Satisfaction | | | | |
| **Baseline** | **1150.878 (595)** | **.053 (.049, .058)** | **.925** |  |
| Weak Invariance | 1492.038 (622) | .065 (.061, .070) | .883 | ΔCFI = .042 |
| Strong Invariance | 3333.507 (658) | .111 (.108, .115) | .640 | ΔCFI = .243 |
| **Study 2** | | | | |
| Positive Communication | | | | |
| Baseline | 729.264 (595) | .024 (.018, .030) | .978 |  |
| Weak Invariance | 751.605 (622) | .024 (.017, .029) | .979 | ΔCFI = .001 |
| Strong Invariance | 917.110 (658) | .032 (.027, .037) | .958 | ΔCFI = .021 |
| **Part. Strong Invar.** | **802.639 (657)** | **.024 (.018, .030)** | **.977** | ΔCFI = .002 |
| Negative Communication |  |  |  |  |
| Baseline | 354.798 (285) | .026 (.016, .034) | .988 |  |
| Weak Invariance | 387.708 (303) | .027 (.018, .035) | .986 | ΔCFI = .002 |
| Strong Invariance | 552.253 (330) | .042 (.036, .048) | .963 | ΔCFI = .023 |
| **Part. Strong Invar.** | **467.926 (323)** | **.035 (.027, .041)** | **.976** | ΔCFI = .010 |
| Relationship Satisfaction |  |  |  |  |
| Baseline | 779.746 (595) | .029 (.023, .034) | .980 |  |
| Weak Invariance | 827.774 (622) | .030 (.024, .035) | .978 | ΔCFI = .002 |
| **Strong Invariance** | **892.000 (658)** | **.031 (.025, .036)** | **.975** | ΔCFI = .003 |
| **Study 3** |  |  |  |  |
| Positive Communication |  |  |  |  |
| Baseline | 88.119 (75) | .007 (.000, .013) | .998 |  |
| Weak Invariance | 102.936 (84) | .008 (.000, .013) | .998 | ΔCFI = .000 |
| Strong Invariance | 513.839 (102) | .034 (.032, .037) | .953 | ΔCFI = .045 |
| **Part. Strong Invar.** | **145.050 (92)** | **.013 (.009, .017)** | **.994** | ΔCFI = .004 |
| Negative Communication |  |  |  |  |
| Baseline | 2099.561 (585) | .028 (.026, .029) | .958 |  |
| Weak Invariance | 2227.733 (612) | .028 (.027, .029) | .956 | ΔCFI = .002 |
| Strong Invariance | 2761.621 (648) | .031 (.030, .032) | .942 | ΔCFI = .014 |
| **Part. Strong Invar.** | 2364.007 (646) | .028 (.027, .029) | .953 | ΔCFI = .003 |

*Notes:* Bolded models have the best fit. Part. = Partial. Invar. = Invariance. Partial strong invariance was typically achieved by allowing one or more indicator intercepts to vary across partners, but not across time.

Supplementary Table 9

*Alternate Standardized Bivariate ALT-SR Modeling Results for Negative Communication and Single-Item Relationship Satisfaction Measure in the RDS Study (Study 1; n = 316 couples)*

| **Female Satisfaction** |  | **Within-Partner Comm. Model** | | | | |  | **Cross-Partner Comm. Model** | | | | |
| --- | --- | --- | --- | --- | --- | --- | --- | --- | --- | --- | --- | --- |
| **Between-Person Results** |  | 1. | 2. | 3. | 4. |  |  | 1. | 2. | 3. | 4. |  |
| 1. Neg. Intercept |  | ̶ |  |  |  |  |  | ̶ |  |  |  |  |
| 2. Neg. Slope |  | ̶ | ̶ |  |  |  |  | ̶ | ̶ |  |  |  |
| 3. F. Sat. Intercept |  | -.58* | ̶ | ̶ |  |  |  | -.48* | ̶ | ̶ |  |  |
| 4. F. Sat. Slope |  | -.24* | ̶ | -.23* | ̶ |  |  | -.07 | ̶ | -.29* | ̶ |  |
| **Within-Person Results** |  | W1 | W2 | W3 | W4 | W5 |  | W1 | W2 | W3 | W4 | W5 |
| Cross-Lagged Paths |  |  |  |  |  |  |  |  |  |  |  |  |
| Neg._W-1_ → F. Sat. |  | ̶ | **-.10*^a^** | **-.09*^a^** | **-.12*^a^** | **-.12*^a^** |  | ̶ | -.02^f^ | -.02^f^ | -.02^f^ | -.02^f^ |
| F. Sat. _W-1_ → Neg. |  | ̶ | **-**.05^b^ | -.08^b^ | -.07^b^ | -.06^b^ |  | ̶ | -.02^g^ | -.03^g^ | -.03^g^ | -.02^g^ |
| Autoregressive Paths |  |  |  |  |  |  |  |  |  |  |  |  |
| Negative |  | ̶ | .12^c^ | .09^c^ | .09^c^ | .10^c^ |  | ̶ | .16*^f^ | .14*^f^ | .14*^f^ | .12*^f^ |
| F. Satisfaction |  | ̶ | -.01^d^ | -.01^d^ | -.01^d^ | -.01^d^ |  | ̶ | -.01^g^ | -.01^g^ | -.02^g^ | -.02^g^ |
| Concurrent Correlations |  |  |  |  |  |  |  |  |  |  |  |  |
| Neg. ↔ F. Sat. |  | -.42*^e^ | -.35*^e^ | -.37*^e^ | -.44*^e^ | -.45*^e^ |  | -.18*^h^ | -.13*^h^ | -.14*^h^ | -.19*^h^ | -.19*^h^ |
|  |  |  |  |  |  |  |  |  |  |  |  |  |
| **Male Satisfaction** |  | **Within-Partner Comm. Model** | | | | |  | **Cross-Partner Comm. Model** | | | | |
| **Between-Person Results** |  | 1. | 2. | 3. | 4. |  |  | 1. | 2. | 3. | 4. |  |
| 1. Neg. Intercept |  | ̶ |  |  |  |  |  | ̶ |  |  |  |  |
| 2. Neg. Slope |  | ̶ | ̶ |  |  |  |  | ̶ | ̶ |  |  |  |
| 3. M. Sat. Intercept |  | -.60* | ̶ | ̶ |  |  |  | -.39* | ̶ | ̶ |  |  |
| 4. M. Sat. Slope |  | -.18 | ̶ | -.06 | ̶ |  |  | -.13 | ̶ | -.10 | ̶ |  |
| **Within-Person Results** |  | W1 | W2 | W3 | W4 | W5 |  | W1 | W2 | W3 | W4 | W5 |
| Cross-Lagged Paths |  |  |  |  |  |  |  |  |  |  |  |  |
| Neg._W-1_ → M. Sat. |  | ̶ | -.04^i^ | -.04^i^ | -.04^i^ | -.05^i^ |  | ̶ | -.04^m^ | -.03^m^ | -.04^m^ | -.05^m^ |
| M. Sat. _W-1_ → Neg. |  | ̶ | **-.27*** | -.03 | .00 | **-.35*** |  | ̶ | -.01^n^ | -.01^n^ | -.01^n^ | -.01^n^ |
| Autoregressive Paths |  |  |  |  |  |  |  |  |  |  |  |  |
| Negative |  | ̶ | .13^j^ | .11^j^ | .11^j^ | .09^j^ |  | ̶ | .15*^o^ | .13*^o^ | .11*^o^ | .15*^o^ |
| M. Satisfaction |  | ̶ | .21*^k^ | .25*^k^ | .24*^k^ | .30*^k^ |  | ̶ | .16*^p^ | .19*^p^ | .19*^p^ | .22*^p^ |
| Concurrent Correlations |  |  |  |  |  |  |  |  |  |  |  |  |
| Neg. ↔ M. Sat. |  | -.25*^L^ | -.29*^L^ | -.31*^L^ | -.31*^L^ | -.37*^L^ |  | -.12*^q^ | -.13*^q^ | -.15*^q^ | -.15*^q^ | -.21*^q^ |

*Notes.* Standardized estimates. ^a - q^Corresponding coefficients are constrained to equality. Significant within-person cross-lagged paths are bolded for clarity. Negative communication slope variance was fixed to 0. _W-1_Preceding Wave. F. = Female partner. M. = Male partner. Neg. = Negative communication. Sat. = Relationship satisfaction. W = Wave. The intercepts and slopes (except the negative communication slope) were regressed on relationship duration. Female relationship satisfaction and negative communication fit indices: χ^2^(47) = 80.965; RMSEA = .048 (.029, .065); CFI = .977; TLI = .973; SRMR = .055. Female relationship satisfaction and male negative communication fit indices: χ^2^(47) = 51.821; RMSEA = .018 (.000, .042); CFI = .996; TLI = .995; SRMR = .046. Male relationship satisfaction and negative communication fit indices: χ^2^(44) = 65.437; RMSEA = .039 (.016, .058); CFI = .983; TLI = .979; SRMR = .054. Male relationship satisfaction and female negative communication fit indices: χ^2^(47) = 65.145; RMSEA = .035 (.008, .054); CFI = .985; TLI = .982; SRMR = .054. **p* < .05.

**4. Full ALT-SR Model Results**

Supplementary Table 10

*Standardized Bivariate ALT-SR Modeling Results for Negative Communication and Relationship Satisfaction in the RDS Study (Study 1; n = 316 couples)*

| **Female Satisfaction** |  | **Within-Partner Comm. Model** | | | | |  | **Cross-Partner Comm. Model** | | | | |
| --- | --- | --- | --- | --- | --- | --- | --- | --- | --- | --- | --- | --- |
| **Between-Person Results** |  | 1. | 2. | 3. | 4. |  |  | 1. | 2. | 3. | 4. |  |
| 1. Neg. Intercept |  | ̶ |  |  |  |  |  | ̶ |  |  |  |  |
| 2. Neg. Slope |  | ̶ | ̶ |  |  |  |  | ̶ | ̶ |  |  |  |
| 3. F. Sat. Intercept |  | -.68* | ̶ | ̶ |  |  |  | -.54* | ̶ | ̶ |  |  |
| 4. F. Sat. Slope |  | -.46* | ̶ | .25 | ̶ |  |  | -.19 | ̶ | .14 | ̶ |  |
| **Within-Person Results** |  | W1 | W2 | W3 | W4 | W5 |  | W1 | W2 | W3 | W4 | W5 |
| Cross-Lagged Paths |  |  |  |  |  |  |  |  |  |  |  |  |
| Neg._W-1_ → F. Sat. |  | ̶ | **-.15*^a^** | **-.12*^a^** | **-.18*^a^** | **-.13*^a^** |  | ̶ | -.08^f^ | -.06^f^ | -.09^f^ | -.07^f^ |
| F. Sat. _W-1_ → Neg. |  | ̶ | **-**.10^b^ | -.09^b^ | -.08^b^ | -.07^b^ |  | ̶ | -.02^g^ | -.02^g^ | -.02^g^ | -.01^g^ |
| Autoregressive Paths |  |  |  |  |  |  |  |  |  |  |  |  |
| Negative |  | ̶ | .08^c^ | .06^c^ | .06^c^ | .07^c^ |  | ̶ | .17*^f^ | .15*^f^ | .15*^f^ | .13*^f^ |
| F. Satisfaction |  | ̶ | -.01^d^ | -.01^d^ | -.01^d^ | -.01^d^ |  | ̶ | .02^g^ | .02^g^ | .03^g^ | .01^g^ |
| Concurrent Correlations |  |  |  |  |  |  |  |  |  |  |  |  |
| Neg. ↔ F. Sat. |  | -.39*^e^ | -.51*^e^ | -.48*^e^ | -.62*^e^ | -.45*^e^ |  | -.17*^h^ | -.20*^h^ | -.20*^h^ | -.29*^h^ | -.18*^h^ |
|  |  |  |  |  |  |  |  |  |  |  |  |  |
| **Male Satisfaction** |  | **Within-Partner Comm. Model** | | | | |  | **Cross-Partner Comm. Model** | | | | |
| **Between-Person Results** |  | 1. | 2. | 3. | 4. |  |  | 1. | 2. | 3. | 4. |  |
| 1. Neg. Intercept |  | ̶ |  |  |  |  |  | ̶ |  |  |  |  |
| 2. Neg. Slope |  | ̶ | ̶ |  |  |  |  | ̶ | ̶ |  |  |  |
| 3. M. Sat. Intercept |  | -.66* | ̶ | ̶ |  |  |  | -.47* | ̶ | ̶ |  |  |
| 4. M. Sat. Slope |  | -.41* | ̶ | .45 | ̶ |  |  | -.42* | ̶ | .66 | ̶ |  |
| **Within-Person Results** |  | W1 | W2 | W3 | W4 | W5 |  | W1 | W2 | W3 | W4 | W5 |
| Cross-Lagged Paths |  |  |  |  |  |  |  |  |  |  |  |  |
| Neg._W-1_ → M. Sat. |  | ̶ | .01 | .21 | .02 | **-.24*** |  | ̶ | -.07^k^ | -.07^k^ | -.07^k^ | -.07^k^ |
| M. Sat. _W-1_ → Neg. |  | ̶ | -.02 | .10 | **.25*** | **-.35*** |  | ̶ | .00^L^ | .00^L^ | .00^L^ | .00^L^ |
| Autoregressive Paths |  |  |  |  |  |  |  |  |  |  |  |  |
| Negative |  | ̶ | .17*^i^ | .15*^i^ | .14*^i^ | .12*^i^ |  | ̶ | .16*^m^ | .13*^m^ | .11*^m^ | .15*^m^ |
| M. Satisfaction |  | ̶ | .20*^j^ | .28*^j^ | .20*^j^ | .22*^j^ |  | ̶ | .20*^n^ | .26*^n^ | .21*^n^ | .22*^n^ |
| Concurrent Correlations |  |  |  |  |  |  |  |  |  |  |  |  |
| Neg. ↔ M. Sat. |  | -.37* | -.33* | -.12 | -.29* | -.71* |  | -.16*^o^ | -.19*^o^ | -.22*^o^ | -.19*^o^ | -.24*^o^ |

*Notes.* Standardized estimates. ^a - o^Corresponding coefficients are constrained to equality. Significant within-person cross-lagged paths are bolded for clarity. Negative communication slope variance was fixed to 0. _W-1_Preceding Wave. F. = Female partner. M. = Male partner. Neg. = Negative communication. Sat. = Relationship satisfaction. W = Wave. The intercepts and slopes (except the negative communication slope) were regressed on relationship duration. Female relationship satisfaction and negative communication fit indices: χ^2^(47) = 92.724; RMSEA = .055 (.039, .072); CFI = .977; TLI = .973; SRMR = .058. Female relationship satisfaction and male negative communication fit indices: χ^2^(47) = 77.986; RMSEA = .046 (.027, .063); CFI = .981; TLI = .978; SRMR = .050. Male relationship satisfaction and negative communication fit indices: χ^2^(36) = 70.719; RMSEA = .055 (.036, .074); CFI = .978; TLI = .967; SRMR = .065. Male relationship satisfaction and female negative communication fit indices: χ^2^(46) = 80.008; RMSEA = .048 (.030, .066); CFI = .977; TLI = .973; SRMR = .066. **p* < .05.

Supplementary Table 11

*Standardized Bivariate ALT-SR Modeling Results for Observed Positive Communication and Relationship Satisfaction in the PASEZ Study (Study 2; n = 365 couples)*

| **Female Satisfaction** |  | **Within-Partner Comm. Model** | | | | |  | **Cross-Partner Comm. Model** | | | | |
| --- | --- | --- | --- | --- | --- | --- | --- | --- | --- | --- | --- | --- |
| **Between-Person Results** |  | 1. | 2. | 3. | 4. |  |  | 1. | 2. | 3. | 4. |  |
| 1. Pos. Intercept |  | ̶ |  |  |  |  |  | ̶ |  |  |  |  |
| 2. Pos. Slope |  | ̶ | ̶ |  |  |  |  | -.74* | ̶ |  |  |  |
| 3. F. Sat. Intercept |  | .23* | ̶ | ̶ |  |  |  | .17* | -.01 | ̶ |  |  |
| 4. F. Sat. Slope |  | .15 | ̶ | .15 | ̶ |  |  | .10 | .00 | .15 | ̶ |  |
| **Within-Person Results** |  | W1 | W2 | W3 | W4 | W5 |  | W1 | W2 | W3 | W4 | W5 |
| Cross-Lagged Paths |  |  |  |  |  |  |  |  |  |  |  |  |
| Pos._W-1_ → F. Sat. |  | ̶ | -.05^a^ | -.03^a^ | -.03^a^ | -.04^a^ |  | ̶ | .00^f^ | .00^f^ | .00^f^ | .00^f^ |
| F. Sat. _W-1_ → Pos. |  | ̶ | -.05^b^ | -.08^b^ | -.07^b^ | -.07^b^ |  | ̶ | .02^g^ | .04^g^ | .02^g^ | .02^g^ |
| Autoregressive Paths |  |  |  |  |  |  |  |  |  |  |  |  |
| Positive |  | ̶ | .23*^c^ | .16*^c^ | .12*^c^ | .17*^c^ |  | ̶ | .01^h^ | .01^h^ | .00^h^ | .01^h^ |
| F. Satisfaction |  | ̶ | .04^d^ | .05^d^ | .06^d^ | .05^d^ |  | ̶ | .04^i^ | .05^i^ | .06^i^ | .05^i^ |
| Concurrent Correlations |  |  |  |  |  |  |  |  |  |  |  |  |
| Pos. ↔ F. Sat. |  | -.04^e^ | -.04^e^ | -.04^e^ | -.03^e^ | -.04^e^ |  | .03^j^ | .02^j^ | .02^j^ | .02^j^ | .02^j^ |
|  |  |  | | | | |  |  | | | | |
| **Male Satisfaction** |  | **Within-Partner Comm. Model** | | | | |  | **Cross-Partner Comm. Model** | | | | |
| **Between-Person Results** |  | 1. | 2. | 3. | 4. |  |  | 1. | 2. | 3. | 4. |  |
| 1. Pos. Intercept |  | ̶ |  |  |  |  |  | ̶ |  |  |  |  |
| 2. Pos. Slope |  | -.76* | ̶ |  |  |  |  | ̶ | ̶ |  |  |  |
| 3. M. Sat. Intercept |  | .13 | -.05 | ̶ |  |  |  | -.04 | ̶ | ̶ |  |  |
| 4. M. Sat. Slope |  | .05 | .24 | -.10 | ̶ |  |  | .44* | ̶ | -.08 | ̶ |  |
| **Within-Person Results** |  | W1 | W2 | W3 | W4 | W5 |  | W1 | W2 | W3 | W4 | W5 |
| Cross-Lagged Paths |  |  |  |  |  |  |  |  |  |  |  |  |
| Pos._W-1_ → M. Sat. |  | ̶ | .05^k^ | .04^k^ | .03^k^ | .06^k^ |  | ̶ | .10^o^ | .06^o^ | .06^o^ | .09^o^ |
| M. Sat. _W-1_ → Pos. |  | ̶ | -.03^L^ | -.04^L^ | -.02^L^ | -.02^L^ |  | ̶ | .16 | .08 | **.21*** | **-.25*** |
| Autoregressive Paths |  |  |  |  |  |  |  |  |  |  |  |  |
| Positive |  | ̶ | .01^m^ | .01^m^ | .01^m^ | .01^m^ |  | ̶ | .21*^p^ | .14*^p^ | .11*^p^ | .16*^p^ |
| M. Satisfaction |  | ̶ | .03^n^ | .04^n^ | .04^n^ | .04^n^ |  | ̶ | .08^q^ | .09*^q^ | .09*^q^ | .10*^q^ |
| Concurrent Correlations |  |  |  |  |  |  |  |  |  |  |  |  |
| Pos. ↔ M. Sat. |  | .04^o^ | .05^o^ | .05^o^ | .03^o^ | .04^o^ |  | .25* | .23* | .02 | .12 | -.21 |

*Notes.* Standardized estimates. ^a - p^Corresponding coefficients are constrained to equality. Significant within-person cross-lagged paths are bolded for clarity. The female partner positive communication slope variance was fixed to 0. _W-1_Preceding Wave. F. = Female partner. M. = Male partner. Pos. = Positive communication. Sat. = Relationship satisfaction. W = Wave. The intercepts and slopes were regressed on relationship duration. Female relationship satisfaction and positive communication fit indices: χ^2^(46) = 37.289; RMSEA = .000 (.000, .022); CFI = 1.000; TLI = 1.011; SRMR = .040. Female relationship satisfaction and male positive communication fit indices: χ^2^(42) = 27.738; RMSEA = .000 (.000, .000); CFI = 1.000; TLI = 1.018; SRMR = .031. Male relationship satisfaction and positive communication fit indices: χ^2^(42) = 42.303; RMSEA = .004 (.000, .036); CFI = 1.000; TLI = 1.000; SRMR = .065. Male relationship satisfaction and female positive communication fit indices: χ^2^(39) = 47.659; RMSEA = .025 (.000, .046); CFI = .992; TLI = .988; SRMR = .075. **p* < .05.

Supplementary Table 12

*Standardized Bivariate ALT-SR Modeling Results for Observed Negative Communication and Relationship Satisfaction in the PASEZ Study (Study 2; n = 365 couples)*

| **Female Satisfaction** |  | **Within-Partner Comm. Model** | | | | |  | **Cross-Partner Comm. Model** | | | | |
| --- | --- | --- | --- | --- | --- | --- | --- | --- | --- | --- | --- | --- |
| **Between-Person Results** |  | 1. | 2. | 3. | 4. |  |  | 1. | 2. | 3. | 4. |  |
| 1. Neg. Intercept |  | ̶ |  |  |  |  |  | ̶ |  |  |  |  |
| 2. Neg. Slope |  | ̶ | ̶ |  |  |  |  | ̶ | ̶ |  |  |  |
| 3. F. Sat. Intercept |  | -.41* | ̶ | ̶ |  |  |  | -.36* | ̶ | ̶ |  |  |
| 4. F. Sat. Slope |  | -.23 | ̶ | .17 | ̶ |  |  | -.02 | ̶ | .11 | ̶ |  |
| **Within-Person Results** |  | W1 | W2 | W3 | W4 | W5 |  | W1 | W2 | W3 | W4 | W5 |
| Cross-Lagged Paths |  |  |  |  |  |  |  |  |  |  |  |  |
| Neg._W-1_ → F. Sat. |  | ̶ | -.06^a^ | -.05^a^ | -.09^a^ | -.08^a^ |  | ̶ | .01^e^ | .01^e^ | .02^e^ | .02^e^ |
| F. Sat. _W-1_ → Neg. |  | ̶ | -.05^b^ | -.04^b^ | -.05^b^ | -.04^b^ |  | ̶ | .03^f^ | .03^f^ | .03^f^ | .03^f^ |
| Autoregressive Paths |  |  |  |  |  |  |  |  |  |  |  |  |
| Negative |  | ̶ | -.25 | .20* | .39* | .13 |  | ̶ | -.48* | .31* | .22*^f^ | .25*^f^ |
| F. Satisfaction |  | ̶ | .04^c^ | .05^c^ | .06^c^ | .05^c^ |  | ̶ | .04^g^ | .06^g^ | .07^g^ | .06^g^ |
| Concurrent Correlations |  |  |  |  |  |  |  |  |  |  |  |  |
| Neg. ↔ F. Sat. |  | -.22*^d^ | -.19*^d^ | -.11*^d^ | -.13*^d^ | -.11*^d^ |  | -.09^h^ | -.05^h^ | -.04^h^ | -.03^h^ | -.03^h^ |
|  |  |  |  |  |  |  |  |  |  |  |  |  |
| **Male Satisfaction** |  | **Within-Partner Comm. Model** | | | | |  | **Cross-Partner Comm. Model** | | | | |
| **Between-Person Results** |  | 1. | 2. | 3. | 4. |  |  | 1. | 2. | 3. | 4. |  |
| 1. Neg. Intercept |  | ̶ |  |  |  |  |  | ̶ |  |  |  |  |
| 2. Neg. Slope |  | ̶ | ̶ |  |  |  |  | ̶ | ̶ |  |  |  |
| 3. M. Sat. Intercept |  | -.20* | ̶ | ̶ |  |  |  | -.25* | ̶ | ̶ |  |  |
| 4. M. Sat. Slope |  | .01 | ̶ | -.10 | ̶ |  |  | -.20 | ̶ | -.12 | ̶ |  |
| **Within-Person Results** |  | W1 | W2 | W3 | W4 | W5 |  | W1 | W2 | W3 | W4 | W5 |
| Cross-Lagged Paths |  |  |  |  |  |  |  |  |  |  |  |  |
| Neg._W-1_ → M. Sat. |  | ̶ | -.04^i^ | -.05^i^ | -.07^i^ | -.09^i^ |  | ̶ | -.02^m^ | -.02^m^ | -.02^m^ | -.02^m^ |
| M. Sat. _W-1_ → Neg. |  | ̶ | .02^j^ | .01^j^ | .01^j^ | .01^j^ |  | ̶ | **-.12*^n^** | **-.10*^n^** | **-.17*^n^** | **-.19*^n^** |
| Autoregressive Paths |  |  |  |  |  |  |  |  |  |  |  |  |
| Negative |  | ̶ | -.52* | .31* | .22* | .25* |  | ̶ | -.29 | .17 | .36* | .13 |
| M. Satisfaction |  | ̶ | .03^k^ | .03^k^ | .03^k^ | .04^k^ |  | ̶ | .01^o^ | .01^o^ | .01^o^ | .01^o^ |
| Concurrent Correlations |  |  |  |  |  |  |  |  |  |  |  |  |
| Neg. ↔ M. Sat. |  | -.11^L^ | -.09^L^ | -.06^L^ | -.05^L^ | -.06^L^ |  | -.27*^p^ | -.30*^p^ | -.17*^p^ | -.20*^p^ | -.20*^p^ |

*Notes.* Standardized estimates. ^a - p^Corresponding coefficients are constrained to equality. Significant within-person cross-lagged paths are bolded for clarity. The negative communication slope variances for male and female partners were fixed to 0. _W-1_Preceding Wave. F. = Female partner. M. = Male partner. Pos. = Positive communication. Sat. = Relationship satisfaction. W = Wave. The intercepts and slopes were regressed on relationship duration. Female relationship satisfaction and negative communication fit indices: χ^2^(43) = 42.347; RMSEA = .000 (.000, .034); CFI = 1.000; TLI = 1.001; SRMR = .045. Female relationship satisfaction and male negative communication fit indices: χ^2^(43) = 50.078; RMSEA = .021 (.000, .043); CFI = .994; TLI = .992; SRMR = .042. Male relationship satisfaction and negative communication fit indices: χ^2^(43) = 55.766; RMSEA = .028 (.000, .048); CFI = .989; TLI = .986; SRMR = .069. Male relationship satisfaction and female negative communication fit indices: χ^2^(43) = 41.778; RMSEA = .000 (.000, .034); CFI = 1.000; TLI = 1.001; SRMR = .068. **p* < .05.

Supplementary Table 13

*Standardized Bivariate ALT-SR Modeling Results for Self-Reported Positive Communication and Relationship Satisfaction in the PASEZ Study (Study 2; n = 365 couples)*

| **Female Satisfaction** |  | **Within-Partner Comm. Model** | | | | |  | **Cross-Partner Comm. Model** | | | | |
| --- | --- | --- | --- | --- | --- | --- | --- | --- | --- | --- | --- | --- |
| **Between-Person Results** |  | 1. | 2. | 3. | 4. |  |  | 1. | 2. | 3. | 4. |  |
| 1. Pos. Intercept |  | ̶ |  |  |  |  |  | ̶ |  |  |  |  |
| 2. Pos. Slope |  | -.35* | ̶ |  |  |  |  | -.11 | ̶ |  |  |  |
| 3. F. Sat. Intercept |  | .24* | .24* | ̶ |  |  |  | .41* | -.31* | ̶ |  |  |
| 4. F. Sat. Slope |  | .07 | .24 | .16 | ̶ |  |  | .04 | .44 | .17 | ̶ |  |
| **Within-Person Results** |  | W1 | W2 | W3 | W4 | W5 |  | W1 | W2 | W3 | W4 | W5 |
| Cross-Lagged Paths |  |  |  |  |  |  |  |  |  |  |  |  |
| Pos._W-1_ → F. Sat. |  | ̶ | -.01^a^ | -.02^a^ | -.02^a^ | -.02^a^ |  | ̶ | -.05^f^ | -.04^f^ | -.04^f^ | -.04^f^ |
| F. Sat. _W-1_ → Pos. |  | ̶ | -.03^b^ | -.04^b^ | -.04^b^ | -.05^b^ |  | ̶ | .00^g^ | .00^g^ | .00^g^ | .00^g^ |
| Autoregressive Paths |  |  |  |  |  |  |  |  |  |  |  |  |
| Positive |  | ̶ | .05^c^ | .10^c^ | .07^c^ | .11^c^ |  | ̶ | -.06^h^ | -.05^h^ | -.05^h^ | -.06^h^ |
| F. Satisfaction |  | ̶ | .04^d^ | .05^d^ | .06^d^ | .05^d^ |  | ̶ | .04^i^ | .06^i^ | .06^i^ | .06^i^ |
| Concurrent Correlations |  |  |  |  |  |  |  |  |  |  |  |  |
| Pos. ↔ F. Sat. |  | .10^e^ | .05^e^ | .05^e^ | .04^e^ | .05^e^ |  | .00^j^ | .00^j^ | .00^j^ | .00^j^ | .00^j^ |
|  |  |  | | | | |  |  | | | | |
| **Male Satisfaction** |  | **Within-Partner Comm. Model** | | | | |  | **Cross-Partner Comm. Model** | | | | |
| **Between-Person Results** |  | 1. | 2. | 3. | 4. |  |  | 1. | 2. | 3. | 4. |  |
| 1. Pos. Intercept |  | ̶ |  |  |  |  |  | ̶ |  |  |  |  |
| 2. Pos. Slope |  | -.11 | ̶ |  |  |  |  | -.33* | ̶ |  |  |  |
| 3. M. Sat. Intercept |  | .44* | -.18 | ̶ |  |  |  | .10 | .25* | ̶ |  |  |
| 4. M. Sat. Slope |  | .12 | .13 | -.12 | ̶ |  |  | .04 | .03 | -.12 | ̶ |  |
| **Within-Person Results** |  | W1 | W2 | W3 | W4 | W5 |  | W1 | W2 | W3 | W4 | W5 |
| Cross-Lagged Paths |  |  |  |  |  |  |  |  |  |  |  |  |
| Pos._W-1_ → M. Sat. |  | ̶ | -.05^k^ | -.05^k^ | -.05^k^ | -.07^k^ |  | ̶ | .03^o^ | .05^o^ | .05^o^ | .07^o^ |
| M. Sat. _W-1_ → Pos. |  | ̶ | .03^L^ | .03^L^ | .03^L^ | .03^L^ |  | ̶ | .02^p^ | .03^p^ | .02^p^ | .03^p^ |
| Autoregressive Paths |  |  |  |  |  |  |  |  |  |  |  |  |
| Positive |  | ̶ | -.05^m^ | -.05^m^ | -.05^m^ | -.05^m^ |  | ̶ | .06^q^ | .11^q^ | .07^q^ | .13^q^ |
| M. Satisfaction |  | ̶ | .02^n^ | .02^n^ | .03^n^ | .03^n^ |  | ̶ | .03^r^ | .03^r^ | .03^r^ | .04^r^ |
| Concurrent Correlations |  |  |  |  |  |  |  |  |  |  |  |  |
| Pos. ↔ M. Sat. |  | .09^o^ | .09^o^ | .08^o^ | .08^o^ | .10^o^ |  | .14^s^ | .08^s^ | .09^s^ | .07^s^ | .11^s^ |

*Notes.* Standardized estimates. ^a - p^Corresponding coefficients are constrained to equality. _W-1_Preceding Wave. F. = Female partner. M. = Male partner. Pos. = Positive communication. Sat. = Relationship satisfaction. W = Wave. The intercepts and slopes were regressed on relationship duration. Female relationship satisfaction and positive communication fit indices: χ^2^(42) = 50.831; RMSEA = .024 (.000, .045); CFI = .994; TLI = .992; SRMR = .032. Female relationship satisfaction and male positive communication fit indices: χ^2^(42) = 27.843; RMSEA = .000 (.000, .000); CFI = 1.000; TLI = 1.011; SRMR = .055. Male relationship satisfaction and positive communication fit indices: χ^2^(42) = 42.148; RMSEA = .003 (.000, .036); CFI = 1.000; TLI = 1.000; SRMR = .078. Male relationship satisfaction and female positive communication fit indices: χ^2^(42) = 46.956; RMSEA = .018 (.000, .041); CFI = .997; TLI = .996; SRMR = .064. **p* < .05.

Supplementary Table 14

*Standardized Bivariate ALT-SR Modeling Results for Self-Reported Negative Communication and Relationship Satisfaction in the PASEZ Study (Study 2; n = 365 couples)*

| **Female Satisfaction** |  | **Within-Partner Comm. Model** | | | | |  | **Cross-Partner Comm. Model** | | | | |
| --- | --- | --- | --- | --- | --- | --- | --- | --- | --- | --- | --- | --- |
| **Between-Person Results** |  | 1. | 2. | 3. | 4. |  |  | 1. | 2. | 3. | 4. |  |
| 1. Neg. Intercept |  | ̶ |  |  |  |  |  | ̶ |  |  |  |  |
| 2. Neg. Slope |  | -.42* | ̶ |  |  |  |  | -.40* | ̶ |  |  |  |
| 3. F. Sat. Intercept |  | -.46* | .03 | ̶ |  |  |  | -.41* | .11 | ̶ |  |  |
| 4. F. Sat. Slope |  | -.12 | -.18 | .15 | ̶ |  |  | -.20 | .37 | .17 | ̶ |  |
| **Within-Person Results** |  | W1 | W2 | W3 | W4 | W5 |  | W1 | W2 | W3 | W4 | W5 |
| Cross-Lagged Paths |  |  |  |  |  |  |  |  |  |  |  |  |
| Neg._W-1_ → F. Sat. |  | ̶ | .04^a^ | .03^a^ | .04^a^ | .03^a^ |  | ̶ | **-.16*^f^** | **-.11*^f^** | **-.14*^f^** | **-.10*^f^** |
| F. Sat. _W-1_ → Neg. |  | ̶ | .03^b^ | .03^b^ | .02^b^ | .02^b^ |  | ̶ | **-.13*^g^** | **-.13*^g^** | **-.18*^g^** | **-.17*^g^** |
| Autoregressive Paths |  |  |  |  |  |  |  |  |  |  |  |  |
| Negative |  | ̶ | .02^c^ | .02^c^ | .02^c^ | .02^c^ |  | ̶ | -.07^h^ | -.04^h^ | -.07^h^ | -.05^h^ |
| F. Satisfaction |  | ̶ | .04^d^ | .05^d^ | .06^d^ | .05^d^ |  | ̶ | .02^i^ | .03^i^ | .03^i^ | .03^i^ |
| Concurrent Correlations |  |  |  |  |  |  |  |  |  |  |  |  |
| Neg. ↔ F. Sat. |  | -.24*^e^ | -.20*^e^ | -.17*^e^ | -.19*^e^ | -.19*^e^ |  | -.20*^j^ | -.21*^j^ | -.16*^j^ | -.22*^j^ | -.19*^j^ |
|  |  |  |  |  |  |  |  |  |  |  |  |  |
| **Male Satisfaction** |  | **Within-Partner Comm. Model** | | | | |  | **Cross-Partner Comm. Model** | | | | |
| **Between-Person Results** |  | 1. | 2. | 3. | 4. |  |  | 1. | 2. | 3. | 4. |  |
| 1. Neg. Intercept |  | ̶ |  |  |  |  |  | ̶ |  |  |  |  |
| 2. Neg. Slope |  | -.40* | ̶ |  |  |  |  | -.43* | ̶ |  |  |  |
| 3. M. Sat. Intercept |  | -.48* | .31* | ̶ |  |  |  | -.32* | .23* | ̶ |  |  |
| 4. M. Sat. Slope |  | -.06 | -.11 | -.11 | ̶ |  |  | -.10 | -.44* | -.12 | ̶ |  |
| **Within-Person Results** |  | W1 | W2 | W3 | W4 | W5 |  | W1 | W2 | W3 | W4 | W5 |
| Cross-Lagged Paths |  |  |  |  |  |  |  |  |  |  |  |  |
| Neg._W-1_ → M. Sat. |  | ̶ | **-.14*^k^** | **-.10*^k^** | **-.13*^k^** | **-.11*^k^** |  | ̶ | **.28*** | .06^p^ | -.01^p^ | -.11^p^ |
| M. Sat. _W-1_ → Neg. |  | ̶ | **-.19*^L^** | **-.17*^L^** | **-.25*^L^** | **-.23*^L^** |  | ̶ | .00^p^ | .00^p^ | .00^p^ | .00^p^ |
| Autoregressive Paths |  |  |  |  |  |  |  |  |  |  |  |  |
| Negative |  | ̶ | -.06^m^ | -.04^m^ | -.06^m^ | -.04^m^ |  | ̶ | -.01^q^ | -.01^q^ | -.01^q^ | -.01^q^ |
| M. Satisfaction |  | ̶ | .03^n^ | .03^n^ | .03^n^ | .04^n^ |  | ̶ | .03^r^ | .03^r^ | .03^r^ | .03^r^ |
| Concurrent Correlations |  |  |  |  |  |  |  |  |  |  |  |  |
| Neg. ↔ M. Sat. |  | -.23*^o^ | -.28*^o^ | -.20*^o^ | -.28*^o^ | -.32*^o^ |  | -.09^s^ | -.09^s^ | -.08^s^ | -.09^s^ | -.11^s^ |

*Notes.* Standardized estimates. ^a - p^Corresponding coefficients are constrained to equality. Significant within-person cross-lagged paths are bolded for clarity. _W-1_Preceding Wave. F. = Female partner. M. = Male partner. Pos. = Positive communication. Sat. = Relationship satisfaction. W = Wave. The intercepts and slopes were regressed on relationship duration. Female relationship satisfaction and negative communication fit indices: χ^2^(42) = 38.228; RMSEA = .000 (.000, .031); CFI = 1.000; TLI = 1.003; SRMR = .028. Female relationship satisfaction and male negative communication fit indices: χ^2^(42) = 33.582; RMSEA = .000 (.000, .023); CFI = 1.000; TLI = 1.006; SRMR = .031. Male relationship satisfaction and negative communication fit indices: χ^2^(42) = 42.739; RMSEA = .007 (.000, .036); CFI = 1.000; TLI = 1.000; SRMR = .060. Male relationship satisfaction and female negative communication fit indices: χ^2^(39) = 36.175; RMSEA = .000 (.000, .032); CFI = 1.000; TLI = 1.002; SRMR = .058. **p* < .05.

Supplementary Table 15

*Standardized Bivariate ALT-SR Modeling Results for Positive Communication and Relationship Satisfaction in the Pairfam Study (Study 3; n = 3,405 couples)*

| **Female Satisfaction** |  | **Within-Partner Comm. Model** | | | | |  | **Cross-Partner Comm. Model** | | | | |
| --- | --- | --- | --- | --- | --- | --- | --- | --- | --- | --- | --- | --- |
| **Between-Person Results** |  | 1. | 2. | 3. | 4. |  |  | 1. | 2. | 3. | 4. |  |
| 1. Pos. Intercept |  | ̶ |  |  |  |  |  | ̶ |  |  |  |  |
| 2. Pos. Slope |  | -.31* | ̶ |  |  |  |  | -.06 | ̶ |  |  |  |
| 3. F. Sat. Intercept |  | .22* | .06 | ̶ |  |  |  | .25* | -.08 | ̶ |  |  |
| 4. F. Sat. Slope |  | .09 | .09 | -.31* | ̶ |  |  | -.02 | .18 | -.31* | ̶ |  |
| **Within-Person Results** |  | W1 | W2 | W3 | W4 | W5 |  | W1 | W2 | W3 | W4 | W5 |
| Cross-Lagged Paths |  |  |  |  |  |  |  |  |  |  |  |  |
| Pos._W-1_ → F. Sat. |  | ̶ | .00^a^ | .00^a^ | .00^a^ | .00^a^ |  | ̶ | .03^e^ | .02^e^ | .02^e^ | .02^e^ |
| F. Sat. _W-1_ → Pos. |  | ̶ | .00^b^ | .00^b^ | .00^b^ | .00^b^ |  | ̶ | .02^f^ | .02^f^ | .02^f^ | .02^f^ |
| Autoregressive Paths |  |  |  |  |  |  |  |  |  |  |  |  |
| Positive |  | ̶ | -.18* | .01 | .08* | .10* |  | ̶ | .03^g^ | .03^g^ | .03^g^ | .03^g^ |
| F. Satisfaction |  | ̶ | .05*^c^ | .05*^c^ | .05*^c^ | .05*^c^ |  | ̶ | .05*^h^ | .05*^h^ | .05*^h^ | .05*^h^ |
| Concurrent Correlations |  |  |  |  |  |  |  |  |  |  |  |  |
| Pos. ↔ F. Sat. |  | .06*^d^ | .06*^d^ | .06*^d^ | .06*^d^ | .07*^d^ |  | .05*^i^ | .05*^i^ | .05*^i^ | .05*^i^ | .06*^i^ |
|  |  |  | | | | |  |  | | | | |
| **Male Satisfaction** |  | **Within-Partner Comm. Model** | | | | |  | **Cross-Partner Comm. Model** | | | | |
| **Between-Person Results** |  | 1. | 2. | 3. | 4. |  |  | 1. | 2. | 3. | 4. |  |
| 1. Pos. Intercept |  | ̶ |  |  |  |  |  | ̶ |  |  |  |  |
| 2. Pos. Slope |  | -.06 | ̶ |  |  |  |  | -.30* | ̶ |  |  |  |
| 3. M. Sat. Intercept |  | .35* | -.27 | ̶ |  |  |  | .24* | .10 | ̶ |  |  |
| 4. M. Sat. Slope |  | .10 | .44 | -.03 | ̶ |  |  | .03 | -.39 | -.01 | ̶ |  |
| **Within-Person Results** |  | W1 | W2 | W3 | W4 | W5 |  | W1 | W2 | W3 | W4 | W5 |
| Cross-Lagged Paths |  |  |  |  |  |  |  |  |  |  |  |  |
| Pos._W-1_ → M. Sat. |  | ̶ | -.02^j^ | -.01^j^ | -.01^j^ | -.01^j^ |  | ̶ | **-.07*** | .02 | .05 | **.14*** |
| M. Sat. _W-1_ → Pos. |  | ̶ | -.02^k^ | -.02^k^ | -.02^k^ | -.03^k^ |  | ̶ | -.01^o^ | -.01^o^ | -.01^o^ | -.01^o^ |
| Autoregressive Paths |  |  |  |  |  |  |  |  |  |  |  |  |
| Positive |  | ̶ | .04^L^ | .03^L^ | .03^L^ | .03^L^ |  | ̶ | -.16* | .02 | .08* | .10* |
| M. Satisfaction |  | ̶ | .11*^m^ | .10*^m^ | .09*^m^ | .12*^m^ |  | ̶ | .11*^p^ | .11*^p^ | .09*^p^ | .12*^p^ |
| Concurrent Correlations |  |  |  |  |  |  |  |  |  |  |  |  |
| Pos. ↔ M. Sat. |  | .05*^n^ | .06*^n^ | .06*^n^ | .05*^n^ | .07*^n^ |  | -.02 | .02 | .05 | -.01 | .18* |

*Notes.* Standardized estimates. ^a - p^Corresponding coefficients are constrained to equality. Significant within-person cross-lagged paths are bolded for clarity. _W-1_Preceding Wave. F. = Female partner. M. = Male partner. Pos. = Positive communication. Sat. = Relationship satisfaction. W = Wave. The intercepts and slopes were regressed on relationship duration. Female relationship satisfaction and positive communication fit indices: χ^2^(39) = 42.482; RMSEA = .005 (.000, .013); CFI = .999; TLI = .999; SRMR = .020. Female relationship satisfaction and male positive communication fit indices: χ^2^(42) = 47.164; RMSEA = .006 (.000, .014); CFI = .999; TLI = .998; SRMR = .019. Male relationship satisfaction and positive communication fit indices: χ^2^(42) = 55.611; RMSEA = .010 (.000, .016); CFI = .996; TLI = .995; SRMR = .020. Male relationship satisfaction and female positive communication fit indices: χ^2^(32) = 46.633; RMSEA = .012 (.002, .018); CFI = .996; TLI = .993; SRMR = .022. **p* < .05.

Supplementary Table 16

*Standardized Bivariate ALT-SR Modeling Results for Negative Communication and Relationship Satisfaction in the Pairfam Study (Study 3; n = 3,405 couples)*

| **Female Satisfaction** |  | **Within-Partner Comm. Model** | | | | |  | **Cross-Partner Comm. Model** | | | | |
| --- | --- | --- | --- | --- | --- | --- | --- | --- | --- | --- | --- | --- |
| **Between-Person Results** |  | 1. | 2. | 3. | 4. |  |  | 1. | 2. | 3. | 4. |  |
| 1. Neg. Intercept |  | ̶ |  |  |  |  |  | ̶ |  |  |  |  |
| 2. Neg. Slope |  | -.28* | ̶ |  |  |  |  | .04 | ̶ |  |  |  |
| 3. F. Sat. Intercept |  | -.45* | .03 | ̶ |  |  |  | -.31* | -.25 | ̶ |  |  |
| 4. F. Sat. Slope |  | -.01 | -.38* | -.23 | ̶ |  |  | -.08 | -.11 | -.30* | ̶ |  |
| **Within-Person Results** |  | W1 | W2 | W3 | W4 | W5 |  | W1 | W2 | W3 | W4 | W5 |
| Cross-Lagged Paths |  |  |  |  |  |  |  |  |  |  |  |  |
| Neg._W-1_ → F. Sat. |  | ̶ | -.03^a^ | -.03^a^ | -.03^a^ | -.03^a^ |  | ̶ | -.04^e^ | -.04^e^ | -.04^e^ | -.04^e^ |
| F. Sat. _W-1_ → Neg. |  | ̶ | **-.09*** | .03 | -.03 | .07 |  | ̶ | **-.15*** | .00 | .04 | -.03 |
| Autoregressive Paths |  |  |  |  |  |  |  |  |  |  |  |  |
| Negative |  | ̶ | .05^b^ | .05^b^ | .06^b^ | .05^b^ |  | ̶ | .03^f^ | .03^f^ | .03^f^ | .02^f^ |
| F. Satisfaction |  | ̶ | .05*^c^ | .06*^c^ | .06*^c^ | .06*^c^ |  | ̶ | .04^g^ | .04^g^ | .05^g^ | .05^g^ |
| Concurrent Correlations |  |  |  |  |  |  |  |  |  |  |  |  |
| Neg. ↔ F. Sat. |  | -.17*^d^ | -.17*^d^ | -.18*^d^ | -.19*^d^ | -.19*^d^ |  | -.15* | -.14* | -.11* | -.08 | -.19* |
|  |  |  |  |  |  |  |  |  |  |  |  |  |
| **Male Satisfaction** |  | **Within-Partner Comm. Model** | | | | |  | **Cross-Partner Comm. Model** | | | | |
| **Between-Person Results** |  | 1. | 2. | 3. | 4. |  |  | 1. | 2. | 3. | 4. |  |
| 1. Neg. Intercept |  | ̶ |  |  |  |  |  | ̶ |  |  |  |  |
| 2. Neg. Slope |  | .03 | ̶ |  |  |  |  | -.32* | ̶ |  |  |  |
| 3. M. Sat. Intercept |  | -.47* | .08 | ̶ |  |  |  | -.32* | .03 | ̶ |  |  |
| 4. M. Sat. Slope |  | -.14 | -.72 | .13 | ̶ |  |  | -.07 | -.23 | .04 | ̶ |  |
| **Within-Person Results** |  | W1 | W2 | W3 | W4 | W5 |  | W1 | W2 | W3 | W4 | W5 |
| Cross-Lagged Paths |  |  |  |  |  |  |  |  |  |  |  |  |
| Neg._W-1_ → M. Sat. |  | ̶ | -.04^h^ | -.04^h^ | -.03^h^ | -.03^h^ |  | ̶ | **-.05***^L^ | **-.05***^L^ | **-.04***^L^ | **-.04***^L^ |
| M. Sat. _W-1_ → Neg. |  | ̶ | -.01^i^ | -.01^i^ | -.01^i^ | -.01^i^ |  | ̶ | -.02^m^ | -.02^m^ | -.02^m^ | -.02^m^ |
| Autoregressive Paths |  |  |  |  |  |  |  |  |  |  |  |  |
| Negative |  | ̶ | .04^j^ | .04^j^ | .04^j^ | .03^j^ |  | ̶ | .04^n^ | .04^n^ | .05^n^ | .04^n^ |
| M. Satisfaction |  | ̶ | .11*^k^ | .10*^k^ | .09*^k^ | .12*^k^ |  | ̶ | .11*^o^ | .10*^o^ | .09*^o^ | .12*^o^ |
| Concurrent Correlations |  |  |  |  |  |  |  |  |  |  |  |  |
| Neg. ↔ M. Sat. |  | -.12* | -.15* | -.15* | -.15* | -.25* |  | -.11*^p^ | -.12*^p^ | -.12*^p^ | -.11*^p^ | -.12*^p^ |

*Notes.* Standardized estimates. ^a - p^Corresponding coefficients are constrained to equality. Significant within-person cross-lagged paths are bolded for clarity. _W-1_Preceding Wave. F. = Female partner. M. = Male partner. Pos. = Positive communication. Sat. = Relationship satisfaction. W = Wave. The intercepts and slopes were regressed on relationship duration. Female relationship satisfaction and negative communication fit indices: χ^2^(39) = 42.417; RMSEA = .005 (.000, .013); CFI = 1.000; TLI = .999; SRMR = .022. Female relationship satisfaction and male negative communication fit indices: χ^2^(35) = 70.243; RMSEA = .017 (.011, .023); CFI = .994; TLI = .991; SRMR = .019. Male relationship satisfaction and negative communication fit indices: χ^2^(38) = 60.900; RMSEA = .013 (.006, .019); CFI = .996; TLI = .995; SRMR = .021. Male relationship satisfaction and female negative communication fit indices: χ^2^(42) = 75.087; RMSEA = .015 (.009, .021); CFI = .995; TLI = .994; SRMR = .024. **p* < .05.

**5. Between-Person Associations**

As discussed in the manuscript, the autoregressive latent trajectory models with structured residuals (ALT-SR; Curran et al., 2014) include the cross-sectional and longitudinal between-person associations between communication and relationship satisfaction. The main manuscript focuses on the within-person findings, but we discuss and present the between-person findings in this supplement. Cross-sectionally at the between-person level, the ALT-SR model considers whether individuals with more negative communication and less positive communication at the outset of the study have concurrently lower relationship satisfaction than individuals with more adaptive communication.

Two longitudinal between-person associations are tested: are initial levels of communication and relationship satisfaction associated with the rate of change in the other construct over time (intercept/slope associations), and are the rates of change in satisfaction associated with the rates of change in communication (slope/slope association). These between-person longitudinal analyses test whether individuals with higher initial negative communication and/or lower positive communication experience greater declines in satisfaction over time relative to individuals with more adaptive communication, and whether individuals with higher initial satisfaction experience better communication trajectories over time relative to individuals with lower initial satisfaction. Such questions are similar to those addressed in widely-used growth curve approaches testing between-couple differences in within-couple trajectories (e.g., Bradbury & Karney, 1997). Additionally, the slope-to-slope associations test whether those individuals with greater increases in negative communication and/or greater decreases in positive communication experience greater decreases in relationship satisfaction compared to individuals with smaller increases in negative communication and/or decreases in positive communication. Covariances among the growth parameters in the ALT-SR models (intercepts and slopes) test the between-person cross-sectional (e.g., intercept to intercept) and longitudinal (e.g., intercept to slope, and slope to slope) associations among communication and relationship satisfaction (see the dashed dotted lines in Figure 1). The latent intercept reflects the between-person difference in the initial levels of each construct and the slope reflects the between-person difference in intraindividual change trajectories (e.g., some people will increase, others decrease, and some remain stable).

**Study 1 (RDS): Between-Person Bivariate ALT-SR Modeling Results**

***Negative Communication and Relationship Satisfaction***

The between-person estimates are contained in Supplementary Table 8. Male and female partners’ negative communication intercepts were negatively correlated with their own and their partner’s relationship satisfaction intercepts. These cross-sectional findings mean that individuals with more initial negative communication tended to also have lower initial relationship satisfaction (as did their partners) compared to those with less initial negative communication. The negative communication intercept was also negatively correlated with each partner’s own relationship satisfaction slope, and female partners’ negative communication intercept was negatively associated with their male partner’s relationship satisfaction slope. These longitudinal findings indicate that individuals who reported higher initial negative communication had greater declines in their own relationship satisfaction over time compared to those with less initial negative communication, and male partners experienced greater declines in relationship satisfaction when their female partner reported more frequent initial negative communication compared to couples where female partners reported less frequent negative communication at the outset. Since the negative communication slope variances were set to zero, it was not possible to examine associations between the relationship satisfaction intercept and negative communication slope nor any slope-to-slope associations. Overall, these findings are similar to previous work examining how between-person differences in communication predict how couples’ relationship trajectories unfold (e.g., Johnson et al., 2005): Those who reported more negative couple communication at the outset of the study were also less satisfied at the outset of the study, as were their partners, and their own satisfaction deteriorated more over time compared to individuals who reported less negative communication.

**Study 2 (PASEZ): Between-Person Bivariate ALT-SR Modeling Results**

***Observed Positive Communication and Relationship Satisfaction***

The between-person estimates for observed positive communication and relationship satisfaction are presented in Supplementary Table 9. Higher initial observed positive communication for male and female partners was associated with higher initial female partner relationship satisfaction compared to couples with less initial positive communication (a cross-sectional intercept-to-intercept association). Additionally, higher female partner initial observed positive communication was associated with a smaller decrease in male partner relationship satisfaction over time compared to female partners with less initial observed positive communication (a longitudinal intercept-to-slope association), but was not associated with decreases in their own relationship satisfaction. Male partner initial observed positive communication was not associated with their own satisfaction intercept or slope, and was not associated with their female partner’s satisfaction slope. No associations were computed with the female partner positive communication slope because its trajectory was best characterized by a fixed slope model where the variance was fixed to zero.

***Observed Negative Communication and Relationship Satisfaction***

The between-person estimates for observed negative communication and relationship satisfaction are shown in Supplementary Table 10. Male and female partners’ observed negative communication intercepts were negatively correlated with their own and their partner’s relationship satisfaction intercepts (cross-sectional associations). Male and female partners with higher initial observed negative communication were initially less satisfied, as were their partners, compared to participants with less initial observed negative communication. There were no significant associations between observed negative communication intercepts and satisfaction slopes (longitudinal between-person associations). No negative communication slope associations were examined because the variances of these slopes were set to zero.

***Self-Reported Positive Communication and Relationship Satisfaction***

The between-person estimates for self-reported positive communication are reported in Supplementary Table 11. Female partners’ positive communication intercept was positively associated with their own relationship satisfaction intercept: female partners who reported higher initial positive communication were concurrently more satisfied than those who reported lower initial positive communication. Additionally, initial male partner positive communication was positively associated with his own and his partner’s relationship satisfaction intercepts. Female partners with higher initial relationship satisfaction also had greater increases in self-reported positive communication over time compared to those with lower initial satisfaction, but their male partners reported greater decreases in positive communication (longitudinal intercept-to-slope associations). There were no significant between-person associations between self-reported positive communication intercepts and relationship satisfaction slopes nor were the slopes of relationship satisfaction or positive communication significantly associated.

***Self-Report Negative Communication and Relationship Satisfaction***

The between-person estimates for self-report negative communication and relationship satisfaction are reported in Supplementary Table 12. The negative communication intercepts were negatively associated with one’s own and the partner’s relationship satisfaction intercepts: individuals who reported higher initial negative communication had lower initial relationship satisfaction for themselves and their partners compared to those with less initial self-reported negative communication. Longitudinally, negative communication intercepts were not significantly associated with satisfaction slopes. Male partners with higher initial relationship satisfaction had less of a decrease in their own and their partner’s self-reported negative communication compared to male partners with lower initial satisfaction (between-person longitudinal intercept-to-slope associations). One significant slope-to-slope association was also evident: a greater decrease in male partner’s relationship satisfaction was associated with a smaller decrease in female partner self-reported negative communication.

***Discussion of Between-Person Results with PASEZ Data***

The between-person intercept-to-intercept associations were robust for both measurement strategies. Couples with higher initial positive communication tended to have female partners who were also more satisfied initially compared to those with less initial positive communication. Additionally, higher initial negative communication was associated with less initial satisfaction for one’s self and one’s partner compared to those with less initial negative communication. Between-person intercept-to-slope associations were less robust: the only significant association between communication intercepts and satisfaction slopes was for female’s observed positive communication being associated with a more positive satisfaction slope for their male partners. There were a few associations between the relationship satisfaction intercepts and self-reported communication slopes: higher initial relationship satisfaction for both partners was associated with increases in self-reported positive communication for female partners and higher initial positive communication for female partners was associated with a decrease in male partner self-reported positive communication. Higher initial relationship satisfaction for either partner was associated with a more gradual decrease in male partner self-reported negative communication.

**Study 3 (Pairfam): Between-Person Bivariate ALT-SR Modeling Results**

***Positive Communication and Relationship Satisfaction***

The between-person estimates for the bivariate positive communication and relationship satisfaction models are reported in Supplementary Table 13. Across the models, one cross-construct association was consistent. For male and female partners, the positive communication intercept positively covaried with their own and their partner’s relationship satisfaction intercept in each model, indicating that individuals reporting more positive communication at the start of the study concurrently reported more satisfying relationships (as did their partners) relative to individuals reporting less positive communication at the start of the study. There were no longitudinal between-person associations, as positive communication intercepts were not associated with satisfaction slopes nor were satisfaction intercepts associated with positive communication slopes. No slope-to-slope associations were detected either.

***Negative Communication and Relationship Satisfaction***

The between-person estimates for negative communication and relationship satisfaction are contained in Supplementary Table 14. One cross-sectional association was consistent again. For male and female partners, the negative communication intercept negatively covaried with their own and their partner’s relationship satisfaction intercept in each model, indicating that individuals with more negative communication at Wave 1 had lower initial relationship satisfaction (as did their partners) compared to individuals with less negative communication. In the female partner relationship satisfaction/negative communication model only, the slopes of relationship satisfaction and negative communication were negatively associated (a longitudinal slope-to slope-association). This pattern indicates that female partners with a greater increase in their own negative communication had a greater decrease in relationship satisfaction compared to female partners with a smaller increase in negative communication. No other between-person longitudinal associations were evident, as negative communication intercepts were not significantly associated with satisfaction slopes, nor were satisfaction intercepts significantly associated with negative communication slopes.

***Discussion of Between-Person Results with Pairfam Data***

As in the analyses for the other studies already reported, the most robust cross-construct associations were the between-person cross-sectional intercept-to-intercept links: those who reported more frequent positive communication and less frequent negative communication at the outset of the study tended to also report higher initial satisfaction, as did their partners, compared to those who reported less frequent positive and more frequent negative communication. Communication intercepts were not significantly associated with satisfaction slopes, however.

Supplementary Table 17

*Summary of Between-Person Associations for Positive and Negative Communication and Relationship Satisfaction Across Studies*

| **Positive Communication: Between-Person Results** | | | | | | | | | | |
| --- | --- | --- | --- | --- | --- | --- | --- | --- | --- | --- |
|  |  | Within-Partner Comm. Model | | | |  | Cross-Partner Comm. Model | | | |
|  |  | Int. Sat. ↔  Int. Pos. | Int. Sat. ↔  Slp. Pos. | Int. Pos. ↔ Slp. Sat. | Slp. Pos. ↔ Slp. Sat. |  | Int. Sat. ↔  Int. Pos. | Int. Sat. ↔  Slp. Pos. | Int. Pos. ↔ Slp. Sat. | Slp. Pos. ↔ Slp. Sat. |
| Results for Female Satisfaction |  |  |  |  |  |  |  |  |  |  |
| Study 2: Obs. Pos. ↔ F. Sat. |  | **.23*** | ̶ | .15 | ̶ |  | **.17*** | .15 | .10 | .00 |
| Study 2: S. R. Pos. ↔ F. Sat. |  | **.24*** | **.24*** | .07 | .24 |  | **.41*** | **-.31*** | .04 | .44 |
| Study 3: Pos. ↔ F. Sat. |  | **.22*** | .06 | .09 | .08 |  | **.25*** | -.08 | -.02 | .18 |
| Results for Male Satisfaction |  |  | | | |  |  | | | |
| Study 2: Obs. Pos. ↔ M. Sat. |  | .13 | -.05 | .05 | .24 |  | -.04 | ̶ | **.44*** | ̶ |
| Study 2: S. R. Pos. ↔ M. Sat. |  | **.44*** | -.18 | .12 | .13 |  | .10 | **.25*** | .04 | .03 |
| Study 3: Pos. ↔ M. Sat. |  | **.35*** | -.27 | .10 | .44 |  | **.24*** | .10 | .03 | -.39 |
| **Negative Communication: Between-Person Results** | | | | | | | | | | |
|  |  | Within-Partner Comm. Model | | | |  | Cross-Partner Comm. Model | | | |
|  |  | Int. Sat. ↔  Int. Neg. | Int. Sat. ↔  Slp. Neg. | Int. Neg. ↔ Slp. Sat. | Slp. Neg. ↔ Slp. Sat. |  | Int. Sat. ↔  Int. Neg. | Int. Sat. ↔  Slp. Neg. | Int. Neg. ↔ Slp. Sat. | Slp. Neg. ↔ Slp. Sat. |
| Results for Female Satisfaction |  |  |  |  |  |  |  |  |  |  |
| Study 1: Neg. ↔ F. Sat. |  | **-.68*** | ̶ | **-.46*** | ̶ |  | **-.54*** | ̶ | -.19 | ̶ |
| Study 2: Obs. Neg. ↔ F. Sat. |  | **-.41*** | ̶ | -.23 | ̶ |  | **-.36*** | ̶ | -.02 | ̶ |
| Study 2: S. R. Neg. ↔ F. Sat. |  | **-.46*** | .03 | -.12 | -.18 |  | **-.41*** | .11 | -.20 | .37 |
| Study 3: Neg. ↔ F. Sat. |  | **-.44*** | .03 | -.01 | **-.38*** |  | **-.31*** | -.25 | -.08 | -.11 |
| Results for Male Satisfaction |  |  | | | |  |  | | | |
| Study 1: Neg. ↔ M. Sat. |  | **-.66*** | ̶ | **-.41*** | ̶ |  | **-.47*** | ̶ | **-.42*** | ̶ |
| Study 2: Obs. Neg. ↔ M. Sat. |  | **-.20*** | ̶ | .01 | ̶ |  | **-.25*** | ̶ | -.20 | ̶ |
| Study 2: S. R. Neg. ↔ M. Sat. |  | **-.48*** | **.31*** | -.06 | -.11 |  | **-.32*** | **.23*** | -.10 | **-.44*** |
| Study 3: Neg. ↔ M. Sat. |  | **-.47*** | .08 | -.14 | -.72 |  | **-.31*** | -.25 | -.08 | -.11 |

*Notes.* Standardized estimates. Significant effects shown in bold for emphasis. Slope variances were fixed to 0 in some models, so no correlations were estimated. Positive communication was not assessed in Study 1. F. = Female partner. M. = Male partner. Pos. = Positive communication. Neg. = Negative communication. Sat. = Relationship satisfaction. W = Wave. Obs. = Observed. S. R. = Self-reported. Int. = Intercept. Slp. = Slope.

**6. Dyadic ALT-SR Models**

This supplement also includes results from an alternate analytic strategy, fully dyadic ALT-SR models that simultaneously model associations between men and women’s relationship satisfaction and both partners’ communication. We opted to present results from bivariate models in the manuscript because those models are less complex and therefore provide the most statistical power to detect any within-person communication/satisfaction associations that may be present in the data. As a robustness check (Duncan et al., 2014), however, we also computed fully dyadic models for each study and the pattern of results was largely consistent in each modeling strategy. When discrepancies in patterns of significance emerged across the two sets of analyses, the coefficients were not substantively different from each other and these discrepancies did not reflect any consistent pattern of effect. Tables containing results for the fully dyadic ALT-SR models and figures comparing the coefficients obtained in each modeling approach are reported below for each study.

**Study 1 (RDS): Dyadic ALT-SR Modeling Results**

***Negative Communication and Relationship Satisfaction***

The dyadic ALT-SR model results that simultaneously modeled both partners’ relationship satisfaction and negative communication in the RDS are shown in Supplementary Table 16. Overall, the pattern of results for the lagged effects was largely similar across models (see Supplementary Figures 1 and 2 for comparisons of the coefficients), with identical patterns of results for the within-person, within-partner negative communication to relationship satisfaction paths. This analysis also yielded cross-partner negative communication to relationship satisfaction paths that were not significant in the bivariate models, with one robust pattern: Higher than typical male partner negative communication predicted an intraindividual reduction in female partner relationship satisfaction at all waves. Additionally, an upward deviation in female partner negative communication at Wave 2 predicted a within-person decrease in male partner relationship satisfaction only at Wave 3. Lastly, only one of the two within-partner relationship satisfaction to negative communication paths remained significant in this model: higher than normal Wave 4 male partner relationship satisfaction predicted a within-person decrease in his own negative communication at Wave 5.

Supplementary Table 18

*Standardized Dyadic ALT-SR Modeling Results for Male and Female Partner Negative Communication and Relationship Satisfaction in the RDS Study (n = 316 couples)*

| **Between-Person Results** | 1. | 2. | 3. | 4. | 5. | 6. | 7. | 8. |
| --- | --- | --- | --- | --- | --- | --- | --- | --- |
| 1. F. Neg. Intercept | ̶ |  |  |  |  |  |  |  |
| 2. F. Neg. Slope | ̶ | ̶ |  |  |  |  |  |  |
| 3. F. Sat. Intercept | -.69* | ̶ | ̶ |  |  |  |  |  |
| 4. F. Sat. Slope | -.36* | ̶ | .23 | ̶ |  |  |  |  |
| 5. M. Neg. Intercept | .65* | ̶ | -.55* | -.02 | ̶ |  |  |  |
| 6. M. Neg. Slope | ̶ | ̶ | ̶ | ̶ | ̶ | ̶ |  |  |
| 7. M. Sat. Intercept | -.45* | ̶ | .67* | .01 | -.62* | ̶ | ̶ |  |
| 8. M. Sat. Slope | ̶ | ̶ | ̶ | ̶ | ̶ | ̶ | ̶ | ̶ |
| **Within-Person Results** | W1 | W2 | W3 | W4 | W5 |  |  |  |
| Cross-Lagged Paths |  |  |  |  |  |  |  |  |
| Within-Partner Cross-Domain |  |  |  |  |  |  |  |  |
| F. Neg. _W-1_ → F. Sat. | ̶ | **-.12*^a^** | **-.10*^a^** | **-.12*^a^** | **-.09*^a^** |  |  |  |
| F. Sat. _W-1_ → F. Neg. | ̶ | -.11^b^ | -.11^b^ | -.10^b^ | -.09^b^ |  |  |  |
| M. Neg. _W-1_ → M. Sat. | ̶ | -.10 | .00 | -.16 | **-.20*** |  |  |  |
| M. Sat. _W-1_ → M. Neg. | ̶ | -.04 | .05 | .09 | **-.33*** |  |  |  |
| Cross-Partner Cross-Domain |  |  |  |  |  |  |  |  |
| F. Neg. _W-1_ → M. Sat. | ̶ | -.05^c^ | -.04^c^ | -.04^c^ | -.04^c^ |  |  |  |
| F. Sat. _W-1_ → M Neg. | ̶ | .15 | **-.28*** | -.07 | -.08 |  |  |  |
| M. Neg. _W-1_ → F. Sat. | ̶ | **-.17*^d^** | **-.14*^d^** | **-.19*^d^** | **-.11*^d^** |  |  |  |
| M. Sat. _W-1_ → F Neg. | ̶ | -.02^e^ | -.02^e^ | -.02^e^ | -.02^e^ |  |  |  |
| Cross-Partner Within-Domain |  |  |  |  |  |  |  |  |
| F. Neg. _W-1_ → M. Neg. | ̶ | .11^f^ | .08^f^ | .09^f^ | .08^f^ |  |  |  |
| F. Sat. _W-1_ → M. Sat. | ̶ | .02 | .10 | .10 | **.34*** |  |  |  |
| M. Neg. _W-1_ → F. Neg. | ̶ | **.21*^g^** | **.18*^g^** | **.18*^g^** | **.17***^g^ |  |  |  |
| M. Sat. _W-1_ → F. Sat. | ̶ | .04 | .00 | .17 | **.41*** |  |  |  |
| Autoregressive Paths |  |  |  |  |  |  |  |  |
| F. Negative | ̶ | .08^h^ | .06^h^ | .06^h^ | .07^h^ |  |  |  |
| F. Satisfaction | ̶ | .09^i^ | .09^i^ | .11^i^ | .06^i^ |  |  |  |
| M. Negative | ̶ | .25*^j^ | .19*^j^ | .23*^j^ | .17*^j^ |  |  |  |
| M. Satisfaction | ̶ | .27*^k^ | .40*^k^ | .33*^k^ | .33*^k^ |  |  |  |
| Within-Time Correlations |  |  |  |  |  |  |  |  |
| F. Neg.↔ F. Sat. | -.41*^L^ | -.52*^L^ | -.49*^L^ | -.59*^L^ | -.49*^L^ |  |  |  |
| F. Neg.↔ M. Neg. | .49* | .26* | .30* | .36* | .28* |  |  |  |
| F. Neg.↔ M. Sat. | -.27*^m^ | -.27*^m^ | -.30*^m^ | -.25*^m^ | -.32*^m^ |  |  |  |
| F. Sat. ↔ M. Neg. | -.23*^n^ | -.29*^n^ | -.26*^n^ | -.37*^n^ | -.26*^n^ |  |  |  |
| F. Sat. ↔ M. Sat. | .12 | .42* | .47* | .47* | .64* |  |  |  |
| M. Neg.↔ M. Sat. | -.41*^o^ | -.41*^o^ | -.44*^o^ | -.44*^o^ | -.48*^o^ |  |  |  |

*Notes.* Standardized estimates. ^a - o^Corresponding coefficients are constrained to equality. Significant cross-lagged paths are bolded for clarity. Negative communication and male partner relationship satisfaction slope variance was fixed to 0. _W-1_Preceding Wave. F. = Female partner. M. = Male partner. Neg. = Negative communication. Sat. = Relationship satisfaction. W = Wave. The intercepts and slope for male partner relationship satisfaction were regressed on relationship duration. Model fit indices: χ^2^(155) = 265.467; RMSEA = .047 (.038, .057); CFI = .972; TLI = .962; SRMR = .068. **p* < .05.

Supplementary Figure 1

*Comparison Between the Longitudinal Standardized Regression Coefficients from the RDS Negative Communication and Relationship Satisfaction Bivariate (see Table 1) and Dyadic Models (see Supplementary Table 2)*


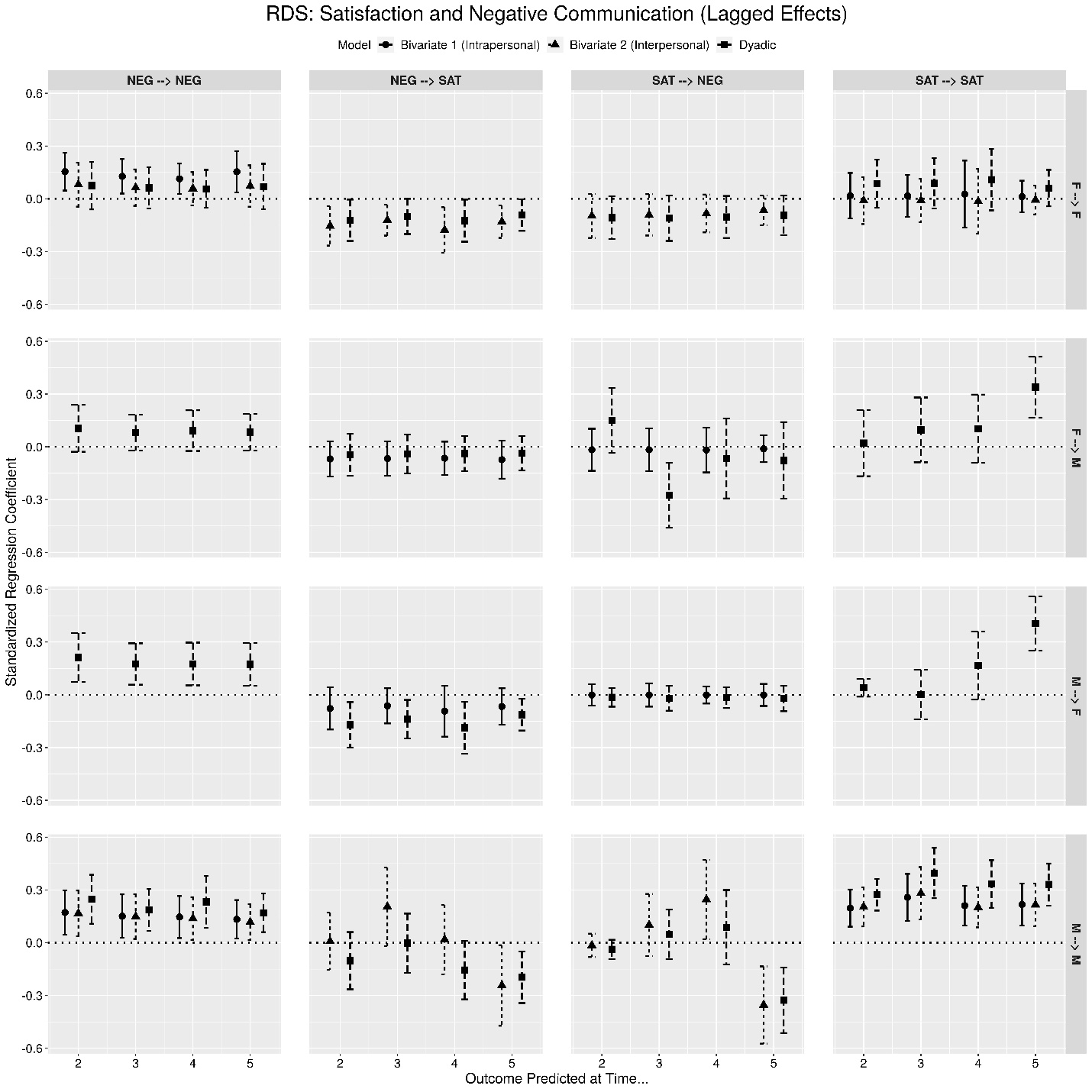


*Notes.* NEG = Negative communication. SAT = Relationship satisfaction. M = Male partner. F = Female partner. The arrow (-->) indicates the direction of the effect. The bivariate model 1 contains intrapersonal effects (e.g., effects of female partner satisfaction on female partner communication), whereas the bivariate model 2 contains interpersonal effects (e.g., effects of female partner satisfation on male partner communication).

Supplementary Figure 2

*Comparison Between the Witihin-Time Standardized Regression Coefficients from the RDS Negative Communication and Relationship Satisfaction Bivariate (see Table 1) and Dyadic Models (see Supplementary Table 2)*


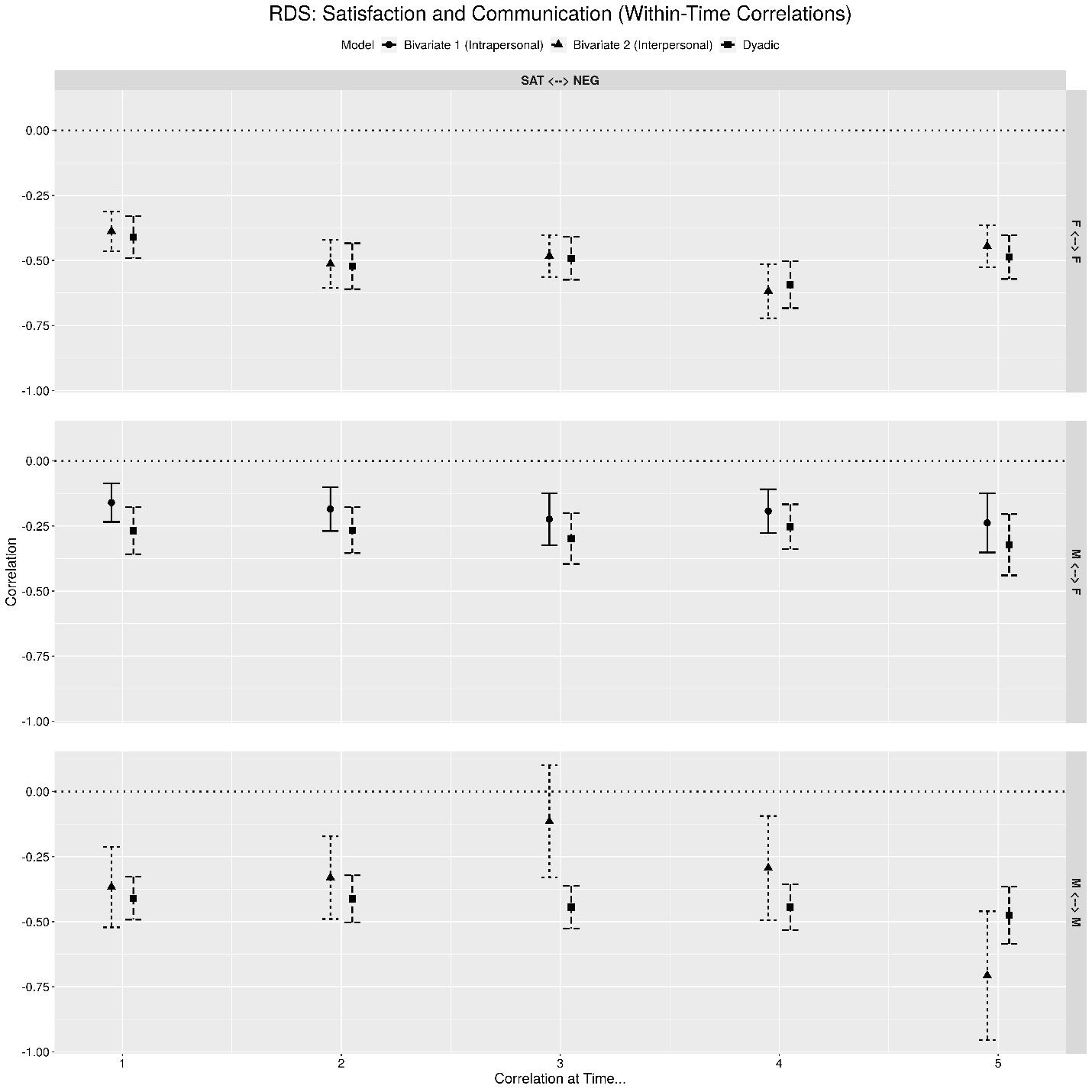


*Notes.* NEG = Negative communication. SAT = Relationship satisfaction. M = Male partner. F = Female partner. The bivariate model 1 contains intrapersonal effects (e.g., correlation between male partner satisfaction and male partner communication), whereas the bivariate model 2 contains interpersonal effects (e.g., correlations between female partner satisfation and male partner communication).

**Study 2 (PASEZ): Dyadic ALT-SR Modeling Results**

***Observed Positive Communication and Relationship Satisfaction***

The dyadic ALT-SR model results that simultaneously modeled both partners’ relationship satisfaction and observed positive communication with PASEZ data are shown in Supplementary Table 17. These results were largely consistent with those in the bivariate models (see Supplementary Figures 3 and 5 for comparisons of the coefficients): the between-person associations were more robust than the within-person links. Although the bivariate models revealed two of twenty cross-sectional within-person associations to be significant, these were not evident in the fully dyadic model. Likewise, two of thirty-two within-person cross-lagged paths were significant in the bivariate models, but no cross-construct cross-lagged associations were significant in the fully dyadic model. Overall, results from both analyses indicate that cross-sectional and lagged associations between observed positive communication and relationship satisfaction were mostly non-significant.

Supplementary Table 19

*Standardized Dyadic ALT-SR Modeling Results for Male and Female Partner Observed Positive Communication and Relationship Satisfaction in the PASEZ Study (n = 365 couples)*

| **Between-Person Results** | 1. | 2. | 3. | 4. | 5. | 6. | 7. | 8. |
| --- | --- | --- | --- | --- | --- | --- | --- | --- |
| 1. F. Pos. Intercept | ̶ |  |  |  |  |  |  |  |
| 2. F. Pos. Slope | ̶ | ̶ |  |  |  |  |  |  |
| 3. F. Sat. Intercept | .25* | ̶ | ̶ |  |  |  |  |  |
| 4. F. Sat. Slope | .13 | ̶ | .32 | ̶ |  |  |  |  |
| 5. M. Pos. Intercept | .60* | ̶ | .16 | .14 | ̶ |  |  |  |
| 6. M. Pos. Slope | -.56 | ̶ | .03 | -.04 | -.75* | ̶ |  |  |
| 7. M. Sat. Intercept | .11 | ̶ | .69* | .16 | .12 | .03 | ̶ |  |
| 8. M. Sat. Slope | .21 | ̶ | .12 | .74* | .04 | .23 | -.02 | ̶ |
| **Within-Person Results** | W1 | W2 | W3 | W4 | W5 |  |  |  |
| Cross-Lagged Paths |  |  |  |  |  |  |  |  |
| Within-Partner Cross-Domain |  |  |  |  |  |  |  |  |
| F. Pos._W-1_ → F. Sat. | ̶ | -.05^a^ | -.03^a^ | -.03^a^ | -.03^a^ |  |  |  |
| F. Sat. _W-1_ → F. Pos. | ̶ | -.07^b^ | -.08^b^ | -.07^b^ | -.08^b^ |  |  |  |
| M. Pos. _W-1_ → M. Sat. | ̶ | .04^c^ | .03^c^ | .03^c^ | .05^c^ |  |  |  |
| M. Sat. _W-1_ → M. Pos. | ̶ | -.05^b^ | -.05^b^ | -.04^b^ | -.04^b^ |  |  |  |
| Cross-Partner Cross-Domain |  |  |  |  |  |  |  |  |
| F. Pos. _W-1_ → M. Sat. | ̶ | -.01^e^ | -.01^e^ | -.01^e^ | -.01^e^ |  |  |  |
| F. Sat. _W-1_ → M Pos. | ̶ | .03^f^ | .04^f^ | .03^f^ | .03^f^ |  |  |  |
| M. Pos. _W-1_ → F. Sat. | ̶ | .01^g^ | .01^g^ | .01^g^ | .01^g^ |  |  |  |
| M. Sat. _W-1_ → F Pos. | ̶ | .02^h^ | .02^h^ | .02^h^ | .02^h^ |  |  |  |
| Cross-Partner Within-Domain |  |  |  |  |  |  |  |  |
| F. Pos. _W-1_ → M. Pos. | ̶ | .04^i^ | .03^i^ | .02^i^ | .03^i^ |  |  |  |
| F. Sat. _W-1_ → M. Sat. | ̶ | .01^j^ | .01^j^ | .01^j^ | .01^j^ |  |  |  |
| M. Pos. _W-1_ → F. Pos. | ̶ | -.01^k^ | -.01^k^ | -.00^k^ | -.01^k^ |  |  |  |
| M. Sat. _W-1_ → F. Sat. | ̶ | -.04^L^ | -.04^L^ | -.04^L^ | -.04^L^ |  |  |  |
| Autoregressive Paths |  |  |  |  |  |  |  |  |
| F. Positive | ̶ | .23*^m^ | .16*^m^ | .13*^m^ | .17*^m^ |  |  |  |
| F. Satisfaction | ̶ | .04^n^ | .05^n^ | .05^n^ | .05^n^ |  |  |  |
| M. Positive | ̶ | .01^o^ | .01^o^ | .00^o^ | .01^o^ |  |  |  |
| M. Satisfaction | ̶ | .03^p^ | .03^p^ | .03^p^ | .03^p^ |  |  |  |
| Within-Time Correlations |  |  |  |  |  |  |  |  |
| F. Pos. ↔ F. Sat. | -.03^q^ | -.04^q^ | -.04^q^ | -.04^q^ | -.04^q^ |  |  |  |
| F. Pos. ↔ M. Pos. | .15*^r^ | .28*^r^ | .32*^r^ | .18*^r^ | .21*^r^ |  |  |  |
| F. Pos. ↔ M. Sat. | .04^s^ | .07^s^ | .06^s^ | .05^s^ | .07^s^ |  |  |  |
| F. Sat. ↔ M. Pos. | .02^t^ | .02^t^ | .02^t^ | .01^t^ | .01^t^ |  |  |  |
| F. Sat. ↔ M. Sat. | .37*^u^ | .34*^u^ | .30*^u^ | .32*^u^ | .31*^u^ |  |  |  |
| M. Pos. ↔ M. Sat. | .03^v^ | .04^v^ | .04^v^ | .03^v^ | .03^v^ |  |  |  |

*Notes.* Standardized estimates. ^a - v^Corresponding coefficients are constrained to equality. The female partner positive communication slope variance was fixed to 0. _W-1_Preceding Wave. F. = Female partner. M. = Male partner. Pos. = Positive communication. Sat. = Relationship satisfaction. W = Wave. The intercepts and slopes were regressed on relationship duration. Model fit indices: χ^2^(164) = 173.492; RMSEA = .013 (.000, .027); CFI = .996; TLI = .995; SRMR = .060. **p* < .05.

**Study 2 (PASEZ): Dyadic ALT-SR Modeling Results**

***Observed Negative Communication and Relationship Satisfaction***

The dyadic ALT-SR model results that simultaneously modeled both partners’ relationship satisfaction and observed negative communication with PASEZ data are shown in Supplementary Table 18. Compared to the bivariate models reported in the main document, the between-person associations were similarly robust in the fully dyadic model, but some significant within-person associations were not consistent with those in the bivariate models (see Supplementary Figures 4 and 5 for comparisons of the coefficients). Cross-sectionally, the association between upward deviations in female partners’ observed negative communication and their own and their partner’s relationship satisfaction was evident in both models. Additionally, unlike in the bivariate model, the fully dyadic model also found upward deviations in male partners’ observed negative communication were concurrently linked with less than average relationship satisfaction for themselves. In terms of within-person cross-lagged associations, the bivariate models revealed higher than average relationship satisfaction in male partners consistently predicted an intraindividual reduction in female partners’ future observed negative communication, but these links were not significant in the fully dyadic models. Rather, in the dyadic models, upward deviations in female partner observed negative communication consistently predicted an intraindividual reduction in her own and her partner’s future relationship satisfaction; these links were not significant in the bivariate models.

Supplementary Table 20

*Standardized Dyadic ALT-SR Modeling Results for Male and Female Partner Observed Negative Communication and Relationship Satisfaction in the PASEZ Study (n = 365 couples)*

| **Between-Person Results** | 1. | 2. | 3. | 4. | 5. | 6. | 7. | 8. |
| --- | --- | --- | --- | --- | --- | --- | --- | --- |
| 1. F. Neg. Intercept | ̶ |  |  |  |  |  |  |  |
| 2. F. Neg. Slope | ̶ | ̶ |  |  |  |  |  |  |
| 3. F. Sat. Intercept | -.44* | ̶ | ̶ |  |  |  |  |  |
| 4. F. Sat. Slope | -.31* | ̶ | .28 | ̶ |  |  |  |  |
| 5. M. Neg. Intercept | .65* | ̶ | -.35* | -.09 | ̶ |  |  |  |
| 6. M. Neg. Slope | ̶ | ̶ | ̶ | ̶ | ̶ | ̶ |  |  |
| 7. M. Sat. Intercept | -.27* | ̶ | .69* | .11 | -.18* | ̶ | ̶ |  |
| 8. M. Sat. Slope | -.22 | ̶ | .06 | .73* | .01 | ̶ | -.09 | ̶ |
| **Within-Person Results** | W1 | W2 | W3 | W4 | W5 |  |  |  |
| Cross-Lagged Paths |  |  |  |  |  |  |  |  |
| Within-Partner Cross-Domain |  |  |  |  |  |  |  |  |
| F. Neg. _W-1_ → F. Sat. | ̶ | **-.12*^a^** | **-.10*^a^** | **-.16*^a^** | **-.14*^a^** |  |  |  |
| F. Sat. _W-1_ → F. Neg. | ̶ | -.04^b^ | -.03^b^ | -.03^b^ | -.03^b^ |  |  |  |
| M. Neg. _W-1_ → M. Sat. | ̶ | -.02^c^ | -.02^c^ | -.03^c^ | -.04^c^ |  |  |  |
| M. Sat. _W-1_ → M. Neg. | ̶ | -.01^d^ | -.01^d^ | -.01^d^ | -.01^d^ |  |  |  |
| Cross-Partner Cross-Domain |  |  |  |  |  |  |  |  |
| F. Neg. _W-1_ → M. Sat. | ̶ | **-.12*^e^** | **-.11*^e^** | **-.17*^e^** | **-.18*^e^** |  |  |  |
| F. Sat. _W-1_ → M Neg. | ̶ | .02^f^ | .02^f^ | .02^f^ | .02^f^ |  |  |  |
| M. Neg. _W-1_ → F. Sat. | ̶ | .04^g^ | .04^g^ | .05^g^ | .05^g^ |  |  |  |
| M. Sat. _W-1_ → F Neg. | ̶ | -.11^h^ | -.07^h^ | -.08^h^ | -.07^h^ |  |  |  |
| Cross-Partner Within-Domain |  |  |  |  |  |  |  |  |
| F. Neg. _W-1_ → M. Neg. | ̶ | **.15*^i^** | **.12*^i^** | **.16*^i^** | **.15*^i^** |  |  |  |
| F. Sat. _W-1_ → M. Sat. | ̶ | .01^j^ | .01^j^ | .02^j^ | .02^j^ |  |  |  |
| M. Neg. _W-1_ → F. Neg. | ̶ | .05^k^ | .04^k^ | .05^k^ | .05^k^ |  |  |  |
| M. Sat. _W-1_ → F. Sat. | ̶ | -.06^L^ | -.05^L^ | -.06^L^ | -.05^L^ |  |  |  |
| Autoregressive Paths |  |  |  |  |  |  |  |  |
| F. Negative | ̶ | -.12 | .27* | .35* | .19* |  |  |  |
| F. Satisfaction | ̶ | .05^m^ | .05^m^ | .06^m^ | .05^m^ |  |  |  |
| M. Negative | ̶ | -.21* | .20* | .16* | .13 |  |  |  |
| M. Satisfaction | ̶ | -.01^n^ | -.01^n^ | -.01^n^ | -.01^n^ |  |  |  |
| Within-Time Correlations |  |  |  |  |  |  |  |  |
| F. Neg.↔ F. Sat. | -.24*^o^ | -.22*^o^ | -.14*^o^ | -.16*^o^ | -.13*^o^ |  |  |  |
| F. Neg.↔ M. Neg. | .38* | .59* | .61* | .56* | .67* |  |  |  |
| F. Neg.↔ M. Sat. | -.27*^p^ | -.29*^p^ | -.19*^p^ | -.22*^p^ | -.22*^p^ |  |  |  |
| F. Sat. ↔ M. Neg. | -.10^q^ | -.07^q^ | -.06^q^ | -.05^q^ | -.05^q^ |  |  |  |
| F. Sat. ↔ M. Sat. | .38*^r^ | .34*^r^ | .30*^r^ | .33*^r^ | .33*^r^ |  |  |  |
| M. Neg.↔ M. Sat. | -.12*^s^ | -.10*^s^ | -.08*^s^ | -.08*^s^ | -.08*^s^ |  |  |  |

*Notes.* Standardized estimates. ^a - s^Corresponding coefficients are constrained to equality. Significant cross-lagged paths are bolded for clarity. The negative communication slope variances for male and female partners were fixed to 0. _W-1_Preceding Wave. F. = Female partner. M. = Male partner. Neg. = Negative communication. Sat. = Relationship satisfaction. W = Wave. The intercepts and slopes were regressed on relationship duration. Model fit indices: χ^2^(161) = 202.009; RMSEA = .026 (.012, .037); CFI = .987; TLI = .983; SRMR = .059. **p* < .05.

**Study 2 (PASEZ): Dyadic ALT-SR Modeling Results**

***Self-Reported Positive Communication and Relationship Satisfaction***

Results from the fully dyadic ALT-SR model that simultaneously modeled both partners’ relationship satisfaction and self-reported positive communication (shown in Supplementary Table 19) were generally consistent with the bivariate models computed with PASEZ data (see Supplementary Figures 6 and 8 for comparisons of the coefficients), finding evidence for between-person effects for men and women but minimal evidence for within-person effects. In the fully dyadic model, one within-person cross-sectional link arose (none were significant in the bivariate models): within-time upward deviations in male partner relationship satisfaction were associated with a concurrent uptick in their self-reported positive communication.

Supplementary Table 21

*Standardized Dyadic ALT-SR Modeling Results for Male and Female Partner Self-Reported Positive Communication and Relationship Satisfaction in the PASEZ Study (n = 365 couples)*

| **Between-Person Results** | 1. | 2. | 3. | 4. | 5. | 6. | 7. | 8. |
| --- | --- | --- | --- | --- | --- | --- | --- | --- |
| 1. F. Pos. Intercept | ̶ |  |  |  |  |  |  |  |
| 2. F. Pos. Slope | -.33* | ̶ |  |  |  |  |  |  |
| 3. F. Sat. Intercept | .26* | .23* | ̶ |  |  |  |  |  |
| 4. F. Sat. Slope | .10 | .30 | .35 | ̶ |  |  |  |  |
| 5. M. Pos. Intercept | .21* | .15 | .44* | .06 | ̶ |  |  |  |
| 6. M. Pos. Slope | -.03 | -.03 | -.36* | .58 | -.10 | ̶ |  |  |
| 7. M. Sat. Intercept | .10 | .24* | .69* | .16 | .46* | -.18 | ̶ |  |
| 8. M. Sat. Slope | .02 | .10 | .08 | .80* | .12 | .20 | -.05 | ̶ |
| **Within-Person Results** | W1 | W2 | W3 | W4 | W5 |  |  |  |
| Cross-Lagged Paths |  |  |  |  |  |  |  |  |
| Within-Partner Cross-Domain |  |  |  |  |  |  |  |  |
| F. Pos._W-1_ → F. Sat. | ̶ | -.02^a^ | -.02^a^ | -.02^a^ | -.02^a^ |  |  |  |
| F. Sat. _W-1_ → F. Pos. | ̶ | -.04^b^ | -.04^b^ | -.04^b^ | -.05^b^ |  |  |  |
| M. Pos. _W-1_ → M. Sat. | ̶ | -.07^c^ | -.06^c^ | -.07^c^ | -.08^c^ |  |  |  |
| M. Sat. _W-1_ → M. Pos. | ̶ | .02^d^ | .02^d^ | .02^d^ | .02^d^ |  |  |  |
| Cross-Partner Cross-Domain |  |  |  |  |  |  |  |  |
| F. Pos. _W-1_ → M. Sat. | ̶ | .03^e^ | .04^e^ | .03^e^ | .05^e^ |  |  |  |
| F. Sat. _W-1_ → M Pos. | ̶ | -.01^f^ | -.01^f^ | -.01^f^ | -.01^f^ |  |  |  |
| M. Pos. _W-1_ → F. Sat. | ̶ | -.07^g^ | -.05^g^ | -.06^g^ | -.06^g^ |  |  |  |
| M. Sat. _W-1_ → F Pos. | ̶ | .02^h^ | .02^h^ | .02^h^ | .03^h^ |  |  |  |
| Cross-Partner Within-Domain |  |  |  |  |  |  |  |  |
| F. Pos. _W-1_ → M. Pos. | ̶ | .02^i^ | .03^i^ | .03^i^ | .03^i^ |  |  |  |
| F. Sat. _W-1_ → M. Sat. | ̶ | .01^j^ | .01^j^ | .01^j^ | .01^j^ |  |  |  |
| M. Pos. _W-1_ → F. Pos. | ̶ | .03^k^ | .03^k^ | .02^k^ | .03^k^ |  |  |  |
| M. Sat. _W-1_ → F. Sat. | ̶ | -.06^L^ | -.05^L^ | -.05^L^ | -.05^L^ |  |  |  |
| Autoregressive Paths |  |  |  |  |  |  |  |  |
| F. Positive | ̶ | .06^m^ | .10^m^ | .07^m^ | .11^m^ |  |  |  |
| F. Satisfaction | ̶ | .06^n^ | .06^n^ | .06^n^ | .06^n^ |  |  |  |
| M. Positive | ̶ | -.05^o^ | -.04^o^ | -.05^o^ | -.05^o^ |  |  |  |
| M. Satisfaction | ̶ | .02^p^ | .02^p^ | .02^p^ | .03^p^ |  |  |  |
| Within-Time Correlations |  |  |  |  |  |  |  |  |
| F. Pos. ↔ F. Sat. | .07^q^ | .04^q^ | .05^q^ | .04^q^ | .04^q^ |  |  |  |
| F. Pos. ↔ M. Pos. | .08^r^ | .06^r^ | .07^r^ | .05^r^ | .07^r^ |  |  |  |
| F. Pos. ↔ M. Sat. | .10^s^ | .07^s^ | .07^s^ | .06^s^ | .08^s^ |  |  |  |
| F. Sat. ↔ M. Pos. | -.02*^t^ | -.02*^t^ | -.01*^t^ | -.01*^t^ | -.01*^t^ |  |  |  |
| F. Sat. ↔ M. Sat. | .35*^u^ | .33*^u^ | .29*^u^ | .29*^u^ | .30*^u^ |  |  |  |
| M. Pos. ↔ M. Sat. | .06^v^ | .07*^v^ | .07*^v^ | .07*^v^ | .07*^v^ |  |  |  |

*Notes.* Standardized estimates. ^a - t^Corresponding coefficients are constrained to equality.

_W-1_Preceding Wave. F. = Female partner. M. = Male partner. Pos. = Positive communication. Sat. = Relationship satisfaction. W = Wave. The intercepts and slopes were regressed on relationship duration. Model fit indices: χ^2^(156) = 172.978; RMSEA = .017 (.000, .030); CFI = .995; TLI = .994; SRMR = .056. **p* < .05.

**Study 2 (PASEZ): Dyadic ALT-SR Modeling Results**

***Self-Reported Negative Communication and Relationship Satisfaction***

The dyadic ALT-SR model results that simultaneously modeled both partners’ relationship satisfaction and self-reported negative communication with PASEZ data are presented in Supplementary Table 20. Most key results were consistent across the models (see Supplementary Figures 7 and 8 for comparisons of the coefficients), such that an intraindividual increase in male partner self-reported negative communication consistently predicted a future intrapersonal reduction in his own and his partner’s relationship satisfaction at all four lags and higher than normal relationship satisfaction for male partners predicted an intraindividual decrease in male partner future self-reported negative communication. However, there were key differences as well. The consistent lagged female partner relationship satisfaction to male partner self-reported negative communication path in the bivariate model was no longer significant in the fully dyadic model, nor was the single within-person cross-lagged path from higher than average female partner satisfaction at Wave 1 predicting an intraindividual increase in male partner negative communication at Wave 2. There was also an additional within-time association only in the fully dyadic model between upward deviations in female partner self-reported negative communication and less than average relationship satisfaction for male partners; this was not significant in the bivariate model.

Supplementary Table 22

*Standardized Dyadic ALT-SR Modeling Results for Male and Female Partner Self-Reported Negative Communication and Relationship Satisfaction in the PASEZ Study (n = 365 couples)*

| **Between-Person Results** | 1. | 2. | 3. | 4. | 5. | 6. | 7. | 8. |
| --- | --- | --- | --- | --- | --- | --- | --- | --- |
| 1. F. Neg. Intercept | ̶ |  |  |  |  |  |  |  |
| 2. F. Neg. Slope | -.46* | ̶ |  |  |  |  |  |  |
| 3. F. Sat. Intercept | -.43* | -.06 | ̶ |  |  |  |  |  |
| 4. F. Sat. Slope | -.19 | .12 | .29 | ̶ |  |  |  |  |
| 5. M. Neg. Intercept | .47* | -.12 | -.42* | -.23 | ̶ |  |  |  |
| 6. M. Neg. Slope | -.21 | -.02 | .09 | .52 | -.40* | ̶ |  |  |
| 7. M. Sat. Intercept | -.31* | .24 | .68* | .16 | -.48* | .31* | ̶ |  |
| 8. M. Sat. Slope | -.15 | -.42 | .11 | .76* | -.10 | -.10 | -.05 | ̶ |
| **Within-Person Results** | W1 | W2 | W3 | W4 | W5 |  |  |  |
| Cross-Lagged Paths |  |  |  |  |  |  |  |  |
| Within-Partner Cross-Domain |  |  |  |  |  |  |  |  |
| F. Neg. _W-1_ → F. Sat. | ̶ | .01^a^ | .01^a^ | .01^a^ | .01^a^ |  |  |  |
| F. Sat. _W-1_ → F. Neg. | ̶ | -.09 | .04 | **.17*** | -.22 |  |  |  |
| M. Neg. _W-1_ → M. Sat. | ̶ | **-.16*^b^** | **-.11*^b^** | **-.14*^b^** | **-.12*^b^** |  |  |  |
| M. Sat. _W-1_ → M. Neg. | ̶ | **-.15*^c^** | **-.12*^c^** | **-.17*^c^** | **-.16*^c^** |  |  |  |
| Cross-Partner Cross-Domain |  |  |  |  |  |  |  |  |
| F. Neg. _W-1_ → M. Sat. | ̶ | .05^d^ | .06^d^ | .06^d^ | .05^d^ |  |  |  |
| F. Sat. _W-1_ → M Neg. | ̶ | -.07^e^ | -.07^e^ | -.09^e^ | -.09^e^ |  |  |  |
| M. Neg. _W-1_ → F. Sat. | ̶ | **-.19*^f^** | **-.14*^f^** | **-.17*^f^** | **-.12*^f^** |  |  |  |
| M. Sat. _W-1_ → F Neg. | ̶ | .02^g^ | .01^g^ | .02^g^ | .02^g^ |  |  |  |
| Cross-Partner Within-Domain |  |  |  |  |  |  |  |  |
| F. Neg. _W-1_ → M. Neg. | ̶ | **.14*^h^** | **.10*^h^** | **.14*^h^** | **.13*^h^** |  |  |  |
| F. Sat. _W-1_ → M. Sat. | ̶ | .01^i^ | .01^i^ | .01^i^ | .01^i^ |  |  |  |
| M. Neg. _W-1_ → F. Neg. | ̶ | **.22*^j^** | **.16*^j^** | **.20*^j^** | **.16*^j^** |  |  |  |
| M. Sat. _W-1_ → F. Sat. | ̶ | -.07^k^ | -.07^k^ | -.07^k^ | -.07^k^ |  |  |  |
| Autoregressive Paths |  |  |  |  |  |  |  |  |
| F. Negative | ̶ | .04^L^ | .03^L^ | .04^L^ | .04^L^ |  |  |  |
| F. Satisfaction | ̶ | .02^m^ | .02^m^ | .02^m^ | .02^m^ |  |  |  |
| M. Negative | ̶ | -.03^n^ | -.02^n^ | -.03^n^ | -.02^n^ |  |  |  |
| M. Satisfaction | ̶ | .01^o^ | .01^o^ | .01^o^ | .02^o^ |  |  |  |
| Within-Time Correlations |  |  |  |  |  |  |  |  |
| F. Neg.↔ F. Sat. | -.27*^p^ | -.27*^p^ | -.23*^p^ | -.25*^p^ | -.24*^p^ |  |  |  |
| F. Neg.↔ M. Neg. | .16*^q^ | .25*^q^ | .18*^q^ | .26*^q^ | .27*^q^ |  |  |  |
| F. Neg.↔ M. Sat. | -.13*^r^ | -.14*^r^ | -.12*^r^ | -.13*^r^ | -.15*^r^ |  |  |  |
| F. Sat. ↔ M. Neg. | -.19*^s^ | -.22*^s^ | -.17*^s^ | -.23*^s^ | -.20*^s^ |  |  |  |
| F. Sat. ↔ M. Sat. | .37*^t^ | .33*^t^ | .28*^t^ | .29*^t^ | .29*^t^ |  |  |  |
| M. Neg.↔ M. Sat. | -.21*^u^ | -.28*^u^ | -.20*^u^ | -.28*^u^ | -.29*^u^ |  |  |  |

*Notes.* Standardized estimates. ^a - u^Corresponding coefficients are constrained to equality. Significant cross-lagged paths are bolded for clarity. _W-1_Preceding Wave. F. = Female partner. M. = Male partner. Neg. = Negative communication. Sat. = Relationship satisfaction. W = Wave. The intercepts and slopes were regressed on relationship duration. Model fit indices: χ^2^(153) = 162.247; RMSEA = .013 (.000, .028); CFI = .998; TLI = .997; SRMR = .044. **p* < .05

Supplementary Figure 3

*Comparison Between the Longitudinal Standardized Regression Coefficients from the Observed PASEZ Positive Communication and Relationship Satisfaction Bivariate (see Table 2) and Dyadic Models (see Supplementary Table 7)*


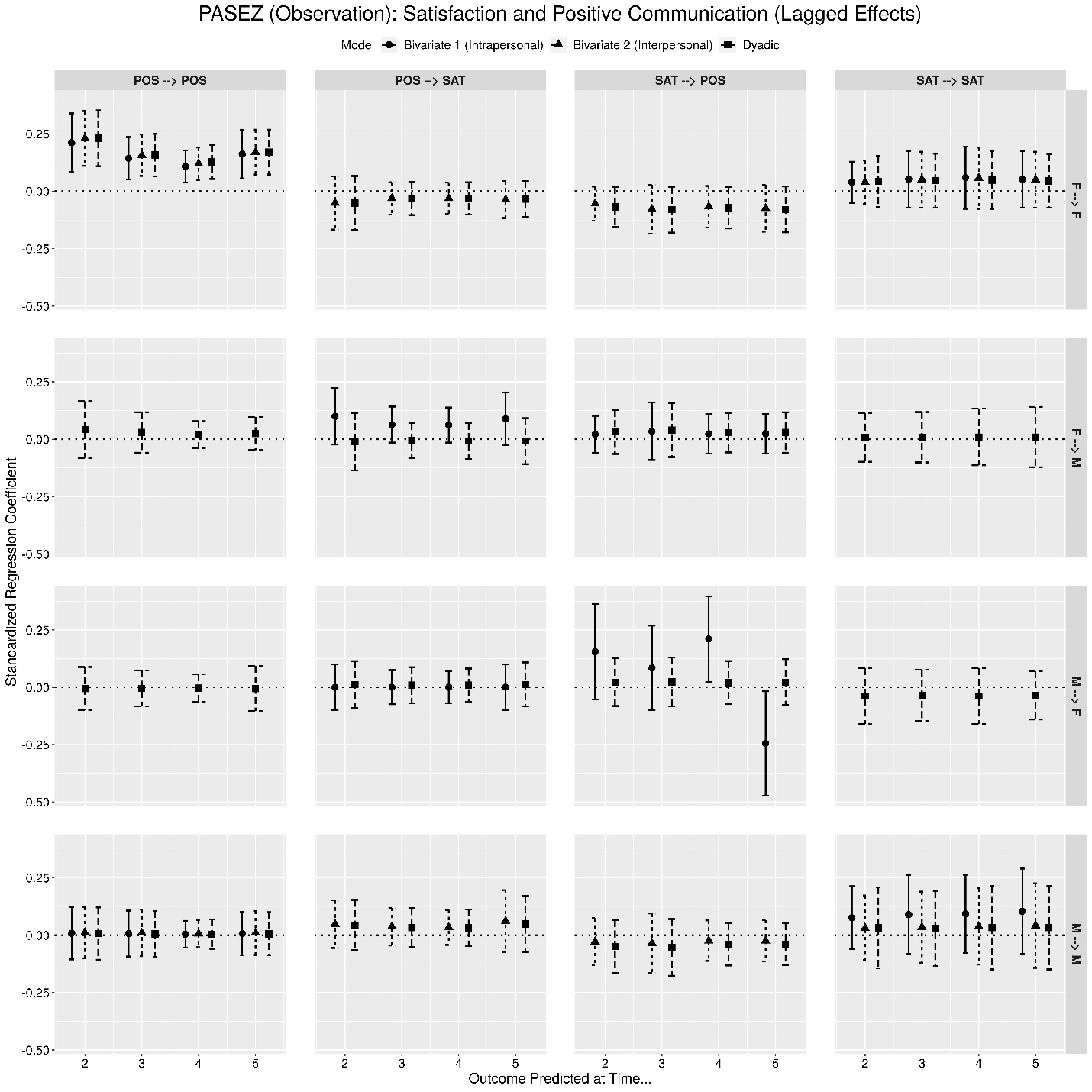


*Notes*. POS = Positive communication. SAT = Relations satisfaction. M = Male partner. F = Female partner. The arrow (-->) indicates the direction of the effect. The bivariate model 1 contains intrapersonal effects (e.g., effects of female partner satisfaction on female partner communication), whereas the bivariate model 2 contains interpersonal effects (e.g., effects of female partner satisfaction on male partner communication).

Supplementary Figure 4

*Comparison Between the Longitudinal Standardized Regression Coefficients from the Observed PASEZ Negative Communication and Relationship Satisfaction Bivariate (see Table 3) and Dyadic Models (see Supplementary Table 8)*


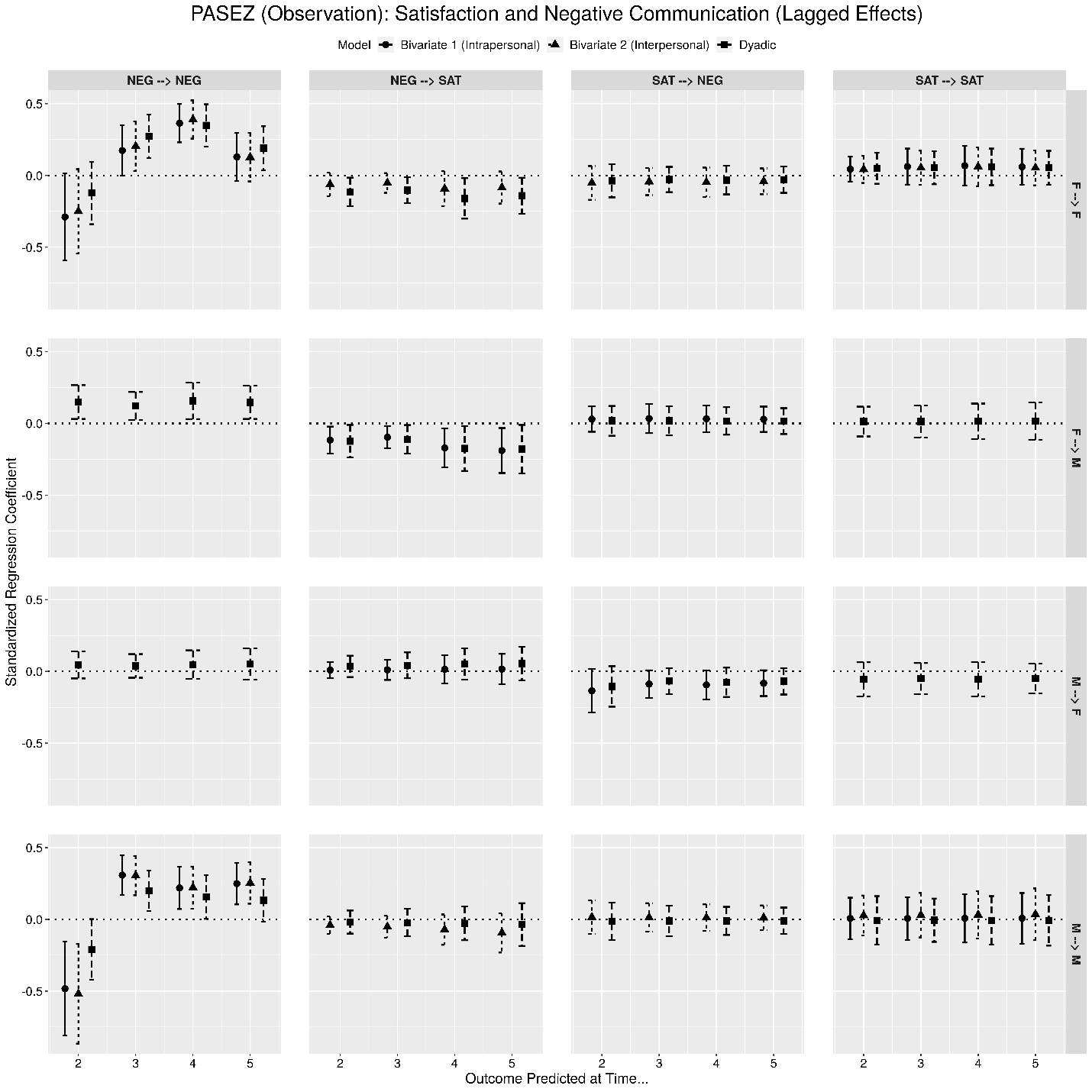


*Notes.* NEG = Negative communication. SAT = Relationship satisfaction. M = Male partner. F = Female partner. The arrow (-->) indicates the direction of the effect. The bivariate model 1 contains intrapersonal effects (e.g., effects of female partner satisfaction on female partner communication), whereas the bivariate model 2 contains interpersonal effects (e.g., effects of female partner satisfaction on male partner communication).

Supplementary Figure 5

*Comparison Between the Within-Time Standardized Regression Coefficients from the Observed PASEZ Positive and Negative Communication and Relationship Satisfaction Bivariate (see Tables 2 and 3) and Dyadic Models (see Supplementary Tables 7 and 8)*


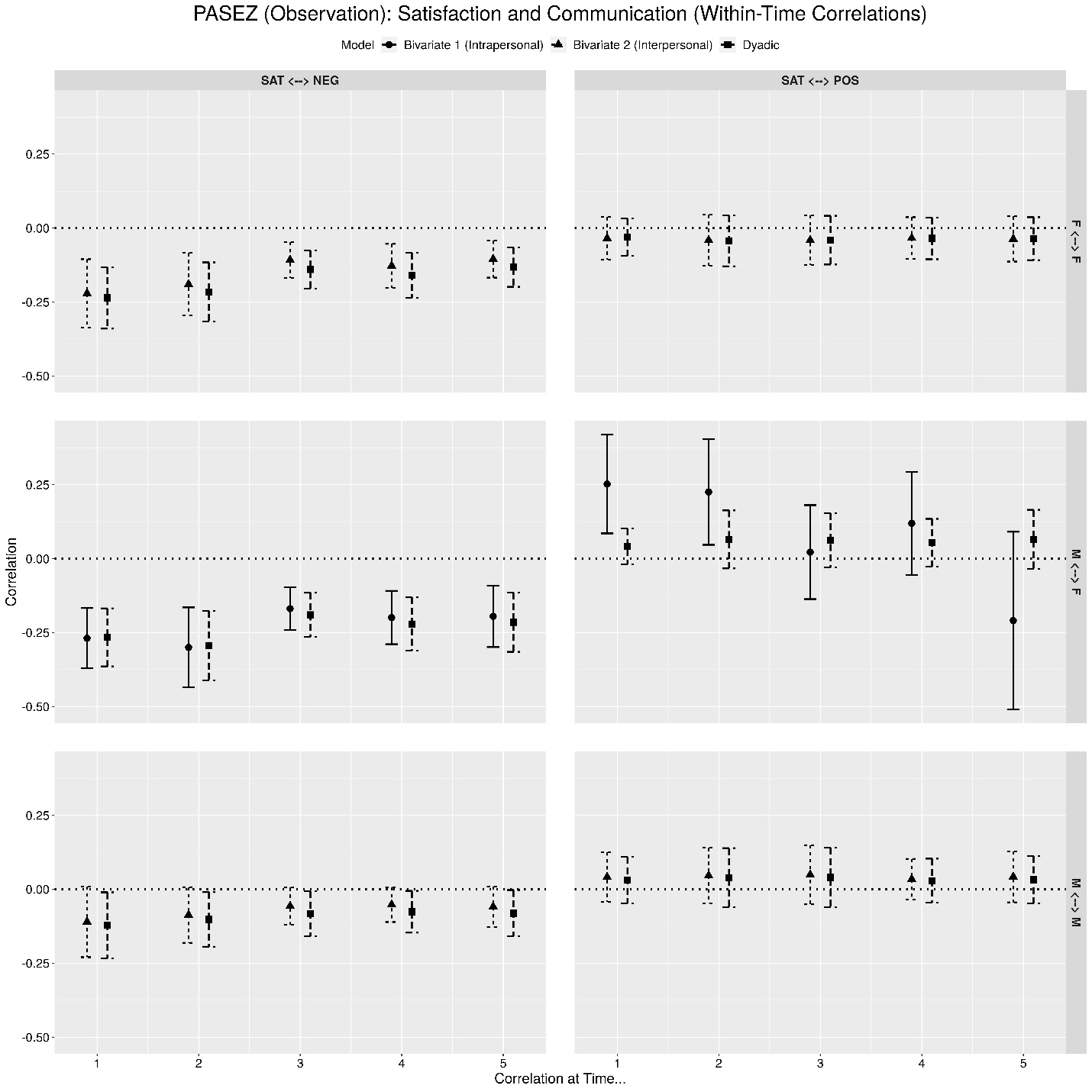


*Notes.* NEG = Negative communication. POS = Positive communication. SAT = Relationship satisfaction. M = Male partner. F = Female partner. The bivariate model 1 contains intrapersonal effects (e.g., correlation between male partner satisfaction and male partner communication), whereas the bivariate model 2 contains interpersonal effects (e.g., correlations between female partner satisfaction and male partner communication).

Supplementary Figure 6

*Comparison Between the Longitudinal Standardized Regression Coefficients from the Self-Report PASEZ Positive Communication and Relationship Satisfaction Bivariate (see Table 4) and Dyadic Models (see Supplementary Table 9)*


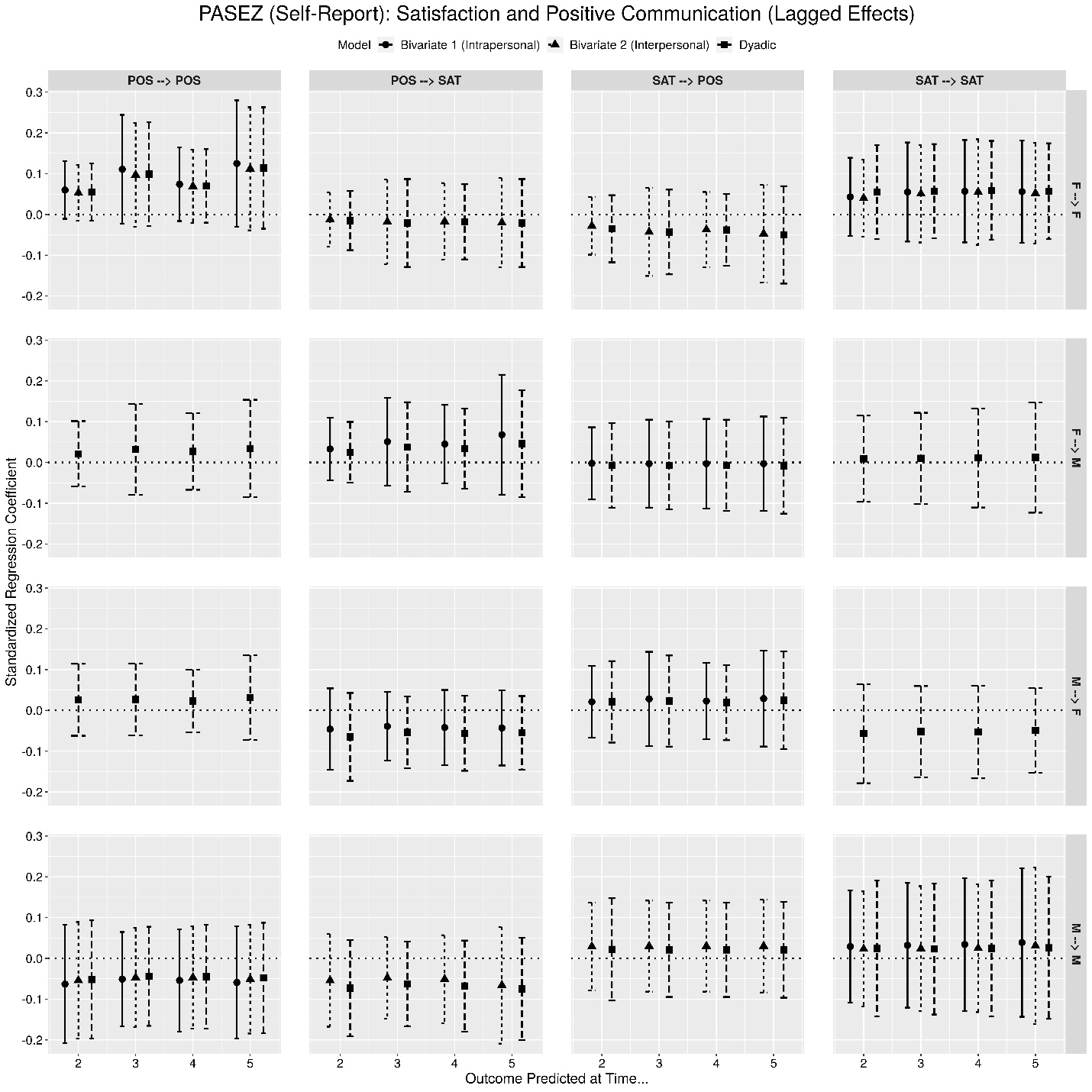


*Notes.* POS = Positive communication. SAT = Relationship satisfaction. M = Male partner. F = Female partner. The arrow (-->) indicates the direction of the effect. The bivariate model 1 contains intrapersonal effects (e.g., effects of female partner satisfaction on female partner communication), whereas the bivariate model 2 contains interpersonal effects (e.g., effects of female partner satisfaction on male partner communication).

Supplementary Figure 7

*Comparison Between the Longitudinal Standardized Regression Coefficients from the Self-Report PASEZ Negative Communication and Relationship Satisfaction Bivariate (see Table 5) and Dyadic Models (see Supplementary Table 10)*


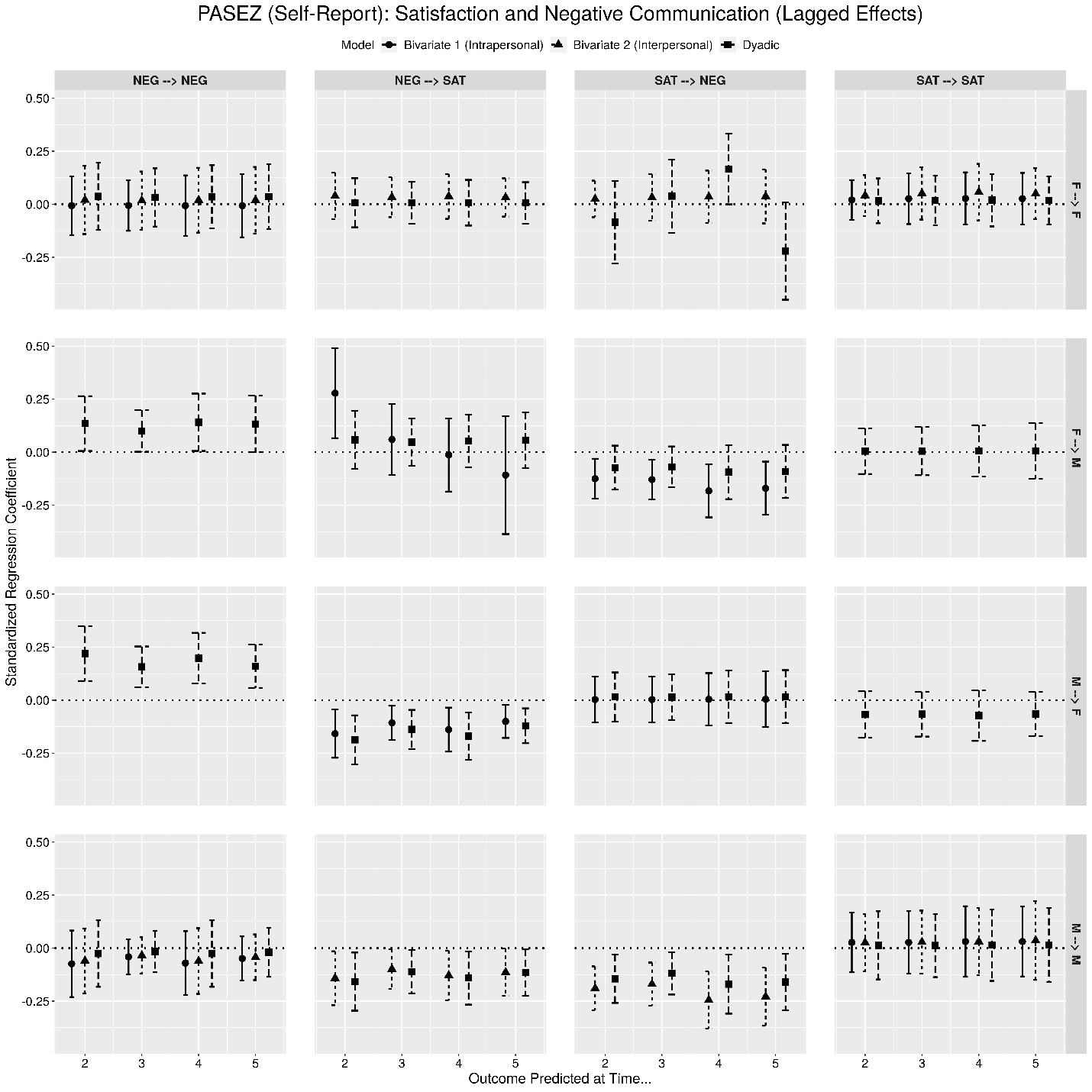


*Notes.* NEG = Negative communication. SAT = Relationship satisfaction. M = Male partner. F = Female partner. The arrow (-->) indicates the direction of the effect. The bivariate model 1 contains intrapersonal effects (e.g., effects of female partner satisfaction on female partner communication), whereas the bivariate model 2 contains interpersonal effects (e.g., effects of female partner satisfaction on male partner communication).

Supplementary Figure 8

*Comparison Between the Within-Time Standardized Regression Coefficients from the Self-Report PASEZ Positive and Negative Communication and Relationship Satisfaction Bivariate (see Tables 4 and 5) and Dyadic Models (see Supplementary Tables 9 and 10)*


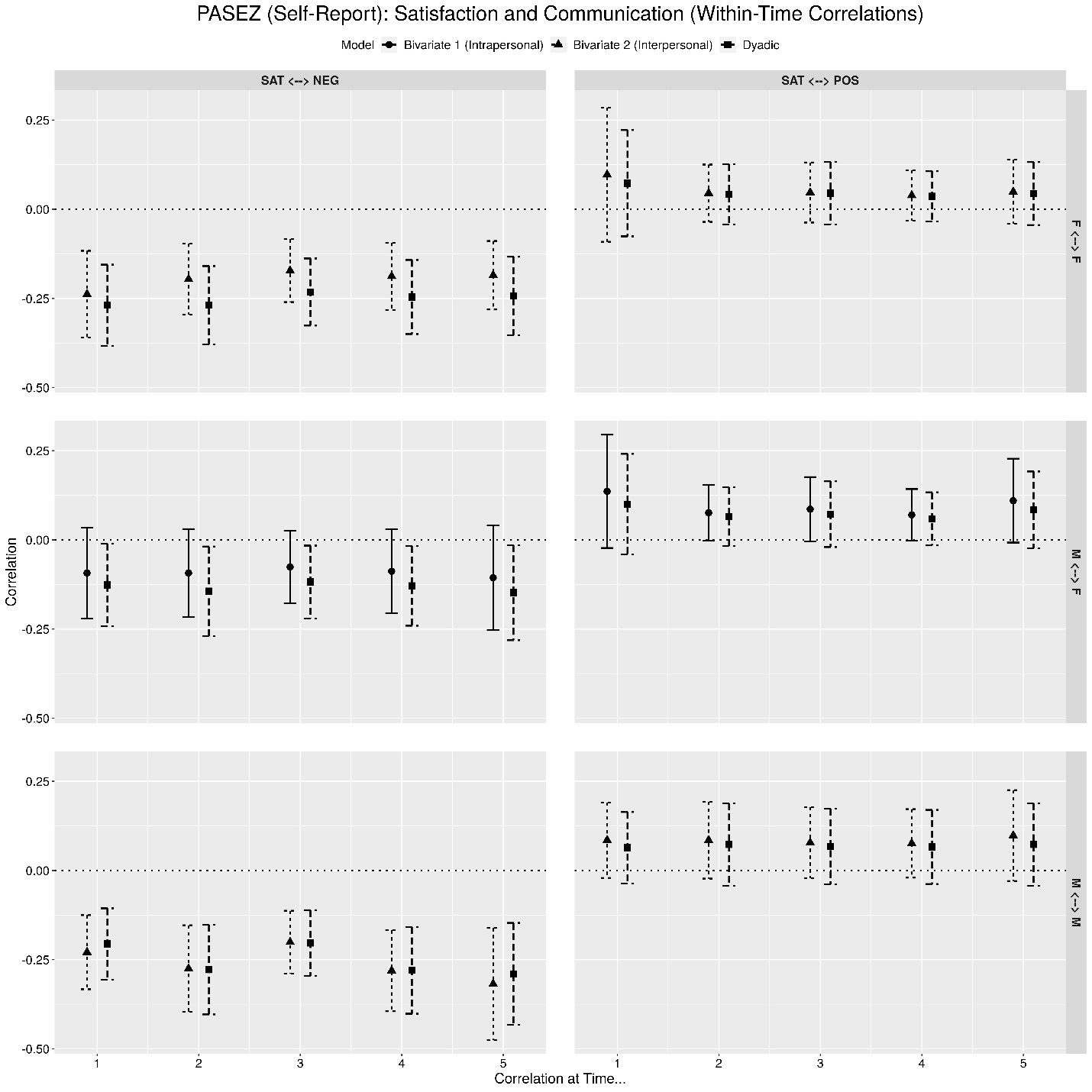


*Notes.* NEG = Negative communication. POS = Positive communication. SAT = Relationship satisfaction. M = Male partner. F = Female partner. The bivariate model 1 contains intrapersonal effects (e.g., correlation between male partner satisfaction and male partner communication), whereas the bivariate model 2 contains interpersonal effects (e.g., correlations between female partner satisfaction and male partner communication).

**Study 3 (Pairfam): Dyadic ALT-SR Modeling Results**

***Positive Communication and Relationship Satisfaction***

The fully dyadic ALT-SR model results that estimate associations between male and female partner positive communication and relationship satisfaction simultaneously in pairfam are shown in Supplementary Table 21. The pattern of results is consistent with those in the bivariate models (see Supplementary Figures 9 and 11 for comparisons of the coefficients).

Supplementary Table 23

*Standardized Dyadic ALT-SR Modeling Results for Male and Female Partner Positive Communication and Relationship Satisfaction in the Pairfam Study (n = 3,405 couples)*

| **Between-Person Results** | 1 | 2 | 3 | 4 | 5 | 6 | 7 | 8 |
| --- | --- | --- | --- | --- | --- | --- | --- | --- |
| 1. F. Pos. Intercept | ̶ |  |  |  |  |  |  |  |
| 2. F. Pos. Slope | -.30* | ̶ |  |  |  |  |  |  |
| 3. F. Sat. Intercept | .23* | .05 | ̶ |  |  |  |  |  |
| 4. F. Sat. Slope | .09 | .09 | -.29* | ̶ |  |  |  |  |
| 5. M. Pos. Intercept | .22* | .06 | .25* | -.01 | ̶ |  |  |  |
| 6. M. Pos. Slope | -.06 | .30 | -.03 | .10 | -.08 | ̶ |  |  |
| 7. M. Sat. Intercept | .23* | .10 | .49* | -.03 | .35* | -.25 | ̶ |  |
| 8. M. Sat. Slope | .04 | -.36 | .21 | .18 | .07 | .46 | -.02 | ̶ |
| **Within-Person Results** | W1 | W2 | W3 | W4 | W5 |  |  |  |
| Cross-Lagged Paths |  |  |  |  |  |  |  |  |
| Within-Partner Cross-Domain |  |  |  |  |  |  |  |  |
| F. Pos._W-1_ → F. Sat. | ̶ | .00^a^ | .00^a^ | .00^a^ | .00^a^ |  |  |  |
| F. Sat. _W-1_ → F. Pos. | ̶ | .00^b^ | .00^b^ | .00^b^ | .00^b^ |  |  |  |
| M. Pos. _W-1_ → M. Sat. | ̶ | -.01^c^ | -.01^c^ | -.01^c^ | -.01^c^ |  |  |  |
| M. Sat. _W-1_ → M. Pos. | ̶ | -.02^d^ | -.02^d^ | -.02^d^ | -.03^d^ |  |  |  |
| Cross-Partner Cross-Domain |  |  |  |  |  |  |  |  |
| F. Pos. _W-1_ → M. Sat. | ̶ | **-.07*** | .00 | .05 | **.12*** |  |  |  |
| F. Sat. _W-1_ → M Pos. | ̶ | .03^e^ | .03^e^ | .03^e^ | .03^e^ |  |  |  |
| M. Pos. _W-1_ → F. Sat. | ̶ | .03^f^ | .02^f^ | .02^f^ | .03^f^ |  |  |  |
| M. Sat. _W-1_ → F Pos. | ̶ | -.01^g^ | -.01^g^ | -.01^g^ | -.02^g^ |  |  |  |
| Cross-Partner Within-Domain |  |  |  |  |  |  |  |  |
| F. Pos. _W-1_ → M. Pos. | ̶ | .01^h^ | .01^h^ | .01^h^ | .01^h^ |  |  |  |
| F. Sat. _W-1_ → M. Sat. | ̶ | .04^i^ | .05^i^ | .04^i^ | .04^i^ |  |  |  |
| M. Pos. _W-1_ → F. Pos. | ̶ | -.01^j^ | -.01^j^ | -.01^j^ | -.01^j^ |  |  |  |
| M. Sat. _W-1_ → F. Sat. | ̶ | .04^k^ | .04^k^ | .04^k^ | .05^k^ |  |  |  |
| Autoregressive Paths |  |  |  |  |  |  |  |  |
| F. Positive | ̶ | -.16* | .02 | .07* | .09* |  |  |  |
| F. Satisfaction | ̶ | .05*^L^ | .05*^L^ | .05*^L^ | .05*^L^ |  |  |  |
| M. Positive | ̶ | .03^m^ | .03^m^ | .03^m^ | .03^m^ |  |  |  |
| M. Satisfaction | ̶ | .11*^n^ | .10*^n^ | .09*^n^ | .12*^n^ |  |  |  |
| Within-Time Correlations |  |  |  |  |  |  |  |  |
| F. Pos. ↔ F. Sat. | .06*^o^ | .06*^o^ | .06*^o^ | .06*^o^ | .07*^o^ |  |  |  |
| F. Pos. ↔ M. Pos. | .00^p^ | .00^p^ | .00^p^ | .00^p^ | .00^p^ |  |  |  |
| F. Pos. ↔ M. Sat. | -.01 | .00 | .05 | -.01 | .18* |  |  |  |
| F. Sat. ↔ M. Pos. | .05*^q^ | .05*^q^ | .06*^q^ | .06*^q^ | .07*^q^ |  |  |  |
| F. Sat. ↔ M. Sat. | .04 | .19* | .11* | .04 | .27* |  |  |  |
| M. Pos. ↔ M. Sat. | .05*^r^ | .06*^r^ | .06*^r^ | .05*^r^ | .07*^r^ |  |  |  |

*Notes.* Standardized estimates. ^a - r^Corresponding coefficients are constrained to equality. Significant cross-lagged paths are bolded for clarity. _W-1_Preceding Wave. F. = Female partner. M. = Male partner. Pos. = Positive communication. Sat. = Relationship satisfaction. W = Wave. The intercepts and slopes were regressed on relationship duration. Model fit indices: χ^2^(142) = 163.715; RMSEA = .007 (.000, .011); CFI = .997; TLI = .996; SRMR = .020. **p* < .05.

**Study 3 (Pairfam): Dyadic ALT-SR Modeling Results**

***Negative Communication and Relationship Satisfaction***

The fully dyadic ALT-SR model results for negative communication and relationship satisfaction in pairfam are shown in Supplementary Table 22. The pattern of results is, again, generally consistent with those in the bivariate models (see Supplementary Figures 10 and 11 for comparisons of the coefficients), with two exceptions. The cross-lagged association between deviations in female partners’ negative communication and their male partner’s future relationship satisfaction were not significant in the fully dyadic model, although the coefficients are nearly identical to those in the bivariate model. In the dyadic model only, more frequent than typical negative communication in the male partner predicted a future intraindividual decrease in his own relationship satisfaction at each wave. Once again, however, the coefficients were nearly identical to those in the bivariate model.

Supplementary Table 24

*Standardized Dyadic ALT-SR Modeling Results for Male and Female Partner Negative Communication and Relationship Satisfaction in the Pairfam Study (n = 3,405 couples)*

| **Between-Person Results** | 1. | 2. | 3. | 4. | 5. | 6. | 7. | 8. |
| --- | --- | --- | --- | --- | --- | --- | --- | --- |
| 1. F. Neg. Intercept | ̶ |  |  |  |  |  |  |  |
| 2. F. Neg. Slope | -.27* | ̶ |  |  |  |  |  |  |
| 3. F. Sat. Intercept | -.44* | .02 | ̶ |  |  |  |  |  |
| 4. F. Sat. Slope | -.02 | -.37* | -.22 | ̶ |  |  |  |  |
| 5. M. Neg. Intercept | .36* | .09 | -.35* | -.06 | ̶ |  |  |  |
| 6. M. Neg. Slope | .17 | .03 | -.21 | -.08 | .03 | ̶ |  |  |
| 7. M. Sat. Intercept | -.34* | .03 | .47* | .06 | -.45* | -.01 | ̶ |  |
| 8. M. Sat. Slope | -.09 | -.23 | .40 | .06 | -.20 | -.57 | .22 | ̶ |
| **Within-Person Results** | W1 | W2 | W3 | W4 | W5 |  |  |  |
| Cross-Lagged Paths |  |  |  |  |  |  |  |  |
| Within-Partner Cross-Domain |  |  |  |  |  |  |  |  |
| F. Neg. _W-1_ → F. Sat. | ̶ | -.03^a^ | -.03^a^ | -.03^a^ | -.03^a^ |  |  |  |
| F. Sat. _W-1_ → F. Neg. | ̶ | **-.08*** | .03 | -.03 | .07 |  |  |  |
| M. Neg. _W-1_ → M. Sat. | ̶ | **-.05*^b^** | **-.04*^b^** | **-.03*^b^** | **-.04*^b^** |  |  |  |
| M. Sat. _W-1_ → M. Neg. | ̶ | -.02^c^ | -.02^c^ | -.02^c^ | -.02^c^ |  |  |  |
| Cross-Partner Cross-Domain |  |  |  |  |  |  |  |  |
| F. Neg. _W-1_ → M. Sat. | ̶ | -.04^d^ | -.04^d^ | -.03^d^ | -.04^d^ |  |  |  |
| F. Sat. _W-1_ → M Neg. | ̶ | **-.11*** | .01 | .00 | -.04 |  |  |  |
| M. Neg. _W-1_ → F. Sat. | ̶ | -.04^e^ | -.03^e^ | -.03^e^ | -.03^e^ |  |  |  |
| M. Sat. _W-1_ → F Neg. | ̶ | -.01^f^ | -.01^f^ | -.01^f^ | -.01^f^ |  |  |  |
| Cross-Partner Within-Domain |  |  |  |  |  |  |  |  |
| F. Neg. _W-1_ → M. Neg. | ̶ | .03^g^ | .03^g^ | .03^g^ | .02^g^ |  |  |  |
| F. Sat. _W-1_ → M. Sat. | ̶ | .04^h^ | .04^h^ | .03^h^ | .04^h^ |  |  |  |
| M. Neg. _W-1_ → F. Neg. | ̶ | .05^i^ | .04^i^ | .04^i^ | .04^i^ |  |  |  |
| M. Sat. _W-1_ → F. Sat. | ̶ | .04^j^ | .04^j^ | .04^j^ | .04^j^ |  |  |  |
| Autoregressive Paths |  |  |  |  |  |  |  |  |
| F. Negative | ̶ | .06^k^ | .05^k^ | .06^k^ | .05^k^ |  |  |  |
| F. Satisfaction | ̶ | .05*^L^ | .05*^L^ | .05*^L^ | .06*^L^ |  |  |  |
| M. Negative | ̶ | .03^m^ | .03^m^ | .03^m^ | .03^m^ |  |  |  |
| M. Satisfaction | ̶ | .12*^n^ | .10*^n^ | .09*^n^ | .12*^n^ |  |  |  |
| Within-Time Correlations |  |  |  |  |  |  |  |  |
| F. Neg.↔ F. Sat. | -.17*^o^ | -.18*^o^ | -.18*^o^ | -.19*^o^ | -.20*^o^ |  |  |  |
| F. Neg.↔ M. Neg. | .14*^p^ | .16*^p^ | .17*^p^ | .19*^p^ | .16*^p^ |  |  |  |
| F. Neg.↔ M. Sat. | -.10*^q^ | -.12*^q^ | -.12*^q^ | -.11*^q^ | -.12*^q^ |  |  |  |
| F. Sat. ↔ M. Neg. | -.12*^r^ | -.13*^r^ | -.14*^r^ | -.15*^r^ | -.14*^r^ |  |  |  |
| F. Sat. ↔ M. Sat. | .07 | .18* | .12* | .05 | .24* |  |  |  |
| M. Neg.↔ M. Sat. | -.15*^s^ | -.18*^s^ | -.19*^s^ | -.17*^s^ | -.17*^s^ |  |  |  |

*Notes.* Standardized estimates. ^a - s^Corresponding coefficients are constrained to equality. Significant cross-lagged paths are bolded for clarity. _W-1_Preceding Wave. F. = Female partner. M. = Male partner. Neg. = Negative communication. Sat. = Relationship satisfaction. W = Wave. The intercepts and slopes were regressed on relationship duration. Model fit indices: χ^2^(146) = 204.592; RMSEA = .011 (.007, .014); CFI = .996; TLI = .994; SRMR = .020. **p* < .05.

Supplementary Figure 9

*Comparison Between the Longitudinal Standardized Regression Coefficients from the Pairfam Positive Communication and Relationship Satisfaction Bivariate (see Table 6) and Dyadic Models (see Supplementary Table 13)*


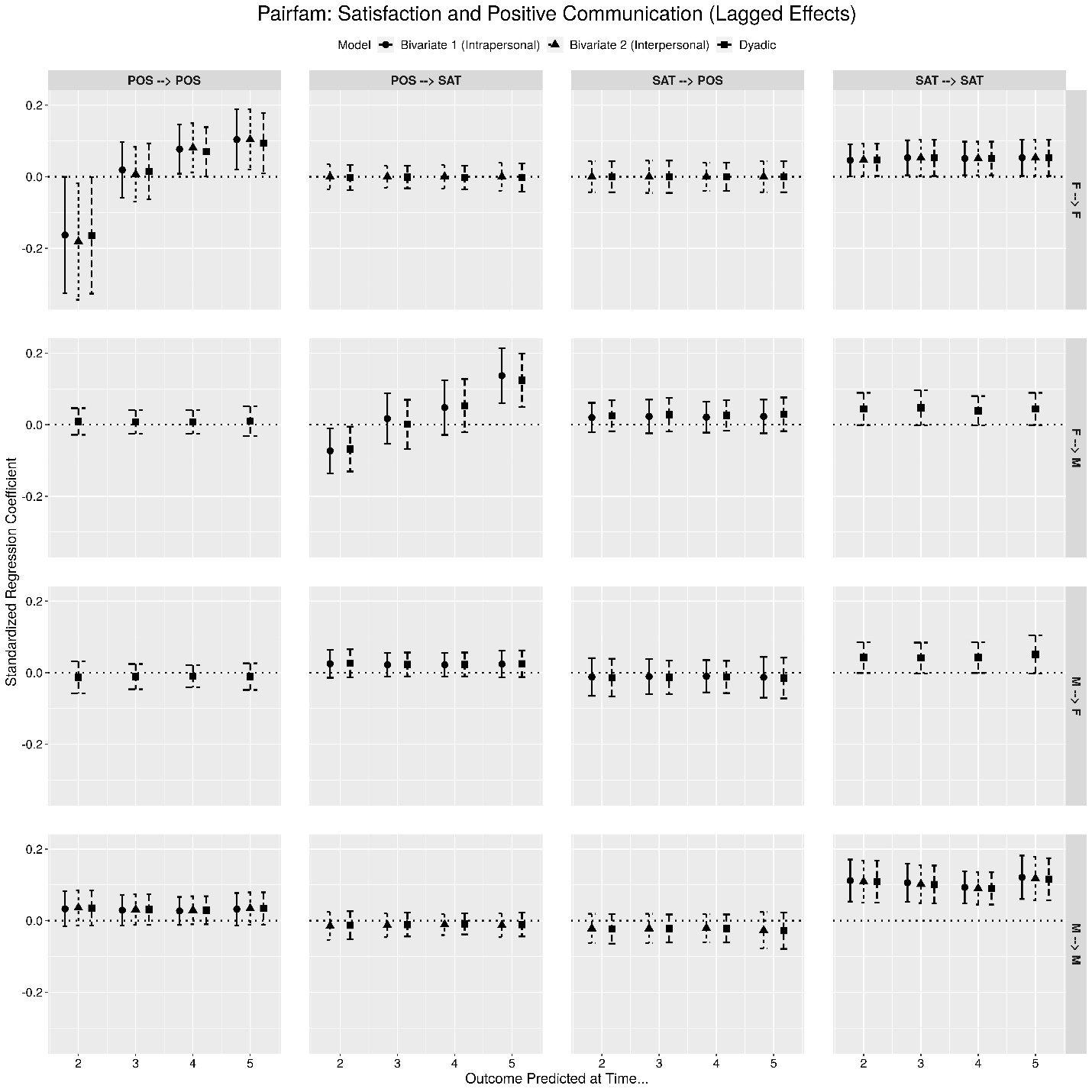


*Notes* POS = Positive communication. SAT = Relationship satisfaction. M = Male partner. F = Female partner. The arrow (-->) indicates the direction of the effect. The bivariate model 1 contains intrapersonal effects (e.g., effects of female partner satisfaction on female partner communication), whereas the bivariate model 2 contains interpersonal effects (e.g., effects of female partner satisfaction on male partner communication).

Supplementary Figure 10

*Comparison Between the Longitudinal Standardized Regression Coefficients from the PASEZ Negative Communication and Relationship Satisfaction Bivariate (see Table 7) and Dyadic Models (see Supplementary Table 14)*


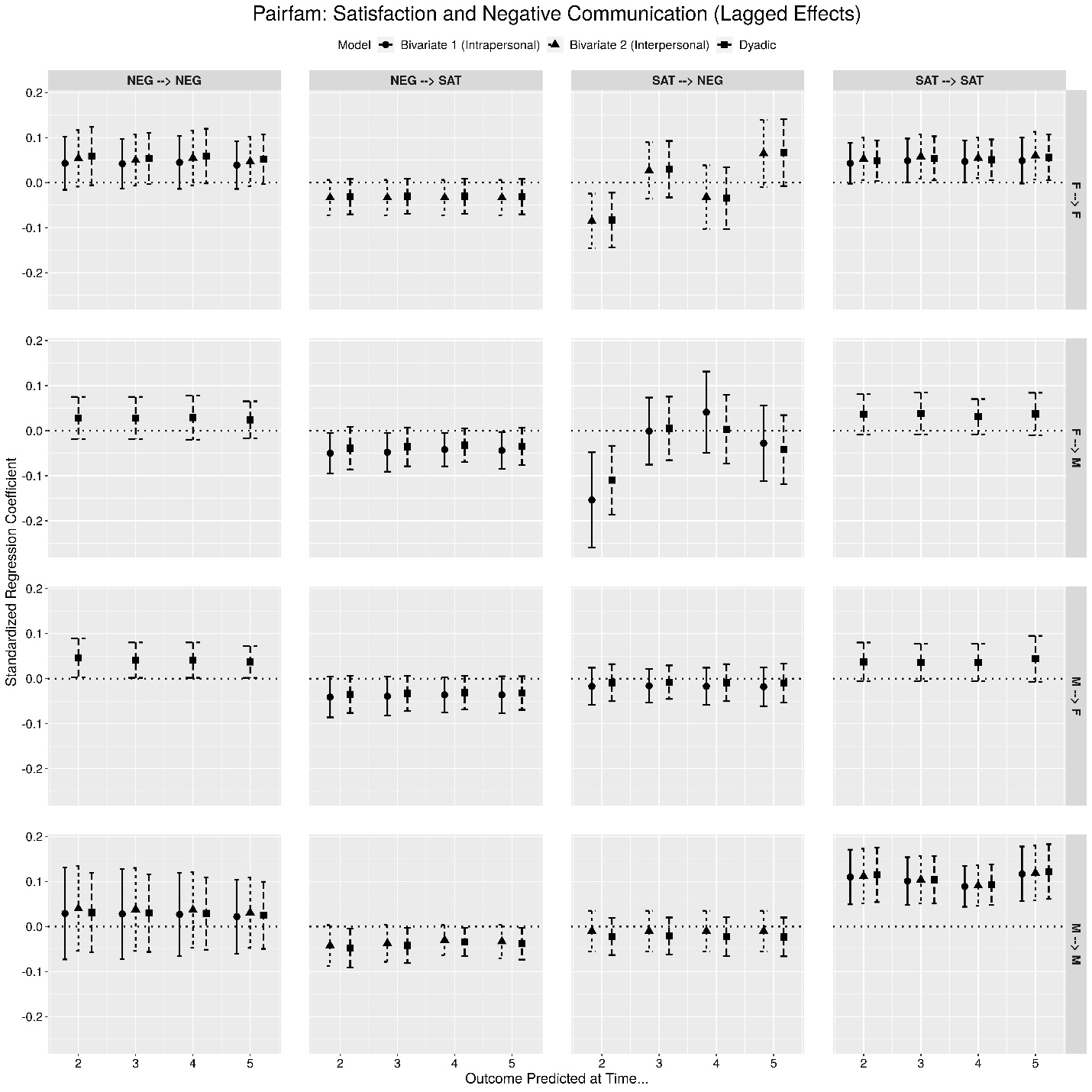


*Notes.* NEG = Negative communication. SAT = Relationship satisfaction. M = Male partner. F = Female partner. The arrow (-->) indicates the direction of the effect. The bivariate model 1 contains intrapersonal effects (e.g., effects of female partner satisfaction on female partner communication), whereas the bivariate model 2 contains interpersonal effects (e.g., effects of female partner satisfaction on male partner communication).

Supplementary Figure 11

*Comparison Between the Within-Time Standardized Regression Coefficients from the PASEZ Positive and Negative Communication and Relationship Satisfaction Bivariate (see Tables 6 and 7) and Dyadic Models (see Supplementary Tables 13 and 14)*


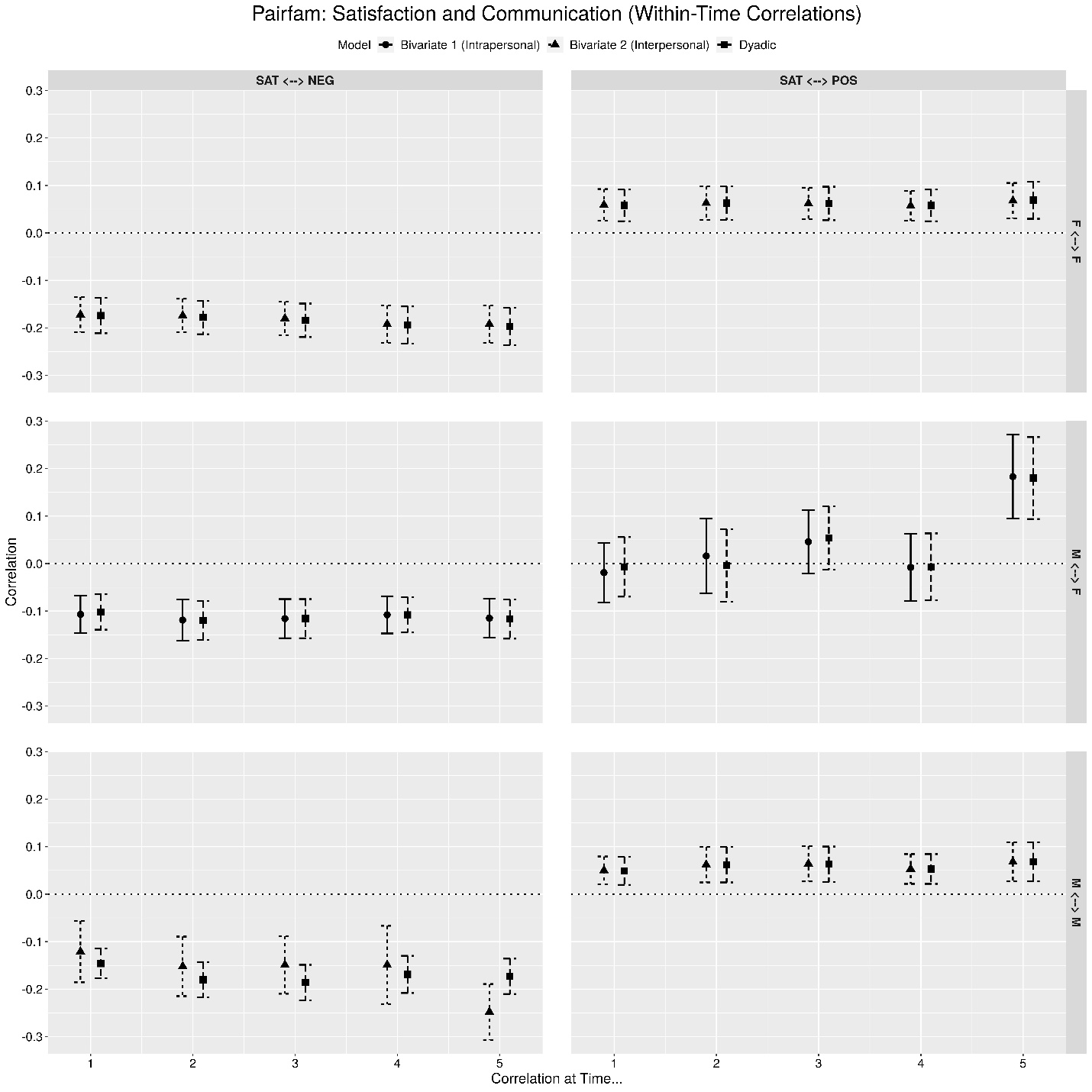


*Notes.* NEG = Negative communication. POS = Positive communication. SAT = Relationship satisfaction. M = Male partner. F = Female partner. The bivariate model 1 contains intrapersonal effects (e.g., correlation between male partner satisfaction and male partner communication), whereas the bivariate model 2 contains interpersonal effects (e.g., correlations between female partner satisfation and male partner communication).

**7. Overview of Study 4: The CouPers Study**

**Method**

**Procedure.** The data analyzed in Study 4 are taken from the *Processes in Romantic Relationships and Their Impact on Relationship and Personal Outcomes (CouPers)* study. The CouPers study is a multi-wave longitudinal online study of couples conducted between 2016 and 2018 at the University of Basel, Switzerland.^[[1]](#footnote-1)^ The primary purpose of the study was to investigate the associations between personality and romantic relationship outcomes. Participants were recruited from the student population, the local community, and via Facebook advertisements targeted at residents of Switzerland, Germany, and Austria who reported being in a relationship. Eligible participants were over 18 years old, had a partner who was older than 18 years and also willing to participate, had been in their relationship for at least one month, and were able to respond to surveys written in the German language.

The study consisted of four waves. Each wave included 14 days of online daily diary surveys with an additional battery of surveys on day 1 and day 14. Waves 1 through 3 were separated by a roughly six-month interval; Waves 3 and 4 were separated by an interval of about 12 months. Participants entered Wave 1 in one of 12 monthly cohorts. In subsequent waves, couples were permitted to move to a different cohort or to skip a wave if they were unable to participate at the scheduled time. Participants were allowed to participate as singletons if their initial relationship ended. New partners were invited to join the study. If participants completed the long surveys on days 1 and 14 and at least seven of the 14 daily surveys they received personalized feedback on a selected measure in the study (if desired) and were compensated with a shopping or cinema voucher worth 20 CHF or €20 per wave.

At the start of the study, 1,313 couples consented for surveys to be emailed to both partners. During the study, 10 new partners were recruited. Three participants asked that their data be deleted, and a further 437 participants (belonging to 272 couples) withdrew (explicitly requested to discontinue participation). During Wave 1, 2,317 participants at least partially responded to surveys (including 1,111 couples in which both partners responded); during Wave 2, 1,760 (819 couples); during Wave 3, 1,481 (672 couples); and during Wave 4, 1,265 participants (549 couples). Ethics approval for the study was granted by the ethics committee of the Department of Psychology at the University of Basel, Switzerland (proposal title: Processes in romantic relationships and their impact on relationship and personal outcomes; protocol number: 003-16-1).

**Participants.** Positive and negative communication were only assessed in the daily diary portion of Wave 4, so we used data only from that wave for the present analyses. Of the 503 eligible couples, 483 couples provided data on the relevant variables in the daily surveys.

At Wave 1, the selected couples for the present analysis had been in their relationships for 8.07 years, on average (*SD* = 10.52 years); 33.8% of the participants were married. Female partners had an average age of 37.39 (*SD* = 17.29) years, male partners had an average age of 39.59 (*SD* = 18.14) years. Regarding parental status, 30.9% of the participants had children. Of those, 38.7% had one child, 40.0% had two children, and 21.3% had three or more children. In terms of education, 55% of the women and 51.8% of the men earned a University degree (or equivalent). Most of the women (62%) had a yearly income up to 40,000 Euros or CHF; only 1% had an income higher than 120,000 Euros or CHF. Similarly, most of the men (48%) had a yearly income of up to 40,000 Euros or CHF; 2.8% had an income higher than 120,000 Euros or CHF.

**Measures.** Descriptive statistics and correlations among all study variables are available in Supplementary Tables 23 and 24.

***Positive Communication.*** Positive communication during conflicts was assessed using the German version of the Conflict Resolution Styles Inventory (CRSI; Herzberg & Sierau, 2010). The CRSI was only presented when the respondents indicated they had a conflict that day. Positive communication was assessed with two items: “We sat together and calmly discussed our conflict,” and “I found solutions that were acceptable for both of us.” Responses ranged from 1 = *does not apply at all* to 5 = *fully applies* and mean scores were computed. Inter-item correlations across the 14 days ranged from *r* = .13 to *r* = .87 (average *r* = .42) for men and from *r* = .26 to *r* = .72 (average *r* = .46) for women.

***Negative Communication.*** As with positive communication, the negative communication items were only presented when the participants reported a conflict that day. Items were “I deliberately insulted my partner,” and “I exploded and lost control over myself.” Responses ranged from 1 = *does not apply at all* to 5 = *fully applies* and mean scores were computed. Inter-item correlations across the 14 days ranged from *r* = .07 to *r* = .81 (average *r* = .58) for men and from *r* = .16 to *r* = .70 (average *r* = .50) for women.

***Relationship satisfaction.*** Each day, the participants were asked to evaluate their current level of relationship satisfaction using a single item: “All in all, how satisfied were you with your relationship today?” Responses ranged from 1 = *very dissatisfied* to 5 = *very satisfied*.

**Results**

As a first step in the data analysis, we computed a series of growth curve models for both partners’ relationship satisfaction, positive communication, and negative communication. With regard to women’s relationship satisfaction, a latent basis growth curve model described the data best, although model fit was borderline according to standard benchmarks (χ^2^(90) = 278.816; RMSEA = 0.066; CFI = 0.901; TLI = 0.900; SRMR = 0.094). Model fit was excellent, however, after we added time-invariant structured residuals (χ^2^(87) = 129.680; RMSEA = 0.032; CFI = 0.978; TLI = 0.977; SRMR = 0.05). According to this model, women’s relationship satisfaction remained stable, on average, across the 14 days of the daily surveys (Cohen’s *d* = 0.09, *p* = .318). For the male partners, the best-fitting model was a model with a random linear slope and unconstrained structured residuals (χ^2^(87) = 196.738; RMSEA = 0.052; CFI = 0.942; TLI = 0.940; SRMR = 0.083). For the female partners, there was no change, on average, in relationship satisfaction across the 14 days (Cohen’s *d* = 0.03, *p* = .655).

When repeating the same procedure for positive and negative communication, we encountered severe convergence issues. Even the models that converged had a poor fit to the data. These issues were most likely caused by the low base rate of conflicts in the sample. On average, only 9.33% of the female partners and 6.42% of the male partners reported a conflict on any given day. Across the 14 days, the average number of reported conflicts totaled 0.97 (*SD* = 1.21) for women and 0.64 (*SD* = 0.97) for men, respectively; 45.92% of the women and 59.24% of the men reported no conflict at all across the 14 days. Furthermore, partners did not agree on whether they had a conflict in a given day or not, as indicated by a correlation of *r* = .41. Finally, there were no prolonged episodes of conflict. The longest sequence of days with conflicts averaged 1.2 (*SD* = 0.47) days for women and 1.13 (*SD* = 0.36) days for men (see Supplementary Figure 12A through D).

Despite the modeling issues with the communication variables, we tried to build an ALT-SR as planned. Only three of the eight bivariate models converged—the model linking (a) female satisfaction and female negative communication, (b) male satisfaction and female negative communication, and (c) male satisfaction and female negative communication. To achieve convergence at least for some models, however, all lagged within-person associations had to be removed; the converging models only included between-person correlations, within-time correlations between satisfaction and communication, and autoregressive paths only for relationship satisfaction. The converging models still had a poor fit to the data (range of CFI = 0.798-0.821; range of TLI = 0.792-0.814; range of RMSEA = 0.046-0.050; range of SRMR = 0.194-0.225). Thus, we refrain from interpreting the obtained parameter estimates. The dyadic models did not converge.

**Discussion**

The analysis of the diary data from the CouPers Study was intended to provide insight regarding the association between satisfaction and communication on a daily level. On this very small time scale, however, there seems to be no pattern of association that could be analyzed. Technically, we encountered non-convergence and poor model fit; nevertheless, we believe that this study is still informative for the present research question. Specifically, we found that on a daily level, conflicts appear to be rather rare and circumscribed events. Even if participants experienced a conflict on one day, they were unlikely to experience another conflict the day after (i.e., very short sequences of days with conflicts). The relatively low correspondence between the partners further suggests that the reported conflicts were minor disagreements; for more serious conflicts, we would expect a substantially higher correspondence between couple members. Although it is possible that there is a significant association between positive and negative communication and relationship satisfaction from one day to the next, the rarity of conflict in this sample precluded us from empirically examining this possibility.

Supplementary Table 23

*Average Correlations Across the 14-Day Diary Phase in the CouPers Study Among Communication and Relationship Satisfaction for Male and Female Partners (n = 483 couples)*

| Variable | 1 | 2 | 3 | 4 | 5 | 6 | 7 |
| --- | --- | --- | --- | --- | --- | --- | --- |
| 1. Male Positive | — |  |  |  |  |  |  |
| 2. Male Negative | -.10 | — |  |  |  |  |  |
| 3. Female Positive | .41* | -.20* | — |  |  |  |  |
| 4. Female Negative | -.11 | .19* | -.23* | — |  |  |  |
| 5. Male Satisfaction | .35* | -.15* | .13* | -.18* | — |  |  |
| 6. Female Satisfaction | .30* | -.09 | .31* | -.17* | .55* | — |  |
| 7. Rel. Length | .07 | .00 | -.01 | -.02 | .01 | -.02 | — |
| *Mean* | 2.78 | 1.58 | 2.81 | 1.97 | 4.16 | 4.20 | 8.07 |
| *SD* | 1.05 | 0.78 | 1.01 | 0.92 | 0.63 | 0.60 | 10.52 |

*Notes*. Male = Male partner. Female = Female partner. Positive = Positive communication. Negative = Negative communication. Rel. = Relationship. Range is 1 – 5 for positive/negative communication and 1 – 5 for relationship satisfaction.

^*^ *p* < .05.

Supplementary Table 24

*Means and Standard Deviations of Communication and Satisfaction for Each Day of the Wave 4 Diary Phase in the CouPers Study (n = 483 couples)*

|  | Survey Day | | | | | | | | | | | | | |
| --- | --- | --- | --- | --- | --- | --- | --- | --- | --- | --- | --- | --- | --- | --- |
|  | 1 | 2 | 3 | 4 | 5 | 6 | 7 | 8 | 9 | 10 | 11 | 12 | 13 | 14 |
| Male Negative |  |  |  |  |  |  |  |  |  |  |  |  |  |  |
| Mean | 1.60 | 1.40 | 1.74 | 1.58 | 1.60 | 1.50 | 1.84 | 1.71 | 1.54 | 1.81 | 1.57 | 1.95 | 1.69 | 1.52 |
| SD | 0.82 | 0.76 | 0.94 | 1.05 | 0.79 | 0.77 | 1.01 | 0.92 | 0.72 | 0.79 | 0.76 | 1.25 | 1.03 | 0.62 |
| Female Negative |  |  |  |  |  |  |  |  |  |  |  |  |  |  |
| Mean | 2.12 | 1.91 | 1.84 | 2.04 | 1.95 | 2.11 | 1.92 | 2.11 | 1.68 | 1.96 | 1.81 | 2.39 | 1.87 | 2.00 |
| SD | 1.39 | 1.00 | 0.84 | 1.08 | 1.04 | 1.13 | 1.03 | 1.13 | 0.69 | 0.94 | 0.92 | 0.98 | 0.82 | 0.95 |
| Male Positive |  |  |  |  |  |  |  |  |  |  |  |  |  |  |
| Mean | 2.73 | 2.88 | 2.86 | 2.77 | 2.83 | 2.61 | 2.84 | 2.48 | 2.58 | 2.14 | 2.43 | 2.64 | 2.69 | 2.77 |
| SD | 1.14 | 1.13 | 0.95 | 1.15 | 1.13 | 1.28 | 1.32 | 1.09 | 1.14 | 0.90 | 1.05 | 1.06 | 1.23 | 1.26 |
| Female Positive |  |  |  |  |  |  |  |  |  |  |  |  |  |  |
| Mean | 2.46 | 2..78 | 2.80 | 2.80 | 2.82 | 2.81 | 2.56 | 2.50 | 3.42 | 2.62 | 3.08 | 2.65 | 2.82 | 3.04 |
| SD | 1.03 | 1.16 | 1.15 | 1.32 | 1.09 | 0.88 | 1.11 | 0.98 | 1.08 | 1.09 | 1.14 | 1.06 | 1.09 | 1.14 |
| Male Satisfaction |  |  |  |  |  |  |  |  |  |  |  |  |  |  |
| Mean | 4.17 | 4.14 | 4.23 | 4.20 | 4.17 | 4.21 | 4.21 | 4.14 | 4.08 | 4.09 | 4.07 | 4.07 | 4.26 | 4.29 |
| SD | 0.94 | 0.89 | 0.86 | 0.82 | 0.93 | 0.90 | 0.92 | 0.87 | 0.88 | 0.90 | 0.94 | 0.92 | 0.91 | 0.90 |
| Female Satisfaction |  |  |  |  |  |  |  |  |  |  |  |  |  |  |
| Mean | 4.26 | 4.21 | 4.21 | 4.17 | 4.11 | 4.19 | 4.26 | 4.17 | 4.11 | 4.19 | 4.11 | 4.20 | 4.27 | 4.40 |
| SD | 0.85 | 0.85 | 0.89 | 0.90 | 0.94 | 0.91 | 0.87 | 0.89 | 0.90 | 0.82 | 0.94 | 0.88 | 0.88 | 0.78 |

*Note.* Male = Male partner. Female = Female partner. Positive = Positive communication. Negative = Negative communication. The sample sizes for each day range from 12 to 47 for positive/negative communication. Furthermore, the participants providing data on communication each day change from one day to the next; the table, thus, presents results of 14 different samples within the CouPers Study. Sample size for relationship satisfaction ranges from 315 to 403.

Supplementary Figure 12

*Figures Depicting Information about Couple Conflicts in the CouPers Study*


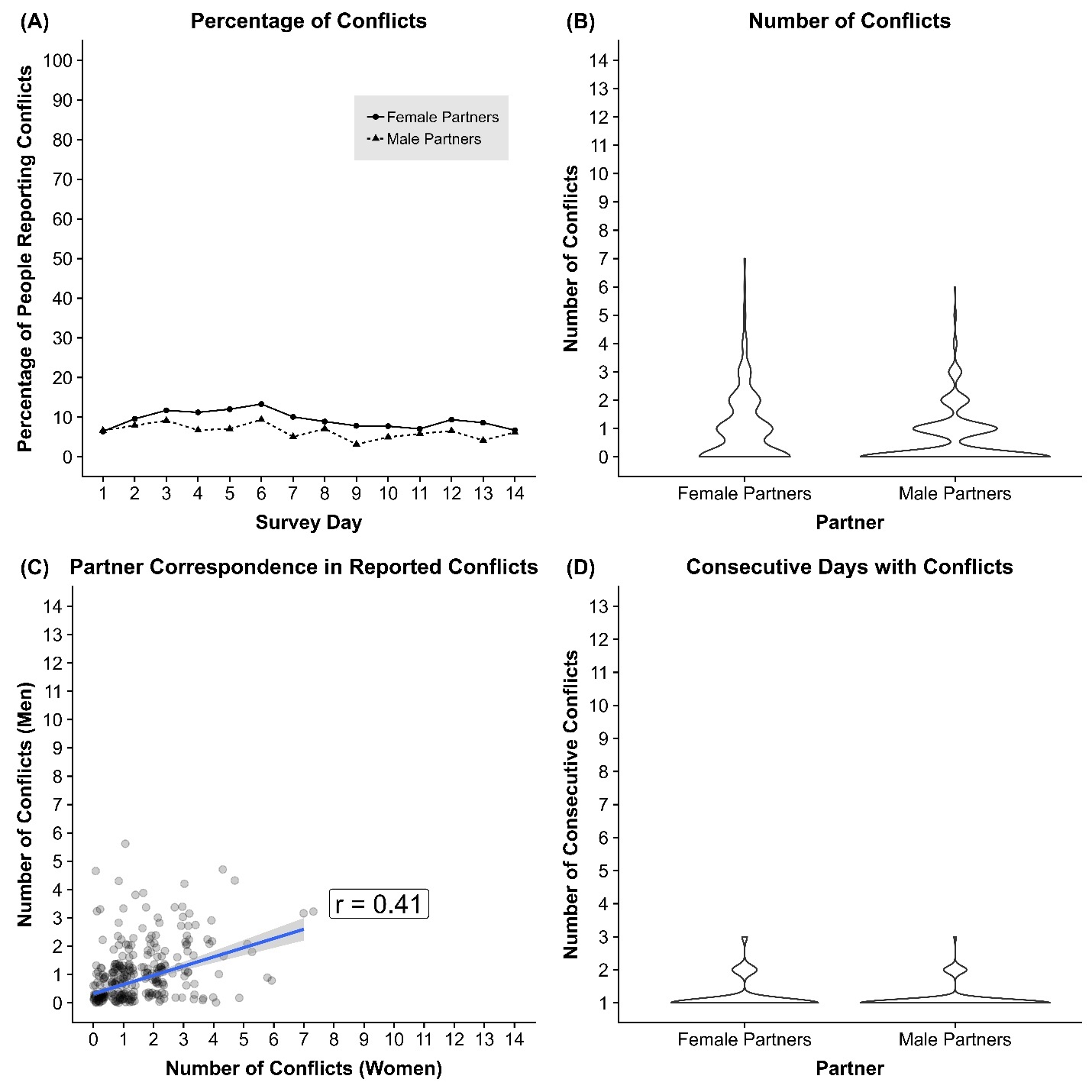


*Note:* Dots in Panel (C) are jittered to avoid overplotting.

**8. References**

References

Curran, P. J., Howard, A. L., Bainter, S. A., Lane, S. T., & McGinley, J. S. (2014). The

separation of between-person and within-person components of individual change over

time: A latent curve model with structured residuals. *Journal of Consulting and Clinical*

*Psychology, 82,* 879-894. https://doi.org/10.1037/a0035297

Duncan, G. J., Engel, M., Claessens, A., & Dowsett, C. J. (2014). Replication and robustness in

developmental research. *Developmental Psychology, 50*, 2417–2425. https://doi.org/10.1037/a0037996

Enders, C. K. (2011). Analyzing longitudinal data with missing values. *Rehabilitation*

*Psychology, 56,* 267-288. https://doi.org/10.1037/a0025579

Gottman, J. M., & Krokoff, L. J. (1989). Marital interaction and satisfaction: A longitudinal

view. *Journal of Consulting and Clinical Psychology, 57,* 47-52.

https://doi.org/10.1037/0022-006X.57.1.47

Graham, J. W. (2003). Adding missing-data-relevant variables to FIML-based structural equation

models. *Structural Equation Modeling: A Multidisciplinary Journal, 10,* 80-100.

https://doi.org/10.1207/S15328007SEM1001_4

Herzberg, P. Y., & Sierau, S. (2010). Das Konfliktlösungsstil-Inventar für Paare (KSIP):

Psychometrische Eigenschaften und Validierung der autorisierten deutschsprachigen

Übersetzung [The German version of the Conflict Resolution Styles Inventory (CRSI):

Psychometric properties and validation of the authorized German version]. Diagnostica,

56, 94-107. Doi: 10.1026/0012-1924/a000014

Johnson, M. D., Cohan, C. L., Davila, J., Lawrence, E., Rogge, R. D., Karney, B. R., … &

Bradbury, T. N. (2005). Problem-solving skills and affective expressions as predictors of

change in marital satisfaction. *Journal of Consulting and Clinical Psychology, 73*, 15-27.

https://doi.org/10.1037/0022-006X.73.1.15

Karney, B. R., & Bradbury, T. N. (1997). Neuroticism, marital interaction, and the trajectory of

marital satisfaction. *Journal of Personality and Social Psychology, 72,* 1075-1092.

https://doi.org/10.1037/0022-3514.72.5.1075

Little, T. D. (2013). *Longitudinal structural equation modeling*. New York: The Guilford Press.

McArdle, J. J., & Epstein, D. (1987). Latent growth curves within developmental structural

equation models. *Child Development, 58,* 110-133. https://doi.org/10.2307/1130295

Muthén, L. K., & Muthén, B. O. (1998-2017). *Mplus user’s guide* (8^th^ edition). Muthén &

Muthén.

Voelkle, M. C. (2009). Reconsidering the use of autoregressive latent trajectory (ALT) models.

*Multivariate Behavioral Research, 43,* 564-591. https://doi.org/10.1080/00273170802490665

Widaman, K. F., Ferrer, E., & Conger, R. D. (2010). Factorial invariance within longitudinal

structural equation models: Measuring the same construct across time. *Child*

*Development Perspectives, 4,* 10–18. doi:10.1111/j.1750-8606.2009.00110.x

1. The authors are grateful for the research assistance of Sabrina Brunner, Fabienne Fend, and Rahel Hütten. [↑](#footnote-ref-1)
